# Single‐Stranded Nucleic Acid Transmembrane Molecular Carriers Based on Positively Charged Helical Foldamers

**DOI:** 10.1002/advs.202400678

**Published:** 2024-05-16

**Authors:** Yunpeng Ge, Wencan Li, Jun Tian, Hao Yu, Zhenzhu Wang, Ming Wang, Zeyuan Dong

**Affiliations:** ^1^ State Key Laboratory of Supramolecular Structure and Materials College of Chemistry Jilin University 2699 Qianjin Street Changchun 130012 P. R. China; ^2^ Center for Supramolecular Chemical Biology Jilin University 2699 Qianjin Street Changchun 130012 P. R. China

**Keywords:** electrostatic interactions, helical foldamers, molecular carriers, RNA delivery, transmembrane transport

## Abstract

Transmembrane delivery of biologically active nucleic acids is an important process in cells and has inspired one to develop advanced drug delivery techniques. In this contribution, molecular‐level single‐stranded nucleic acid transmembrane carriers are reported based on 3.2 nm long Huc's foldamers (A^Orn^Q^3^Q^3^)_8_ and (^m^Q^3^Q^2^)_8_ with linearly and helically aligned positive charges, respectively. These two foldamers not only show very strong DNA affinity via electrostatic interactions but also discriminatively bind single‐stranded DNA (ss‐DNA) and double‐stranded DNA (ds‐DNA), corroborating the importance of precise charge arrangement in the electrostatic interactions. More importantly, these two foldamers are capable of efficiently transporting ss‐DNA across the lipid membranes, and the ss‐DNA transport activity of (A^Orn^Q^3^Q^3^)_8_ with linearly aligned charges is higher than that of (^m^Q^3^Q^2^)_8_ with helically aligned charges. Thus a type of novel single‐stranded nucleic acid transmembrane molecular carriers based on positively charged helical foldamers are introduced. Further, effective and enhanced expression in EGFP‐mRNA transfection experiments strongly demonstrates the potential of positively charged foldamers for RNA transmembrane transport and therapy.

## Graphical abstract

Single‐stranded nucleic acid transmembrane unimolecular carriers are developed by using 3.2 nm long helical foldamers with linearly or helically arranged positive charges.

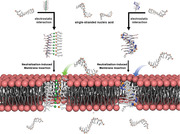

## 1 Introduction

Nucleic acids are fundamental biopolymers with unique structural characteristics and display pivotal roles in various biological processes in life systems. Meanwhile, transmembrane transport of nucleic acids is a key process in cells, which facilitates cell division, gene expression, and even intercellular communication by exchange of genetic information between cells.^[^ ^1^ ^]^ However, the structural features of nucleic acids, including biodegradability, negative charges, and hydrophilic backbones, strictly prevent free diffusion across the cell membranes.^[^ ^2^ ^]^ In fact, it is of biomedical importance to effectively deliver RNA therapeutic agents across the cell membranes to exert their intracellular functions. Thanks to the biocompatibility, low immunogenicity, and therapeutic function of nucleic acids,^[^ ^3^ ^]^ several typical delivery techniques based on RNA therapy have been developed to assist in nucleic acid transmembrane transport in the past few decades, such as lipid nanoparticles (LNP)^[^ ^4^ ^]^ and polymer‐based nanoparticles,^[^ ^5^ ^]^ viral or bacterial agents,^[^ ^6^ ^]^ and membrane‐penetrating peptides.^[^ ^7^ ^]^ In addition, inspired by the way that natural channels transport ions, only a few nucleic acid and protein transport models based on membrane‐spanning nanopores have been reported.^[^ ^8^ ^]^ Although these delivery techniques have greatly promoted nucleic acid therapy, developing new nucleic acid delivery strategies remains important.

**Figure 1:**
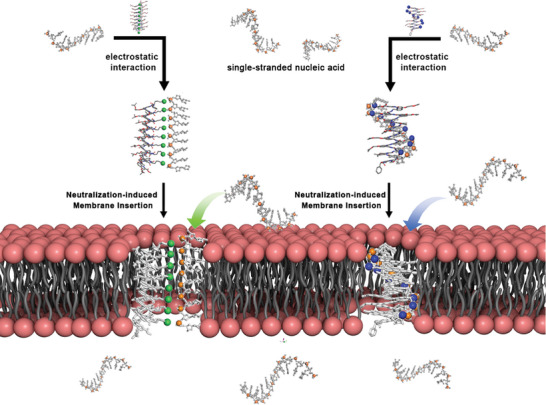
Schematic representation of single‐stranded nucleic acid transmembrane transport by using positively charged helical foldamers as molecular carriers.

**Figure 2:**
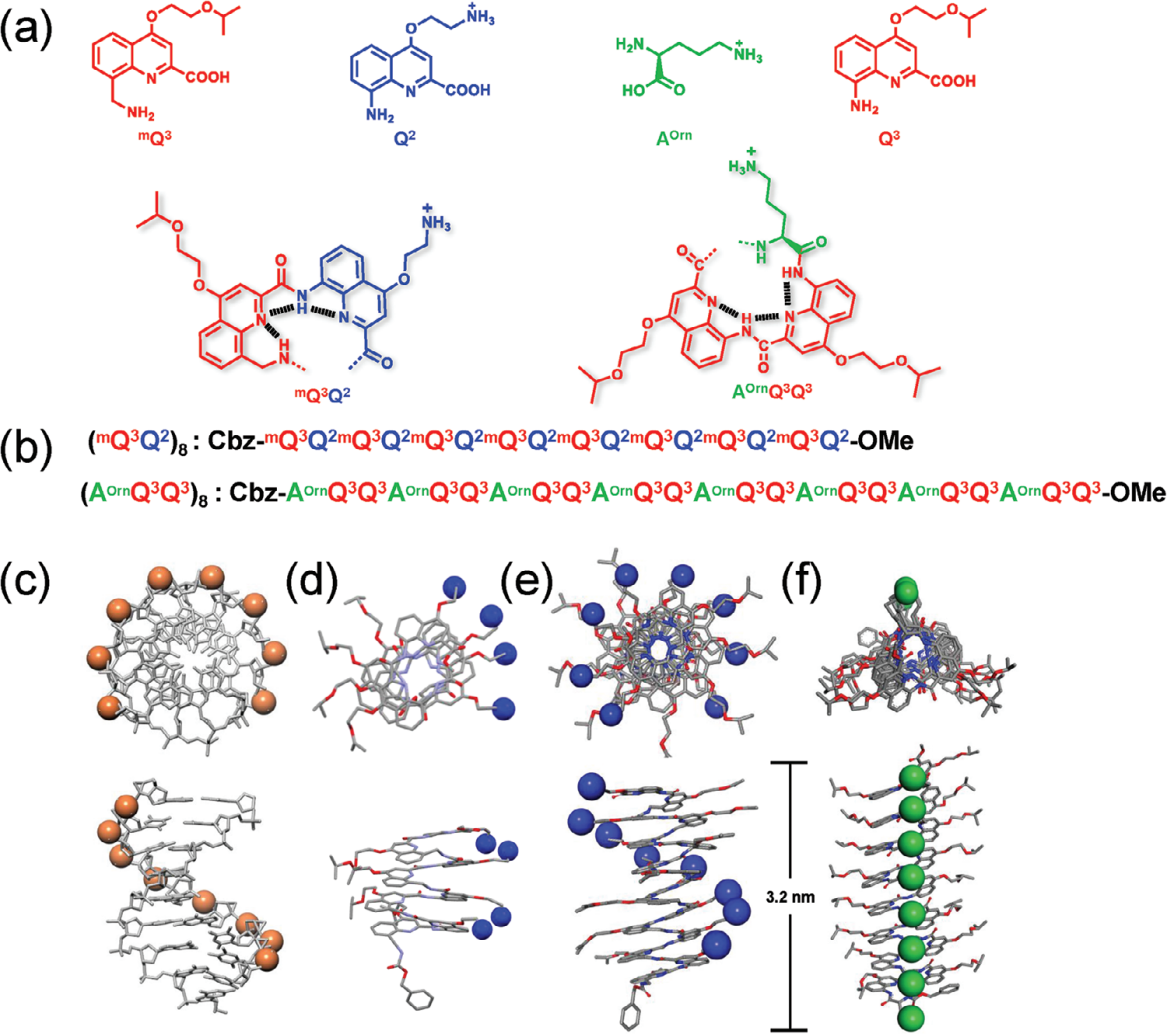
a) Chemical structures of monomers ^m^Q^3^, Q^2^, A^Orn^, Q^3^, ^m^Q^3^Q^2^, and A^Orn^Q^3^Q^3^ blocks. b) Sequences of (^m^Q^3^Q^2^)_8_ and (A^Orn^Q^3^Q^3^)_8_. c) Schematic representation, top view, and side view of models of the structure of an eight‐base‐pair ds‐DNA. d) Crystal structure of Cbz‐(^m^Q^3^Q^P2^)_4_‐OMe with the Boc groups hidden (P stands for Boc‐protected side chain). e) Simulation structures of (^m^Q^3^Q^2^)_8_ and (f) (A^Orn^Q^3^Q^3^)_8_ were obtained from their crystal structures.^[^ ^11^, ^12^ ^]^ Schemes show the distribution of the phosphorus atoms (orange) when viewed down the helix axis. Simulation and crystal structures are shown at the same scale as the stick representations except that the phosphorus atoms are shown as orange spheres and the nitrogen atoms are shown as blue or green spheres.

We wonder if it is possible to use molecular‐level carriers to achieve nucleic acid transmembrane delivery, of which this concept comes from the work on the chemical simulation of ion transport.^[^ ^9^ ^]^ Further, it is noteworthy that frequently used peptide transduction domains as well as cell‐penetrating peptides usually contain cationic peptide sequences of 8–16 amino acids in length.^[^ ^10^ ^]^ Herein, we introduce a type of single‐stranded nucleic acid transmembrane molecular carriers based on 3.2 nm long Huc's foldamers (A^Orn^Q^3^Q^3^)_8_ and (^m^Q^3^Q^2^)_8_ with linearly and helically aligned positive charges, respectively (**Figure** **1**). Based on Huc's foldamer structures, the side‐chain residues at the surface of (^m^Q^3^Q^2^)_8_ are expected to form a double‐helical array where the Q^2^ cationic side chains closely match the spatial position of phosphate groups in duplex B‐DNA (**Figure** **2**c–e). In contrast, the ornithine residues of helix (A^Orn^Q^3^Q^3^)_8_ are positioned to gain a linear array of cationic side chains at 3.5 Å interval that corresponds to the pitch of the aromatic helix (Figure 2f). We thus attempt to investigate electrostatic recognition and transmembrane delivery of single‐stranded nucleic acids by Huc's foldamers with different spatial arrangements of positively charged groups.

## 2 Results and Discussion

To achieve molecular carriers that might transport nucleic acids, helical foldamers were chosen because of their unique structure allowing for precise alignment of positive charges on their side chains. For example, Huc and coworkers developed aromatic oligoamide foldamers to finely replicate the negative charge surface of double‐stranded B‐DNA.^[^ ^11^ ^]^ Notably, the negatively charged phosphodiester groups with a regular inter‐planar distance of 3.5 Å are exposed on the outside of the B‐DNA structure. Furthermore, the length of eight‐charge arrangement of B‐DNA can effectively span the hydrophobic thickness of the lipid membrane. Therefore, foldamer with helically aligned eight oppositely positive charges on the side chains is desired as a nucleic acid transmembrane molecular carrier. At the same time, foldamer with linearly aligned eight positive charges on the side chains was also considered as a nucleic acid transmembrane molecular carrier.^[^ ^12^ ^]^ To obtain these two foldamers (Figure 2), we synthesized the desired monomers Q^2^, Q^3^, and ^m^Q^3^ following the synthesis schemes in supporting information. Q^2^ and Q^3^ were prepared by using an 8‐nitro‐2‐quinolinecarboxylate precursor via the Mitsunobu reaction.^[^ ^11^, ^12^, ^13^ ^]^ Monomer ^m^Q^3^ was obtained via an 8‐methyl‐4‐hydroxy‐2‐quinolinecarboxylate intermediate. The desired 8‐aminomethyl function was synthesized by stepwise reacitions including bromination, azide, and reduction. Especially, the 2‐isopropoxyethoxyl group was selected as the relatively water‐soluble side chain in monomers ^m^Q^3^. Positively charged ammonium groups were introduced as a Boc‐protected form in monomer Q^2^. The side chain length was adjusted to make the size of the overall foldamer helix matchable with B‐DNA. The main chain aromatic amines were protected with a benzyloxucarbonyl group (Cbz) while the aliphatic amines on the side‐chain of Q^2^ and A^Orn^ were protected with a Boc group to allow selective side‐chain deprotection at the end of the foldamer synthesis. A segment doubling approach was followed in which the Boc‐protected forms of CbzNH‐(^m^Q^3^Q^2^)_n_‐CO_2_H and H_2_N‐(^m^Q^3^Q^2^)_m_‐CO_2_Me were coupled to yield CbzNH‐(^m^Q^3^Q^2^)_n+m_‐CO_2_Me. A series of (^m^Q^3^Q^2^)_n_ oligomers with n = 2, 4, and 8 were synthesized in this way, and then Boc groups were deprotected by using trifluoroacetic acid. The coupling of the aromatic units was carried out by applying the conditions recently developed by Beutner^[^ ^14^ ^]^ with the use of *N*,*N*,*N*′,*N*′‐tetramethylchloroformamidinium hexafluorophosphate (TCFH) and N‐methylimidazole (NMI), without resorting to the use of acid chloride activation, Cbz‐A^Orn^‐ornithine could be coupled to an 8‐aminoquinoline monomer to give A^orn^Q^3^ in one step. Similarly, A^Orn^Q^3^‐CO_2_H coupled to an 8‐aminoquinoline monomer to give A^Orn^Q^3^Q^3^ trimer. The same segment doubling approach was followed to give fully Boc‐protected (A^Orn^Q^3^Q^3^)_8_. Consequently, two kinds of foldamers, (^m^Q^3^Q^2^)_8_ with helically aligned eight positive charges and (A^Orn^Q^3^Q^3^)_8_ with linearly aligned eight positive charges, were straightforwardly synthesized and fully characterized (Figure 2b). The crystal structure of Cbz‐(^m^Q^3^Q^P2^)_4_‐OMe (Figure 2d) supports the helical array of positively charged side chains on the surface of foldamer (^m^Q^3^Q^2^)_8_ with near 3.5 Å distance between two adjacent positive charges,^[^ ^11^ ^]^ similar to the base‐pair distance of B‐DNA (Figure 2c). These two foldamers with a molecular length of 3.2 nm can cross the hydrophobic region of the lipid membranes (Figure 2e,f).

**Figure 3:**
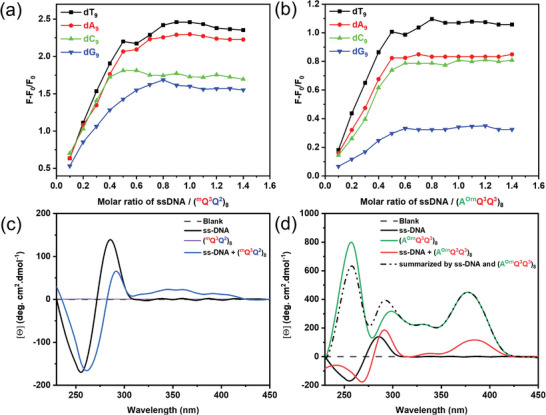
The plots of relative FL intensity of a) (^m^Q^3^Q^2^)_8_ (2 µm) and b) (A^Orn^Q^3^Q^3^)_8_ (2 µm) in 10 mm Tris·HCl, 10 mm NaCl, pH 7.4 upon titrating 0–1.4 equivalent amounts of dT_9_, dA_9_, dC_9_, and dG_9_. Zeta potential distribution graphs of c) 2.5 µm (^m^Q^3^Q^2^)_8_ and d) 2.5 µm (A^Orn^Q^3^Q^3^)_8_ with 2.5 µm dG_9_ in 10 mm Tris·HCl, 10 mm NaCl, pH 7.4. CD titration spectra of 10 µm ss‐DNA dT_13_ upon addition of (c) 5 µm (^m^Q^3^Q^2^)_8_ and d) 5 µM (A^Orn^Q^3^Q^3^)_8_ upon addition of 10 µm ss‐DNA dT_13_ in 10 mm Tris·HCl, 10 mm NaCl, pH 7.4.

Foldamers (^m^Q^3^Q^2^)_8_ and (A^Orn^Q^3^Q^3^)_8_ both showed an absorption band at 300–430 nm. Under 360 nm excitation, the maximum emission peaks of (^m^Q^3^Q^2^)_8_ and (A^Orn^Q^3^Q^3^)_8_ are found to be 488 and 465 nm, respectively (Figure S2, Supporting Information). Next, the binding properties of foldamers (^m^Q^3^Q^2^)_8_ and (A^Orn^Q^3^Q^3^)_8_ to single‐stranded DNA (ss‐DNA) were studied by fluorescence titrations, respectively. With gradual addition of various ss‐DNA (dT_9_ (5′‐TTT TTT TTT‐3′), dA_9_ (5′‐AAA AAA AAA‐3′), dC_9_ (5′‐CCC CCC CCC‐3′), and dG_9_ (5′‐GGG GGG GGG‐3′), the fluorescence intensity of foldamer (^m^Q^3^Q^2^)_8_ (2.0 µm) at 488 nm remarkably increases, even though the concentration of ss‐DNA is as low as to 0.2 µm (Figure S3, Supporting Information). Similarly, the fluorescence intensity of foldamer (A^Orn^Q^3^Q^3^)_8_ (2.0 µm) at 465 nm significantly enhances with the addition of various ss‐DNAs (Figure S4, Supporting Information). The fluorescence titration experiments indicate that the affinity between foldamers and ss‐DNAs is beyond 10^6^ M^−1^ (Figure S5, Supporting Information). The change in fluorescence intensity was caused by electrostatic interactions between foldamers and ss‐DNAs (**Figure** **3**a,b). The Zeta potential experiments were utilized to underpin electrostatic interactions between foldamers and ss‐DNAs. In the presence of foldamers, the negative potential of ss‐DNA was neutralized partially (Figures S6 and S7, Supporting Information).

Additionally, circular dichroism (CD) spectroscopy was used to investigate the complexation of foldamers and ss‐DNA. As seen in the CD spectrum (Figure 3c), by mixing achiral (^m^Q^3^Q^2^)_8_ and dT_13_ (5′‐ TTT TTT TTT TTT T‐3′), a dramatic decrease in CD intensity at 285 nm accompanying obvious redshift to 292 nm was observed, and a new signal band at 300–410 nm appeared. The achiral (^m^Q^3^Q^2^)_8_ is dynamic and undergoes helicity induction by diastereoselective binding with chiral ss‐DNA (Figure S8a, Supporting Information). For right‐handed foldamer (A^Orn^Q^3^Q^3^)_8_,^[^ ^12a^ ^]^ the presence of ss‐DNA dT_13_ caused obvious redshift from 378 to 385 nm as well as the drop in CD intensity of the positive band at 378 nm (Figure 3d; Figure S8b, Supporting Information). This drop in the CD intensity might be mainly due to the formation of a slightly water‐soluble ssDNA‐(A^Orn^Q^3^Q^3^)_8_ complex through charge neutralization. To observe the complexation of ss‐DNA and foldamers, AFM experiments were carried out. The morphologies of (^m^Q^3^Q^2^)_8_ and (A^Orn^Q^3^Q^3^)_8_ were found as assembled nanowires with profile height of 2.3 ± 0.1 nm in the absence of ssDNA dT_9_ (Figure S10a–d, Supporting Information). Importantly, in the presence of dT_9_, (^m^Q^3^Q^2^)_8_ exhibited very uniform nanowires with a profile height of 2.7 nm, which is consistent with the expected complex structure (Figure S10b, Supporting Information). However, the morphology of (A^Orn^Q^3^Q^3^)_8_ with dT_9_ displayed uniform nanowires with a profile height of 5.2 nm, which is twofold higher than that of the expected complex structure, suggesting further controllable self‐assembly caused by the hydrophobic effect of isopropyl groups in the case (Figure S10e, Supporting Information). These results indicate the precise spatial arrangement of positive charges in synthetic foldamers leads to strong ss‐DNA recognition ability as well as highly ordered complex structures in water. Similarly, the binding abilities of foldamers (^m^Q^3^Q^2^)_8_ and (A^Orn^Q^3^Q^3^)_8_ to double‐stranded DNA were measured by various techniques. In order to ensure the formation of a double‐stranded DNA structure in the case, a short self‐complementary sequence (5′‐CCA GTA CTG G‐3′) with a relatively high melting temperature (Tm >25 °C) was chosen.^[^ ^15^ ^]^ As seen in CD spectra (Figure S9, Supporting Information), the CD peak at 290 nm significantly decreased in intensity upon the addition of (^m^Q^3^Q^2^)_8_. However, no obvious CD signals at 300–410 nm were detectable while mixing foldamer (^m^Q^3^Q^2^)_8_ and ds‐DNA, suggesting very weak chiral induction between ds‐DNA and (^m^Q^3^Q^2^)_8_. Additionally, the CD spectra of ds‐DNA and (A^Orn^Q^3^Q^3^)_8_ mixture represented slight redshifts, in which the peak of ds‐DNA at 290 nm moves to 293 nm and the helicity peak of (A^Orn^Q^3^Q^3^)_8_ at 378 nm shifts to 385 nm. These observations suggest that the binding behaviors between ds‐DNA and foldamers ((^m^Q^3^Q^2^)_8_ and (A^Orn^Q^3^Q^3^)_8_) do happen via electrostatic interactions.^[^ ^16^ ^]^ The AFM experiments of foldamers and ds‐DNA (5′‐CCA GTA CTG G‐3′) further demonstrate the discrimination recognition of positive charge arrangement on ds‐DNA. The helical arrangement of positive charges gave rise to nanoparticle morphology of (^m^Q^3^Q^2^)_8_ and ds‐DNA (Figure S11a, Supporting Information). However, as shown in AFM (Figure S11b, Supporting Information), the nanosheet‐like morphologies with uniform 2.2 nm profile height were observed by mixing ds‐DNA and (A^Orn^Q^3^Q^3^)_8_. Considering the structural feature of ds‐DNA, we employ UV thermal melting experiments to monitor the electrostatic interactions between positively charged foldamers and ds‐DNA. The T_m_ of ds‐DNA (5′‐AGC CTA GGA TAA GAG‐3′) was measured to be 47.4 °C. Very surprisingly, as observed by the melting temperature curves (Figure S12, Supporting Information), T_m_ values of ds‐DNA change to 44.7 and 44.3 °C in the presence of (^m^Q^3^Q^2^)_8_ and (A^Orn^Q^3^Q^3^)_8_, respectively. As compared, the branched polyethyleneimine (M.W. 600) exhibits a mild thermal stabilization ability for ds‐DNA with a T_m_ value of 48.0 °C (Figure S13, Supporting Information). This indicates that precisely designed electrostatic interactions can weaken the stability of ds‐DNA.

**Figure 4:**
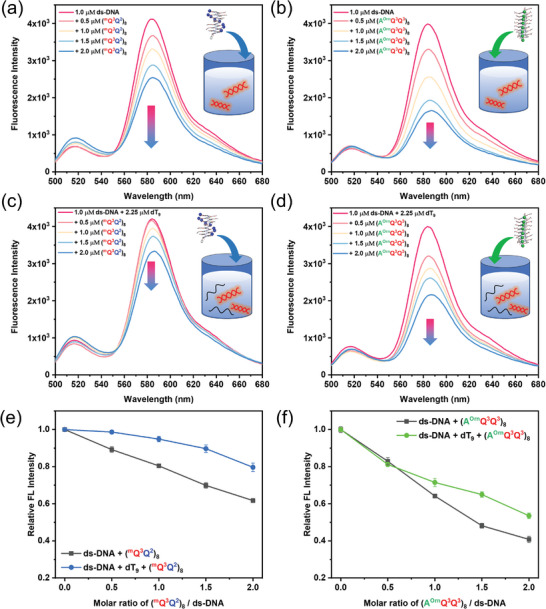
a) The fluorescence spectra of ds‐DNA at the concentration of 1.0 µm upon titrating 0–2.0 µm (^m^Q^3^Q^2^)_8_. b) The fluorescence spectra of ds‐DNA at the concentration of 1.0 µm upon titrating 0–2.0 µm (A^Orn^Q^3^Q^3^)_8_. c) The fluorescence spectra of ds‐DNA (1.0 µm) and ss‐DNA (2.25 µm) upon titrating 0–2.0 µm (^m^Q^3^Q^2^)_8_. d) The fluorescence spectra of ds‐DNA (1.0 µm) and ss‐DNA (2.25 µm) upon titrating 0–2.0 µm (A^Orn^Q^3^Q^3^)_8_. The plots of relative FL intensity of e) (^m^Q^3^Q^2^)_8_ and f) (A^Orn^Q^3^Q^3^)_8_.

Owing to the difference in the structures of ss‐DNA and ds‐DNA, their selective recognition by using foldamers (^m^Q^3^Q^2^)_8_ and (A^Orn^Q^3^Q^3^)_8_ was investigated. The single‐stranded dT_9_ was used as ss‐DNA that has no fluorescence at 480 nm excitation. The fluorescently labeled ds‐DNA composed of self‐complementary sequence 5′‐FAM‐CCA GTA CTG G‐TAMRA‐3′ was employed in selective recognition experiments (Figure S14, Supporting Information). The fluorescence intensity at 580 nm from TAMRA moiety in this self‐complementary sequence was observed under 480 nm excitation (**Figure** **4**), which represents the formation of a double‐stranded helix accompanied by the occurrence of Förster Resonance Energy Transfer (FRET). With the gradual addition of foldamer (^m^Q^3^Q^2^)_8_ or (AO^rn^Q^3^Q^3^)_8_, the fluorescence intensity at 580 nm significantly decreased (Figure 4a,b), indicating that both foldamers (^m^Q^3^Q^2^)_8_ and (A^Orn^Q^3^Q^3^)_8_ can efficiently bind ds‐DNA, and even potentially unwind the double helix structure of DNA. When ss‐DNA dT_9_ with the same negative charges was added into the competition experiment (Figure 4c), the fluorescence intensity of labeled ds‐DNA decreased very slightly rather than significantly in the presence of (^m^Q^3^Q^2^)_8_, indicating that foldamer (^m^Q^3^Q^2^)_8_ preferentially binds ss‐DNA rather than ds‐DNA (Figure 4e). However, the fluorescence intensity of labeled ds‐DNA with or without ss‐DNA (dT_9_) remains almost unchanged in the presence of less than one equivalent of foldamer (A^Orn^Q^3^Q^3^)_8_ (Figure 4d), suggesting that foldamer (A^Orn^Q^3^Q^3^)_8_ preferentially recognizes ds‐DNA rather than ss‐DNA (Figure 4f). When the amount of (A^Orn^Q^3^Q^3^)_8_ further increases, the recognition of foldamer to ss‐DNA subsequently takes place, as evidenced by that the fluorescence intensity of labeled ds‐DNA with ss‐DNA dT_9_ is higher than that without ss‐DNA dT_9_. These observations demonstrated that, in the mixture solution of ss‐DNA and ds‐DNA, foldamer (^m^Q^3^Q^2^)_8_ with helically arranged positive charges selectively recognizes ss‐DNA, whereas foldamer (A^Orn^Q^3^Q^3^)_8_ with linearly arranged positive charges preferentially binds to ds‐DNA (Figure 4e,f). This phenomenon on discriminative recognition of single‐stranded and double‐stranded DNA is interesting and reasonable since the linearly aligned charges on the surface of helical foldamer (A^Orn^Q^3^Q^3^)_8_ could contact with ds‐DNA over a larger area than helically aligned charges of (^m^Q^3^Q^2^)_8_. These results also indicate that spatial electrostatic interactions between oppositely charged groups are important in nucleic acid recognition.^[^ ^17^ ^]^

**Figure 5:**
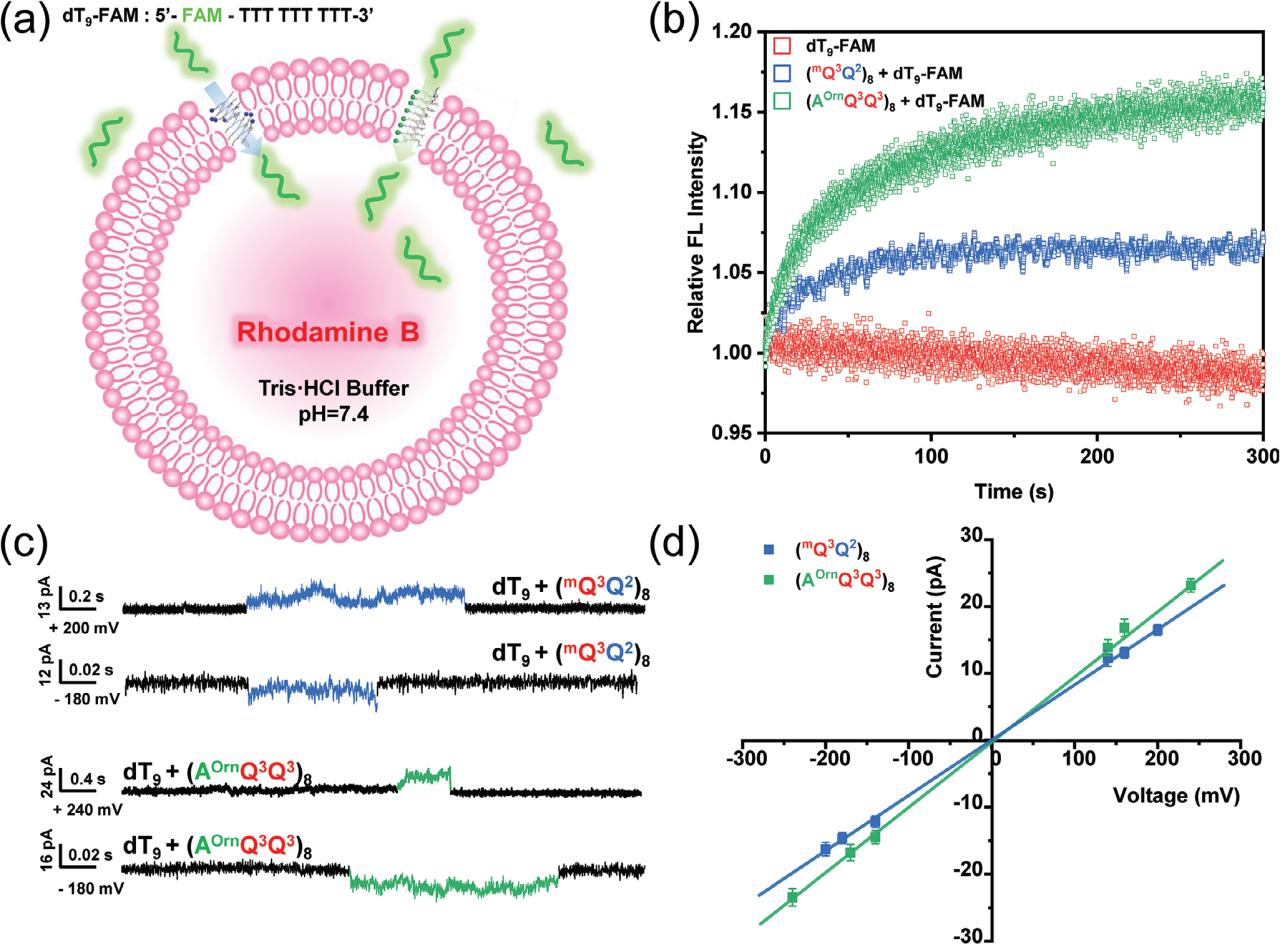
a) Schematic of vesicle‐based kinetic assay. b) Normalized dT_9_‐FAM transport activity of (^m^Q^3^Q^2^)_8_ and (A^Orn^Q^3^Q^3^)_8_ at 1 µm. c) Current traces were recorded for (^m^Q^3^Q^2^)_8_ and (A^Orn^Q^3^Q^3^)_8_ with dT_9_ at different voltages. d) Current–voltage relationship of (^m^Q^3^Q^2^)_8_ and (A^Orn^Q^3^Q^3^)_8_ from symmetric BLM experiments.

Taking advantage of the strong binding ability and electrostatic neutralization of foldamers (^m^Q^3^Q^2^)_8_ and (A^Orn^Q^3^Q^3^)_8_ to single‐stranded DNA, we investigated the ss‐DNA transmembrane transport functions of foldamers by vesicle‐based kinetic assay (**Figure** **5**a).^[^ ^18^ ^]^ We evaluated the stability of lipid membranes in the presence of the ssDNA/foldamer complexes by molecular probe carboxyfluorescein (CF) and found that the rupture of large lamellar vesicles (LUVs) can be avoided at the experimental conditions (Figure S15a, Supporting Information).^[^ ^18b^ ^]^ As observed, the fluorescence intensity of CF monitoring at 520 nm increased by the addition of foldamers (1.0 µm), which indicated that positively charged foldamers were not conducive to the stability of the membrane. In particular, foldamer (^m^Q^3^Q^2^)_8_ with helically arranged positive charges was found more destructive to the stability of the membrane than foldamer (A^Orn^Q^3^Q^3^)_8_ with linearly arranged positive charges (Figure S15b,c, Supporting Information). When the mixture of foldamers and dT_9_ was added, the fluorescence intensity of the mixture solution was almost kept unchanged, indicating that the lipid membranes were stable under the mixture conditions. As observed in the emission spectra (Figure S16a, Supporting Information), the fluorescence intensity of dT_9_‐FAM at 570 nm did not enhance in the absence of foldamers, suggesting that negatively charged dT_9_‐FAM itself could not pass through the lipid membranes to achieve FRET. When the melittin was added to the experimental system, no significant change was found in fluorescence intensity, indicating no FRET between the dT_9_‐ FAM and rhodamine B at the tested concentration.

After checking the control experiments without FRET, the ss‐DNA transport function was measured. When the mixture of foldamer and dT_9_‐FAM was added to the vesicle solution, the fluorescence intensity at 570 nm significantly increased, suggesting that foldamer could carry dT_9_‐FAM to cross the lipid membranes and realize FRET (Figure S16b, Supporting Information). After the addition of melittin, LUV rupture led to sample dilution and the loss of FRET. Surprisingly, in the presence of foldamers, remarkable increases in fluorescence intensity over time were observed (Figure 5b). In contrast to the references such as branched polyethyleneimine (M.W. 600) and linear polyethyleneimine (average Mn: 5000, PDI < 1.2), foldamers (^m^Q^3^Q^2^)_8_ and (A^Orn^Q^3^Q^3^)_8_ both exhibited important transmembrane transport ability over ss‐DNA (Figure S18, Supporting Information). This result indicates that both foldamers (^m^Q^3^Q^2^)_8_ and (A^Orn^Q^3^Q^3^)_8_ are capable of accomplishing transmembrane delivery of ss‐DNA. Moreover, foldamer (A^Orn^Q^3^Q^3^)_8_ with linearly aligned charges exhibits ss‐DNA transport ability stronger than (^m^Q^3^Q^2^)_8_ with helically aligned charges. In addition, symmetric bilayer lipid membrane (BLM) experiments were carried out to further underpin the ss‐DNA transport functions of foldamers. Helical foldamers alone displayed a big current in a symmetrical solution (Figure S19a, Supporting Information), which indicates that positively charged foldamers can cause the rupture of the planer lipid membrane. As shown in Figure 5c, both foldamers (^m^Q^3^Q^2^)_8_ and (A^Orn^Q^3^Q^3^)_8_ exhibited clear current signals and current traces of ss‐DNA (dT_9_) (Figure S19, Supporting Information), suggesting the charge transmembrane delivery through foldamer‐assisting ss‐DNA transport. Average current values at different voltages were obtained by Gaussian formula fitting. Furthermore, the conductance of (A^Orn^Q^3^Q^3^)_8_ calculated from *I*–*V* plots is up to 98.7 pS, which is larger than the conductance (83.0 pS) of (^m^Q^3^Q^2^)_8_ under the identical conditions (Figure 5d). According to the conductance values of (A^Orn^Q^3^Q^3^)_8_ and (^m^Q^3^Q^2^)_8_, their ss‐DNA transport rates are calculated to be 7.70 × 10^6^ and 6.47 × 10^6^ dT_9_ s^−1^ at 100 mV, respectively. As observed, the ss‐DNA transport activity of (A^Orn^Q^3^Q^3^)_8_ with linearly aligned charges is indeed higher than that of (^m^Q^3^Q^2^)_8_ with helically aligned charges, according to the result from vesicle‐based kinetic analyses. The difference in ss‐DNA transport activity between (A^Orn^Q^3^Q^3^)_8_ and (^m^Q^3^Q^2^)_8_ might be due to the different arrangement of positive charges, and the helical charge arrangement is not conducive to membrane binding. This not only corroborates the importance of precise spatial arrangement of charges in electrostatic interactions but also develops a feasible approach for transporting single‐stranded DNA across the lipid membranes.

**Figure 6:**
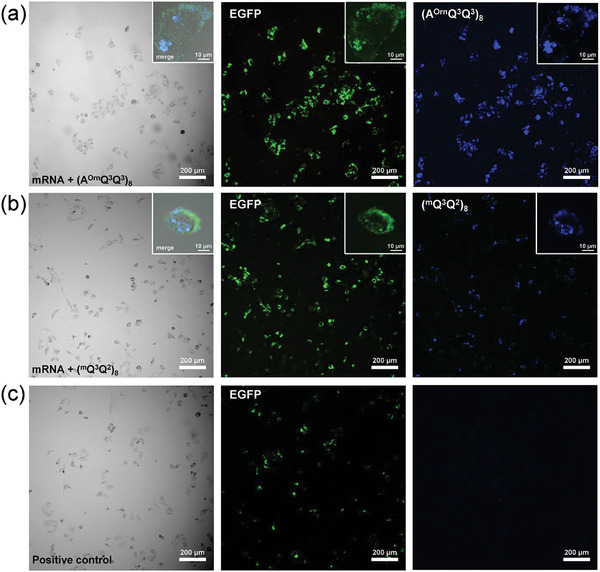
Confocal laser scanning microscopy images (10×) of Human Umbilical Vein Endothelial Cells (HUVEC) after transfection with a) (A^Orn^Q^3^Q^3^)_8_ and b) (^m^Q^3^Q^2^)_8_ carrying EGFP‐mRNA at the concentration of 10 µm. Insets focus on single cells (40×). c) Confocal laser scanning microscopy images (10×) of positive control (1 µL Hieff Trans Liposomal Transfection Reagent carrying EGFP‐mRNA). (405 nm laser excitation for foldamers (blue fluorescence), 488 nm laser excitation for EGFP (green fluorescence)).

To test the potential of foldamers acting as the mRNA carriers in cells, the cytotoxicity of foldamers was primarily examined by treating different concentrations on Human Umbilical Vein Endothelial Cells and measuring cell viability. After incubation for 24 h, both foldamers (^m^Q^3^Q^2^)_8_ and (A^Orn^Q^3^Q^3^)_8_ did not exhibit obvious cytotoxicity (Figure S20, Supporting Information). In light of the result from the cytotoxicity assay, the concentration of 10 µm for foldamers was used because it does not cause significant toxicity effects in EGFP‐mRNA transfection experiments in HUEVC. Both (^m^Q^3^Q^2^)_8_ and (A^Orn^Q^3^Q^3^)_8_ show efficient and enhanced EGFP‐mRNA expression compared to the positive control experiment (Hieff Trans Liposomal Transfection Reagent (Yaesen)) in laser scanning confocal microscopy (**Figure** **6**). As shown in the inserted merged illustration, the positively charged helical foldamers (blue fluorescence) are widely distributed in individual cells with EGFP (green fluorescence) and at the cell edges. These results indicate that synthetic foldamers with precise spatial arrangement of positive charges are not only capable of effectively facilitating the transmembrane transport of RNA but also could be molecular‐level gene carriers for promising mRNA delivery and therapy.

## 3 Conclusion

In summary, we design and synthesize two different types of Huc's foldamers with linearly and helically aligned positive charges to spatially match the negative charges of nucleic acids. Surprisingly, these two foldamers possess strong DNA recognition ability via electrostatic interactions, and discriminative binding of single‐stranded and double‐stranded DNA has been achieved by precise spatial arrangement of positive charges in synthetic foldamers. In the coexistence of ss‐DNA and ds‐DNA, foldamer (^m^Q^3^Q^2^)_8_ with helically aligned charges selectively recognizes ss‐DNA, whereas foldamer (A^Orn^Q^3^Q^3^)_8_ with linearly aligned charges preferentially binds to ds‐DNA. Moreover, the melting temperature experiments of ds‐DNA suggest that precisely designed electrostatic interactions would destroy hydrogen bonding interactions of base pairs of ds‐DNA, resulting in the destabilization of the double‐stranded structure of ds‐DNA. Importantly, both foldamers are capable of transporting ss‐DNA across the lipid membranes. More importantly, synthetic foldamers with precise spatial arrangement of positive charges are capable of effectively facilitating the transmembrane delivery of RNA, as illustrated by the observations in EGFP‐mRNA transfection experiments. This preliminary study not only develops an important nucleic acid delivery strategy but also reports a type of molecular‐level gene transmembrane carriers for future applications in RNA delivery and therapy.

## Conflict of Interest

The authors declare no conflict of interest.

## Supporting information

Supporting Information

## Data Availability

The data that support the findings of this study are available in the Supporting Information of this article.
