# Supplementary material for: Single‐Stranded Nucleic Acid Transmembrane Molecular Carriers Based on Positively Charged Helical Foldamers
Source: Adv Sci (Weinh). 2024 May 16;11(28):2400678. doi: 10.1002/advs.202400678 (PMC11267351; doi:10.1002/advs.202400678)
Supplement: Supplementary file 1 — Supporting Information [file ADVS-11-2400678-s001.docx]

Supporting Information

Single-Stranded Nucleic Acid Transmembrane Molecular Carriers Based on Positively Charged Helical Foldamers

Yunpeng Ge,^1, 2^ Wencan Li,^1, 2^ Jun Tian,^1, 2^ Hao Yu,^1^ Zhenzhu Wang,^1, 2^ Ming Wang,^1^ and Zeyuan Dong^1, 2, *^

1State Key Laboratory of Supramolecular Structure and Materials, College of Chemistry, Jilin University, Changchun 130012, China

2 Center for Supramolecular Chemical Biology, Jilin University, Changchun 130012, China

*Corresponding Author: Zeyuan Dong Email Address: [zdong@jlu.edu.cn](mailto:zdong@jlu.edu.cn)

**Table of Contents**

General remarks and synthesis schemes.....................................................................................S2-S4

X-Ray crystal data and molecular modeling..................................................................................S4-S5

Fluorescence titration spectrum....................................................................................................S5-S7

Zeta potential experiments............................................................................................................S8

CD experiments.........................................................................................................................S8-S9

Atomic Force Microscope images...............................................................................................S9-S10

UV Melting Analysis................................................................................................................S10-S11

Fluorescence recognition of single- and double-stranded oligonucleotides experiments..............S11-S12

Vesicle-based kinetic experiment..............................................................................................S12-S14

Bilayer lipid membrane (BLM) experiments.............................................................................S14-S15

Cytotoxicity assays........................................................................................................................S15-S16

EGFP-mRNA transfection imaging by laser scanning confocal microscope.................................S16-S18

^1^H NMR, ^13^C NMR and Mass spectra of purified new synthetic compounds.............................S18-S62

References......................................................................................................................................S62

**General remarks and synthesis schemes**

HPLC-purified single oligonucleotides dT_9_ (5’-TTT TTT TTT-3’), dC_9_ (5’-CCC CCC CCC-3’), dA_9_ (5’-AAA AAA AAA-3’), dG_9_ (5’-GGG GGG GGG-3’), dT_13_ (5’-TTT TTT TTT TTT T-3’), dT_9_-FAM (5’- FAM-TTT TTT TTT-3’), duplex DNA (5’-FAM-CCA GTA CTG G-TAMRA-3’), duplex DNA (5’-CCA GTA CTG G-3’), 15bp-duplex DNA (5’-AGC CTA GGA TAA GAG-3’) were purchased from Sangon Biotech. EGFP-mRNA was purchased from Absin. All the regents were obtained from commercial suppliers and used without further purification. N, N-dimethylformamide (DMF), acetonitrile (MeCN), N-Methylimidazole (NMI), N,N,N’,N’-tetramethylchloroformamidinium hexafluorophosphate (TCFH), triethylamine (TEA), chloroform (CHCl_3_) were distilled from CaH_2_ prior to use. Aqueous solution was prepared from MilliQ water. All the reactions were monitored by thin layer chromatography (TLC) and observed with ultraviolet light (UV), while column chromatography purifications were carried out via silica gel. ^1^H and ^13^C NMR spectra were recorded on the WNMR-I 400 or Bruker AVANCEⅢ 500. The solvents signals of CDCl_3_ and DMSO-d_6_ (Dimethyl sulfoxide-d_6_) for ^1^H NMR spectra were referenced at δ = 7.26 and 2.50 ppm, respectively. ^1^H NMR data are recorded in the order: chemical shift (ppm), multiplicity (s = singlet, d = doublet, t = triplet, m = multiplet, br = broad), number of protons. The solvents signals of CDCl_3_ and DMSO-d_6_ for ^13^C NMR spectrum were referenced at δ = 77.16 and 39.52 ppm, respectively. The solvent signals of CD_3_CN (Acetonitrile-d3) for ^1^H NMR and ^13^C NMR were referenced at δ = 1.94 ppm and 1.32, 118.26 ppm, respectively. The mass spectra were obtained on an HP1100EMD (electrospray ionization mass spectrometry, ESI MS).

**Synthesis schemes**


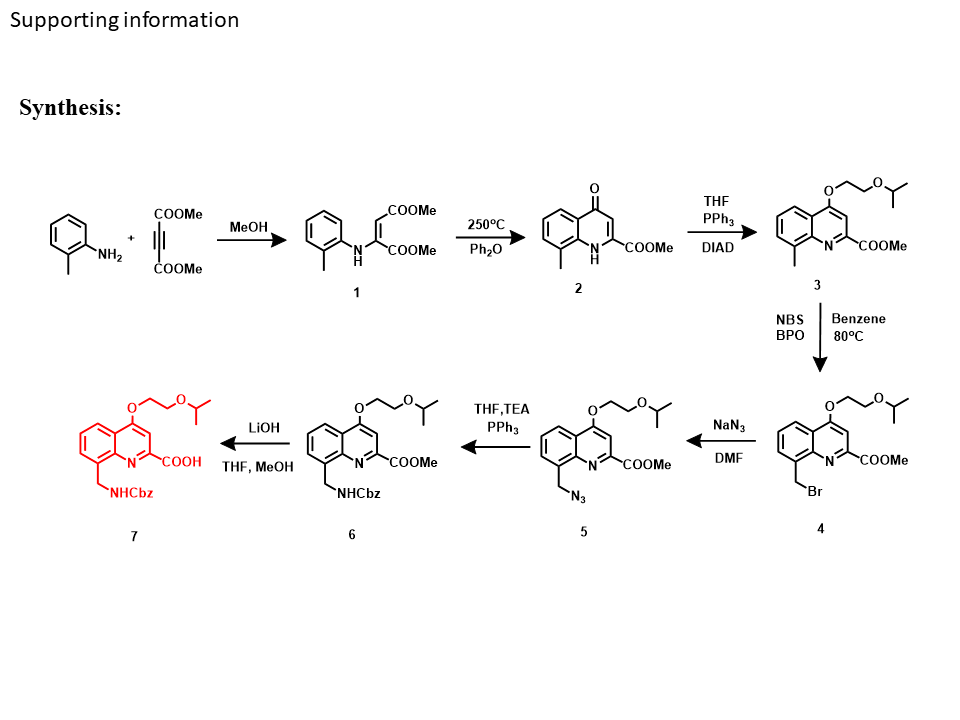
Preparation of monomer 7.


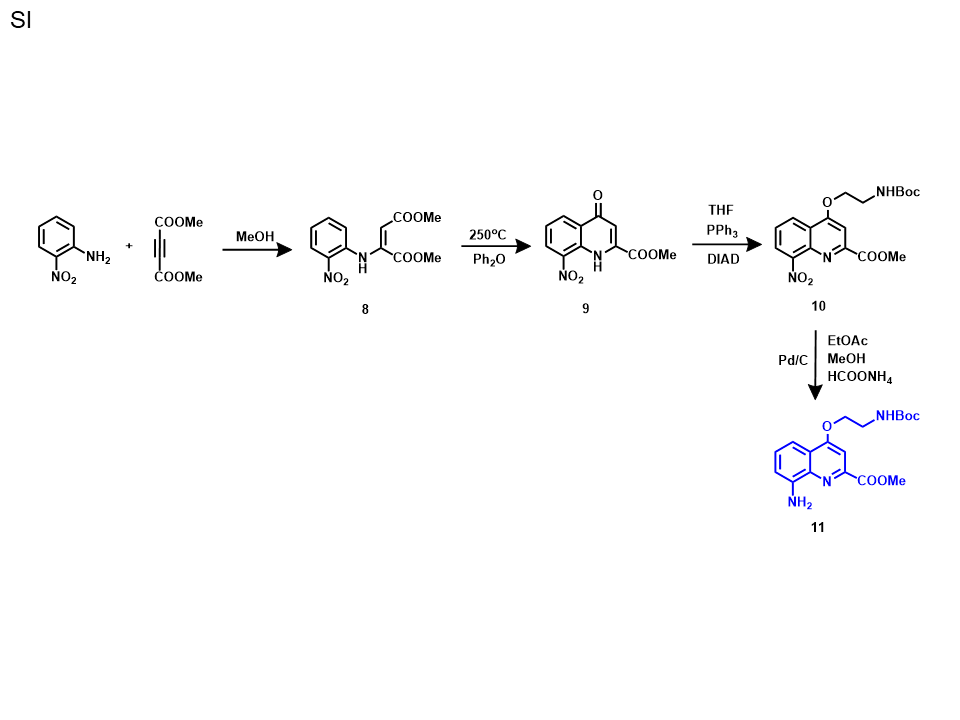
Preparation of monomer 11.


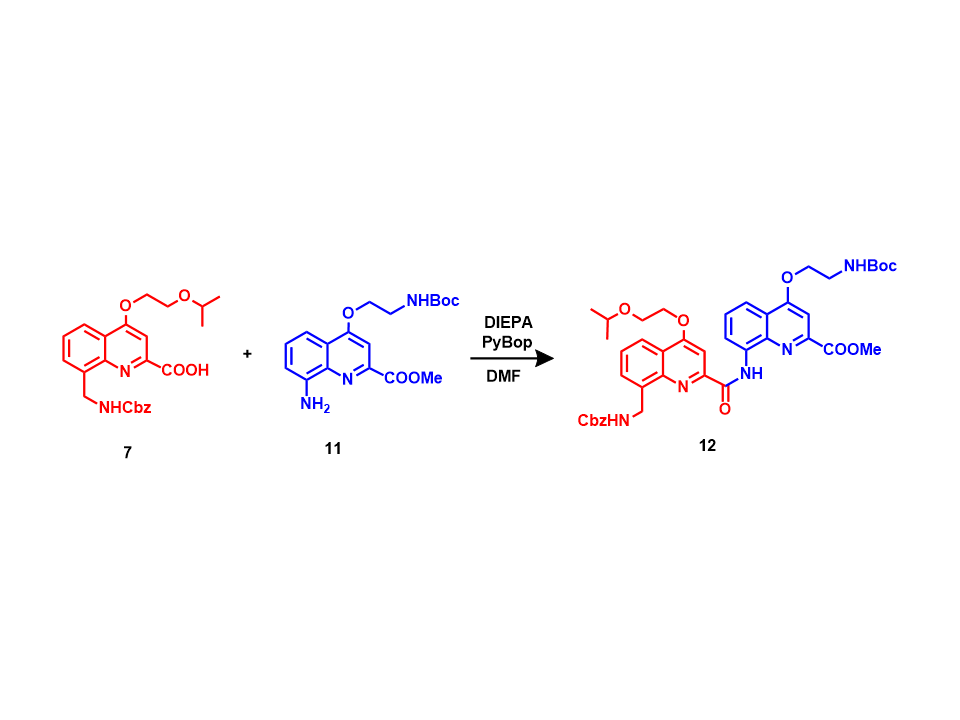


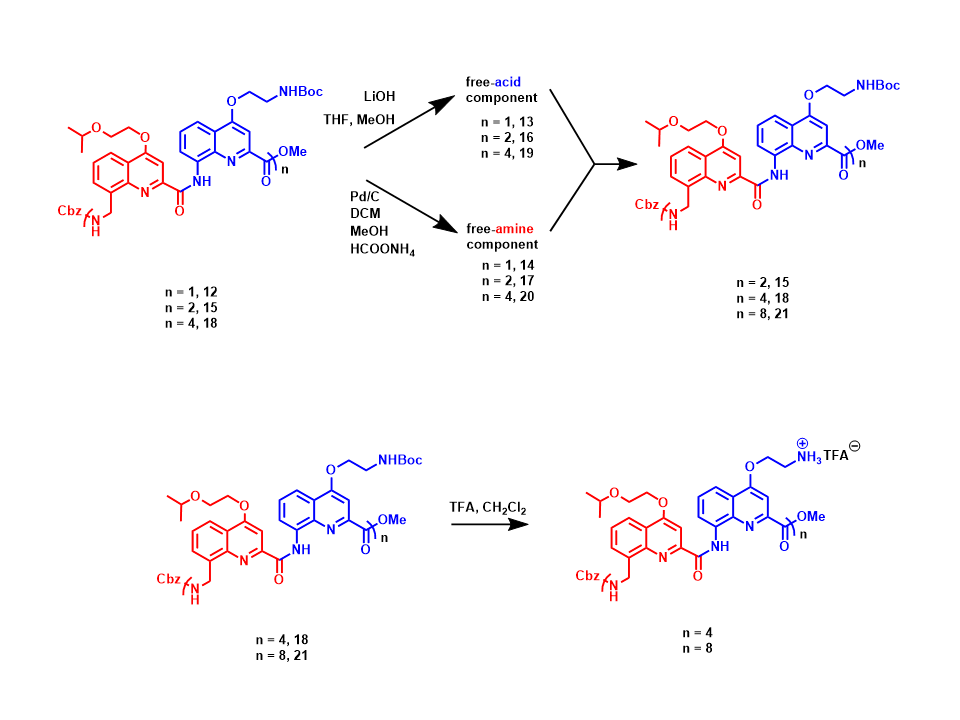


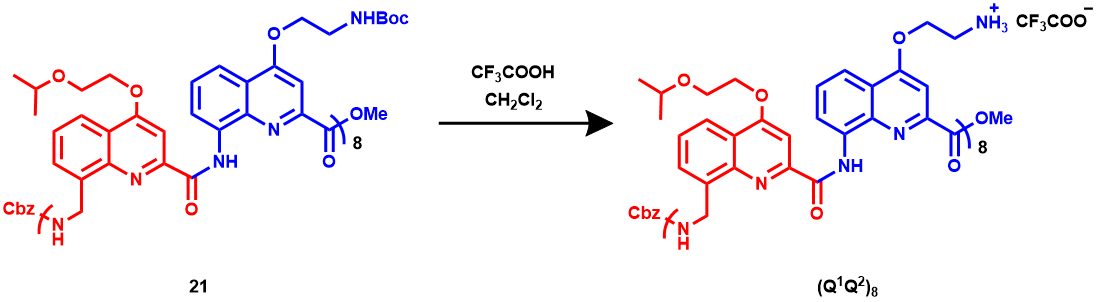


Preparation of (^m^Q^3^Q^2^)_8_.

Preparation of monomer 23.

Preparation of dimer 24.

Preparation of A^Orn^Q^3^Q^3^ sequence.

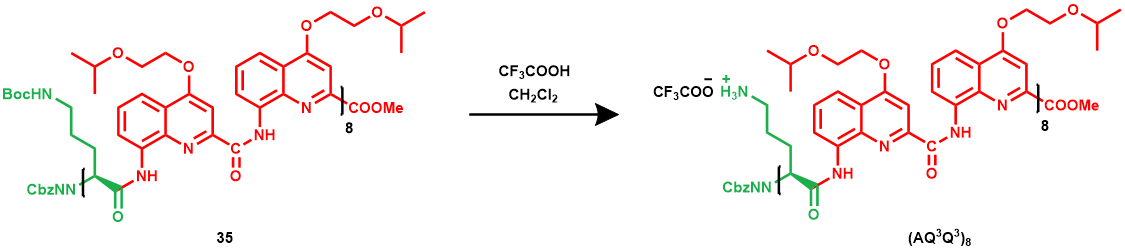


Preparation of (A^Orn^Q^3^Q^3^)_8_.

**X-Ray crystal data and molecular modeling**

The single crystals were obtained by vapor diffusion, in which Cbz-(^m^Q^3^Q^P2^)_4_-OMe and Cbz-(^m^Q^3^Q^P2^)_8_-OMe (P stands for Boc-protected side chain) were dissolved in dichloromethane while MeOH as antisolvent. The X-ray diffraction data were collected on a Bruker APEX-II CCD at Shanghai Synchrotron Radiation Facility. The molecular skeleton and side chains have been resolved but the X-ray diffraction data were not adequate to refine all hydrogen atoms. CCDC 2174578 (Cbz-(^m^Q^3^Q^P2^)_4_-OMe) and 2349304 (Cbz-(^m^Q^3^Q^P2^)_8_-OMe) contain the crystallographic data for this paper. Structures of (A^Orn^Q^3^Q^3^)_8_ and (^m^Q^3^Q^2^)_8_ were energy minimized using the Merck Molecular Force Field static (MMFF) force field implemented in MacroModel Version 8.6 via Maestro version 6.5 (Schrödinger).

Table S1. Crystallographic data for Cbz-(^m^Q^3^Q^P2^)_4_-OMe and Cbz-(^m^Q^3^Q^P2^)_8_-OMe.

| empirical formula | C_141_H_158_N_20_O_31_ | C_273_H_306_N_40_O_59_ |
| --- | --- | --- |
| formula weight | 2628.86 | 5088.20 |
| *T* [K] | 273(2) | 273(2) |
| crystal system | triclinic | triclinic |
| space group | P-1 | P-1 |
| *a* [Å] | 13.509(2) | 18.0717(12) |
| *b* [Å] | 14.937(3) | 21.7995(15) |
| *c* [Å] | 38.223(8) | 39.653(2) |
| *α* [°] | 96.595(5) | 80.1920(10) |
| *β* [°] | 97.502(5) | 85.7080(10) |
| *γ* [°] | 103.486(4) | 69.435(2) |
| *V* [Å^3^] | 7351(2) | 14410.2(16) |
| *Z* | 2 | 2 |
| F(000) | 2784 | 5430 |
| density [g/cm^3^] | 1.188 | 1.182 |
| *μ* [mm^-1^] | 0.085 | 0.084 |
| *λ/ Å* | 0.71073 | 0.71073 |
| reflections collected | 22918 | 23671 |
| Parameters/restraints | 1726/1164 | 1547/89 |

**
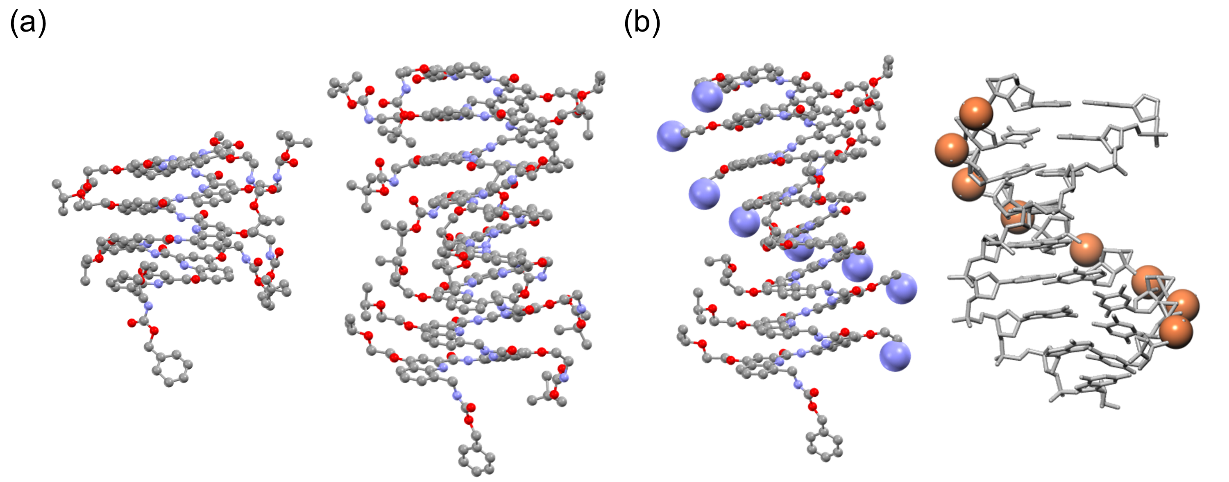
**

Figure S1. (a) The crystal structures of Cbz-(^m^Q^3^Q^P2^)_4_-OMe and Cbz-(^m^Q^3^Q^P2^)_8_-OMe_._ (b) The crystal structure of Cbz-(^m^Q^3^Q^P2^)_8_-OMe with the Boc hidden and the model structure of ds-DNA. Their structures are shown at the same scale as the stick representations except that the phosphorus atoms are shown as orange spheres and the nitrogen atoms are shown as blue spheres.

**Fluorescence titration spectrum**

Fluorescence titration experiments of foldamers with ss-DNA were carried out with a SHIMADZU RF-5301PC Spectro fluorophotometer at excitation 360 nm. The foldamers with increments of ss-DNA (dT_9_ (5’-TTT TTT TTT- 3’), dA_9_ (5’-AAA AAA AAA- 3’), dG_9_ (5’-GGG GGG GGG- 3’) and dC_9_ (5’-CCC CCC CCC- 3’)) were dissolved in 10 mM Tris·HCl, 10 mM NaCl, pH = 7.4. The relative FL intensity were calculated according to the equation:

I_intensity change_ = (F- F**_0_**)/F**_0_**

Where F**_0_** is the fluorescence intensity of foldamer, F is the fluorescence intensity when ss-DNA was added accordingly.


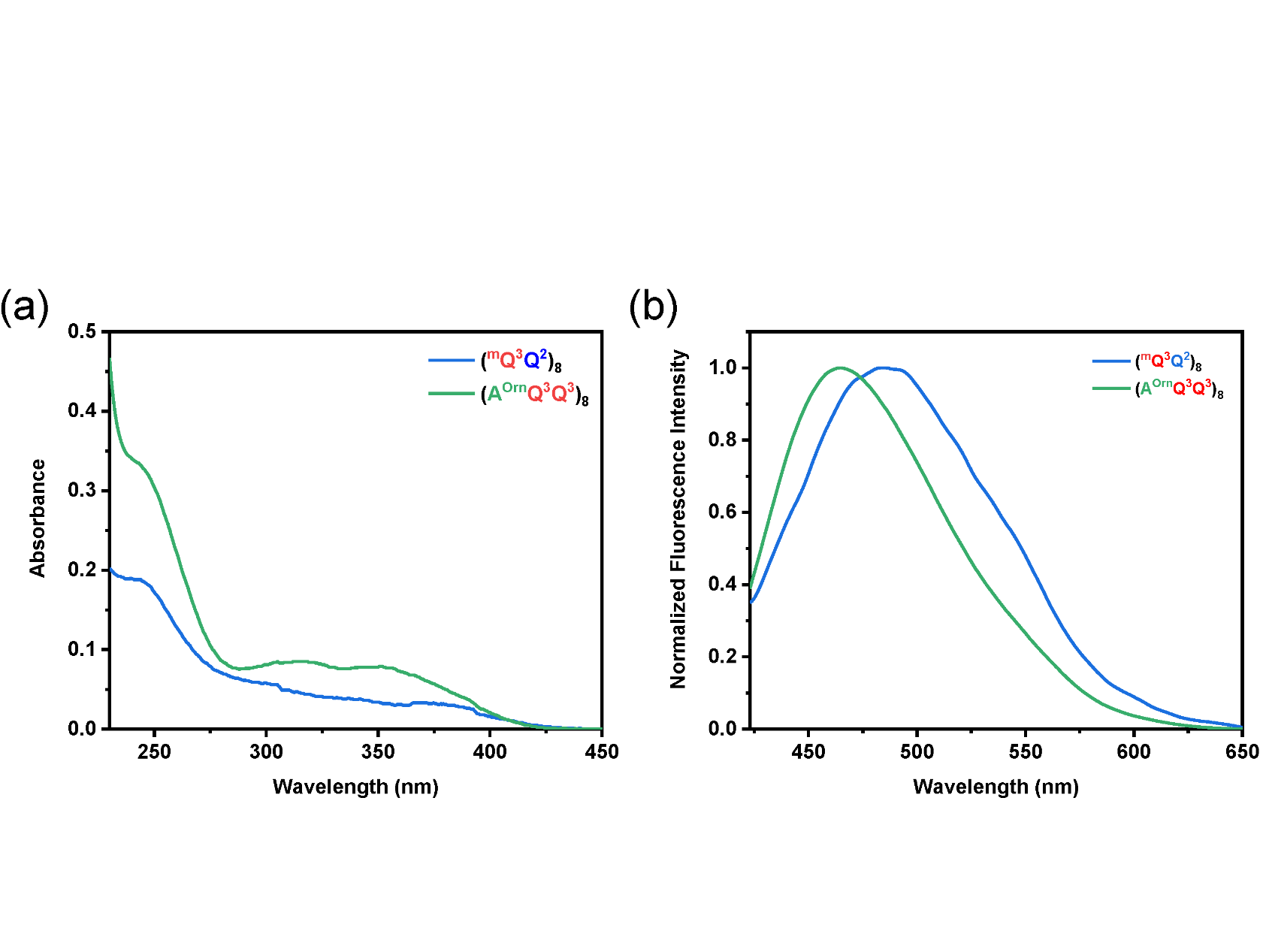


Figure S2. (a) UV spectra of 2.0 μM foldamers in 10 mM Tris·HCl, 10 mM NaCl, pH = 7.4. (b) The normalized emission of 2.0 μM foldamers in 10 mM Tris·HCl, 10 mM NaCl, pH = 7.4.


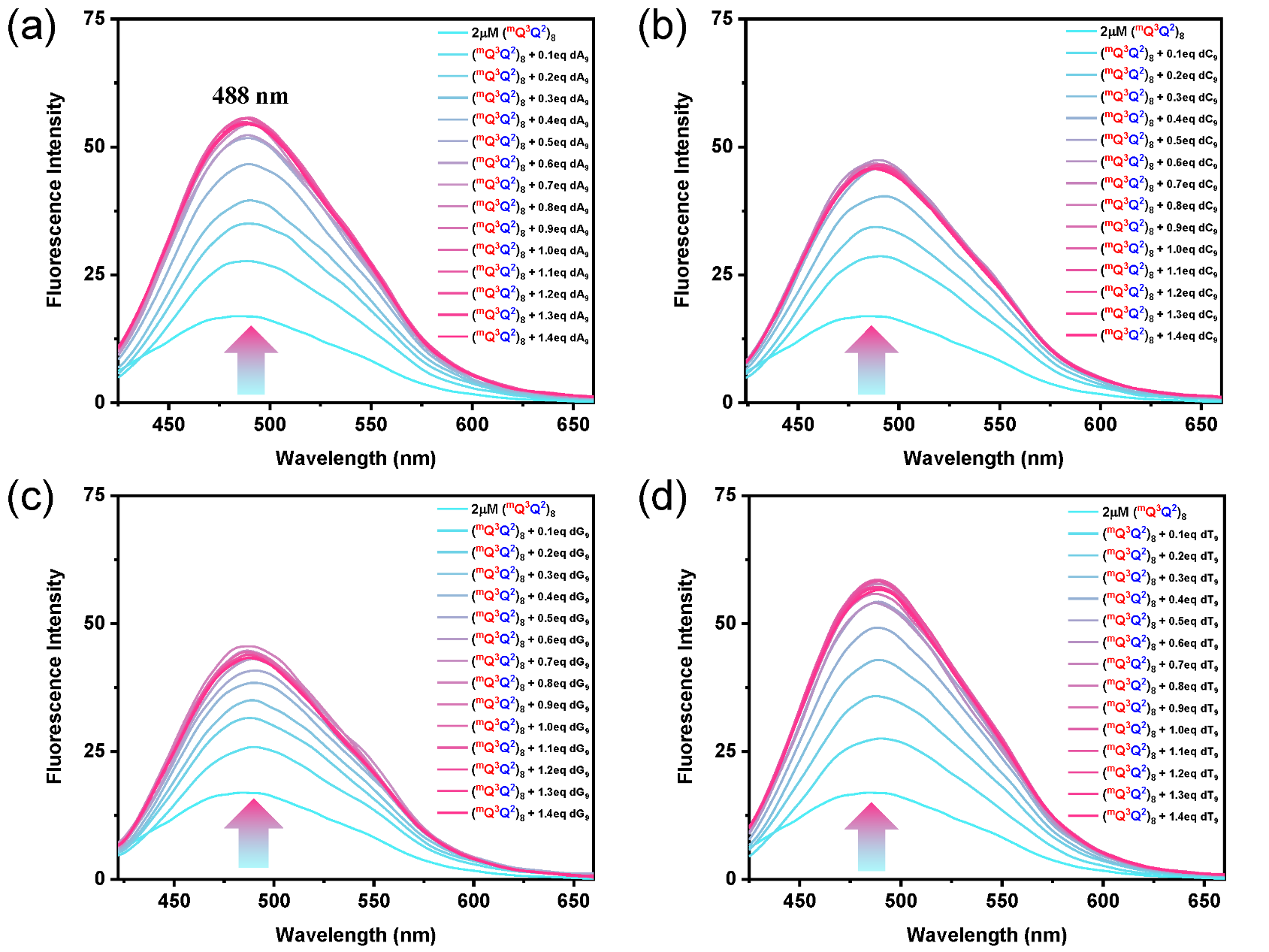


Figure S3. The fluorescence titrations of (^m^Q^3^Q^2^)_8_ at the concentration of 2 μM in 10 mM Tris·HCl, 10 mM NaCl, pH = 7.4 upon titrating 0-1.4 equivalent amounts of (a) dA_9_, (b) dC_9_, (c) dG_9_, and (d) dT_9_, respectively.


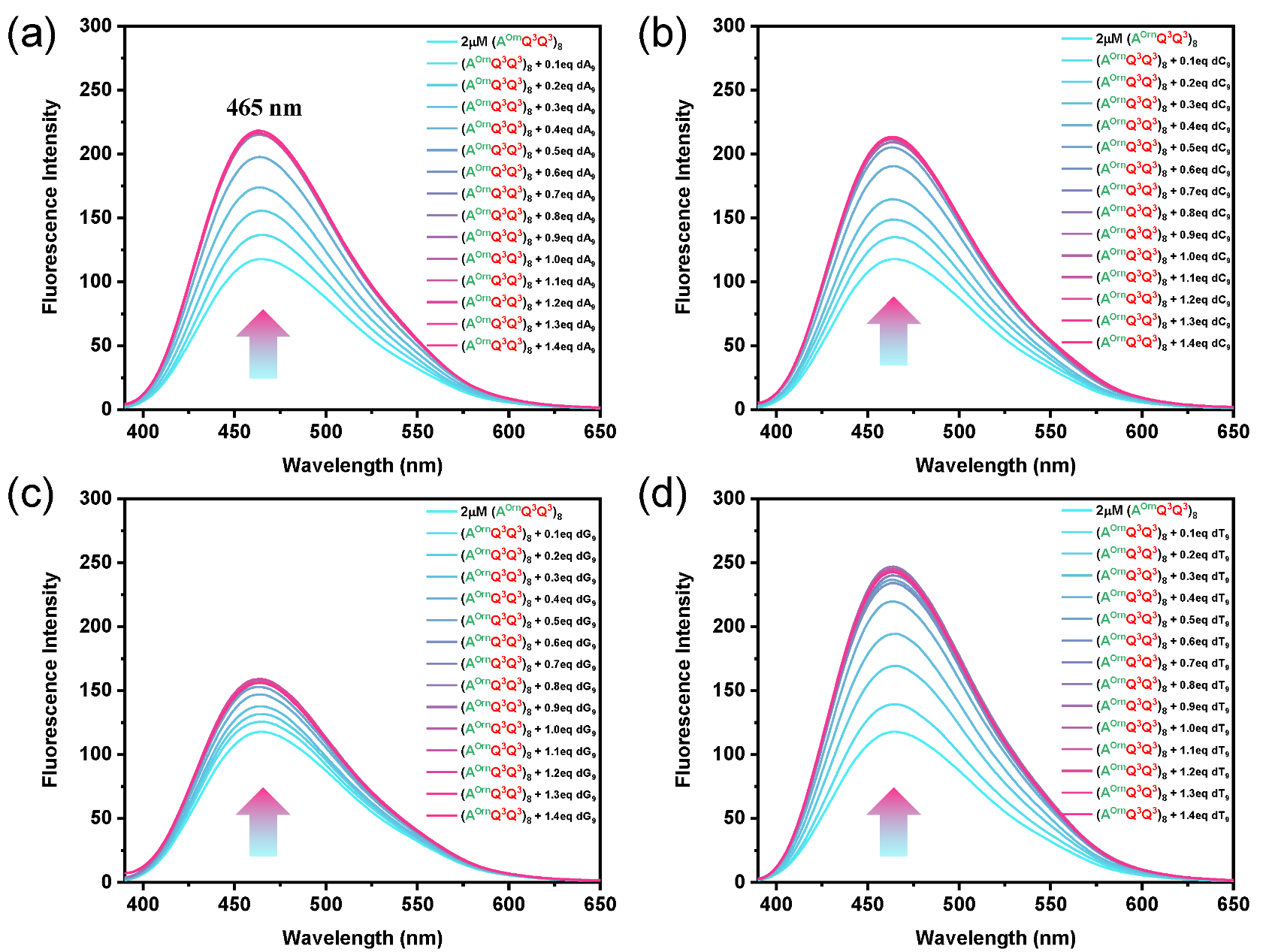


Figure S4. The fluorescence titrations of (A^Orn^Q^3^Q^3^)_8_ at the concentration of 2 μM in 10 mM Tris·HCl, 10 mM NaCl, pH = 7.4 upon titrating 0-1.4 equivalent amounts of (a) dA_9_, (b) dC_9_, (c) dG_9_, and (d) dT_9_, respectively.

Fluorescence titration experiments of dT_9_-FAM with foldamers were carried out with a SHIMADZU RF-5301PC Spectro fluorophotometer at excitation 480 nm. The dT_9_-FAM with increments of foldamers were dissolved in 10 mM Tris·HCl, 10 mM NaCl, pH = 7.4. The relative FL intensity were calculated according to the equation^[1]^:

$$\mathbf{log}\left[ \frac{\left( \boldsymbol{F}_{\boldsymbol{0}}\mathbf{-}\boldsymbol{F} \right)}{\boldsymbol{F}} \right]\mathbf{=log}\boldsymbol{K}_{\boldsymbol{a}}\boldsymbol{+}\mathbf{nlog}\left[ \boldsymbol{(Foldamer} \right)\boldsymbol{]}$$

Where F**_0_** is the initial fluorescence intensity of dT_9_-FAM (2.5 μM), F is the fluorescence intensity when foldamer ((^m^Q^3^Q^2^)_8_ or (A^Orn^Q^3^Q^3^)_8_) was added accordingly. Binding constant K_a_ of (^m^Q^3^Q^2^)_8_ and (A^Orn^Q^3^Q^3^)_8_ were determined as 1.67 ×10^6^ M^-1^ and 4.25 ×10^6^ M^-1^ and n of (^m^Q^3^Q^2^)_8_ and (A^Orn^Q^3^Q^3^)_8_ were determined as 1.1 and 1.2, respectively.


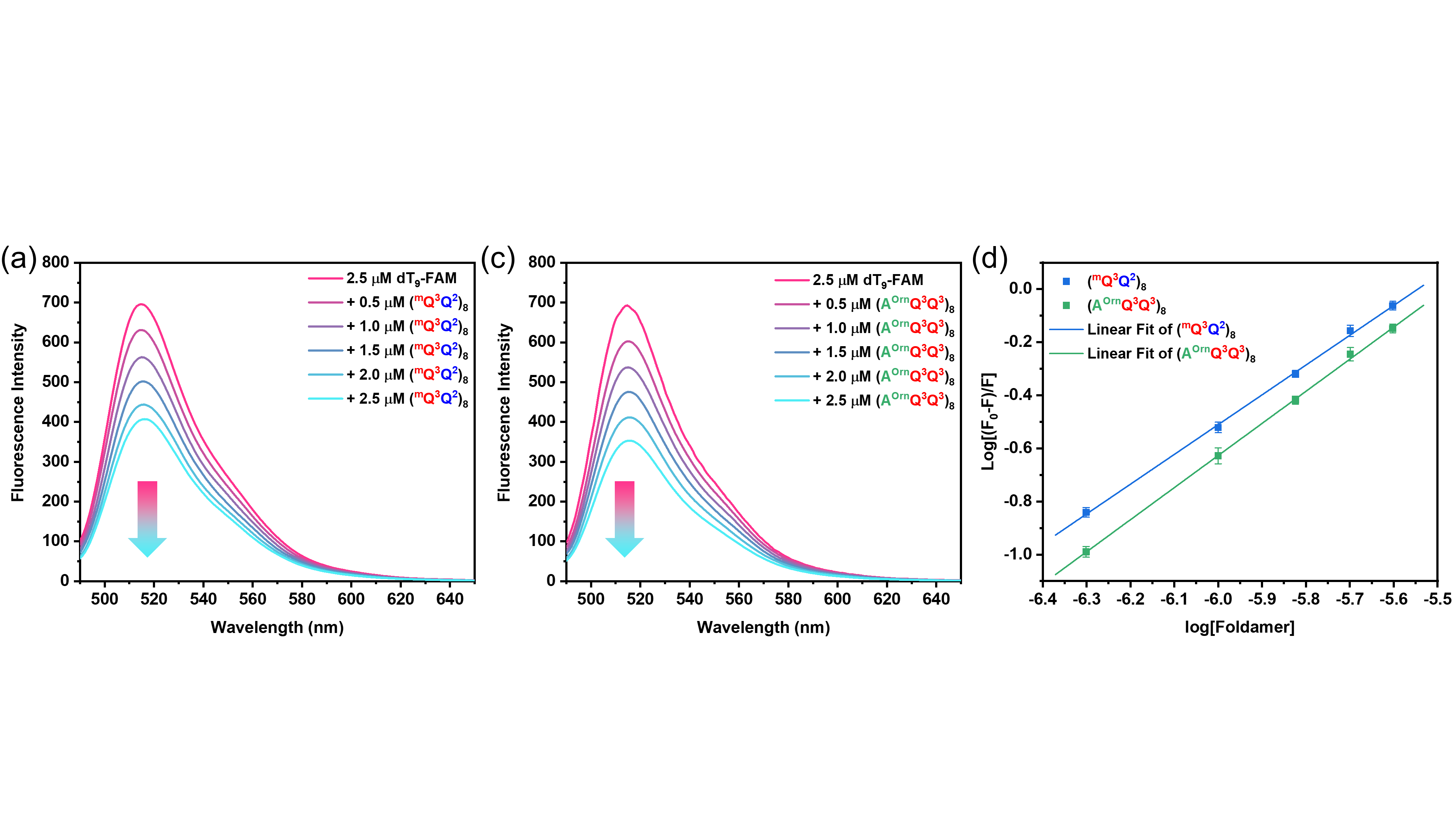


Figure S5. The fluorescence spectra of dT_9_-FAM at the concentration of 2.5 μM upon titrating 0-2.5 μM (a) (^m^Q^3^Q^2^)_8_ and (b) (A^Orn^Q^3^Q^3^)_8_. (c) Plots of log((F_0_-F)/F) vs log [Foldamer].

**Zeta potential Experiments**

10 mM Tris·HCl, 10 mM NaCl, pH=7.4 was used as buffer solution. (^m^Q^3^Q^2^)_8_ and (A^Orn^Q^3^Q^3^)_8_ were brought to a final concentration of 1.0 μM or 2.5 μM. The final potential was obtained from the average of three tests by Litesizer 500.


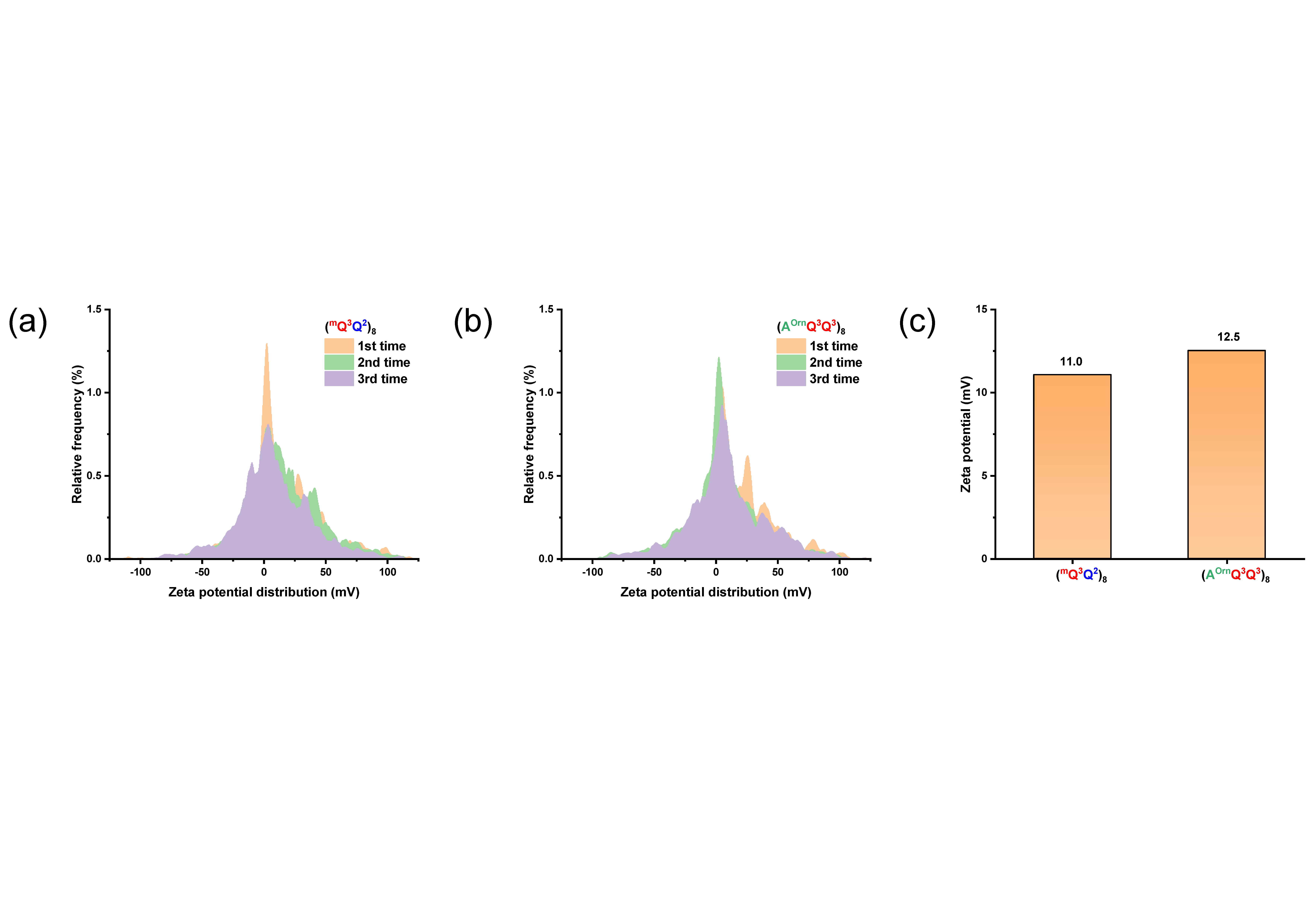


Figure S6. Zeta potential distribution graphs of (a) 1.0 μM (^m^Q^3^Q^2^)_8_, (b) 1.0 μM (A^Orn^Q^3^Q^3^)_8_, and (c) the average potential of (^m^Q^3^Q^2^)_8_ and (A^Orn^Q^3^Q^3^)_8_ in 10 mM Tris·HCl, 10 mM NaCl, pH = 7.4.


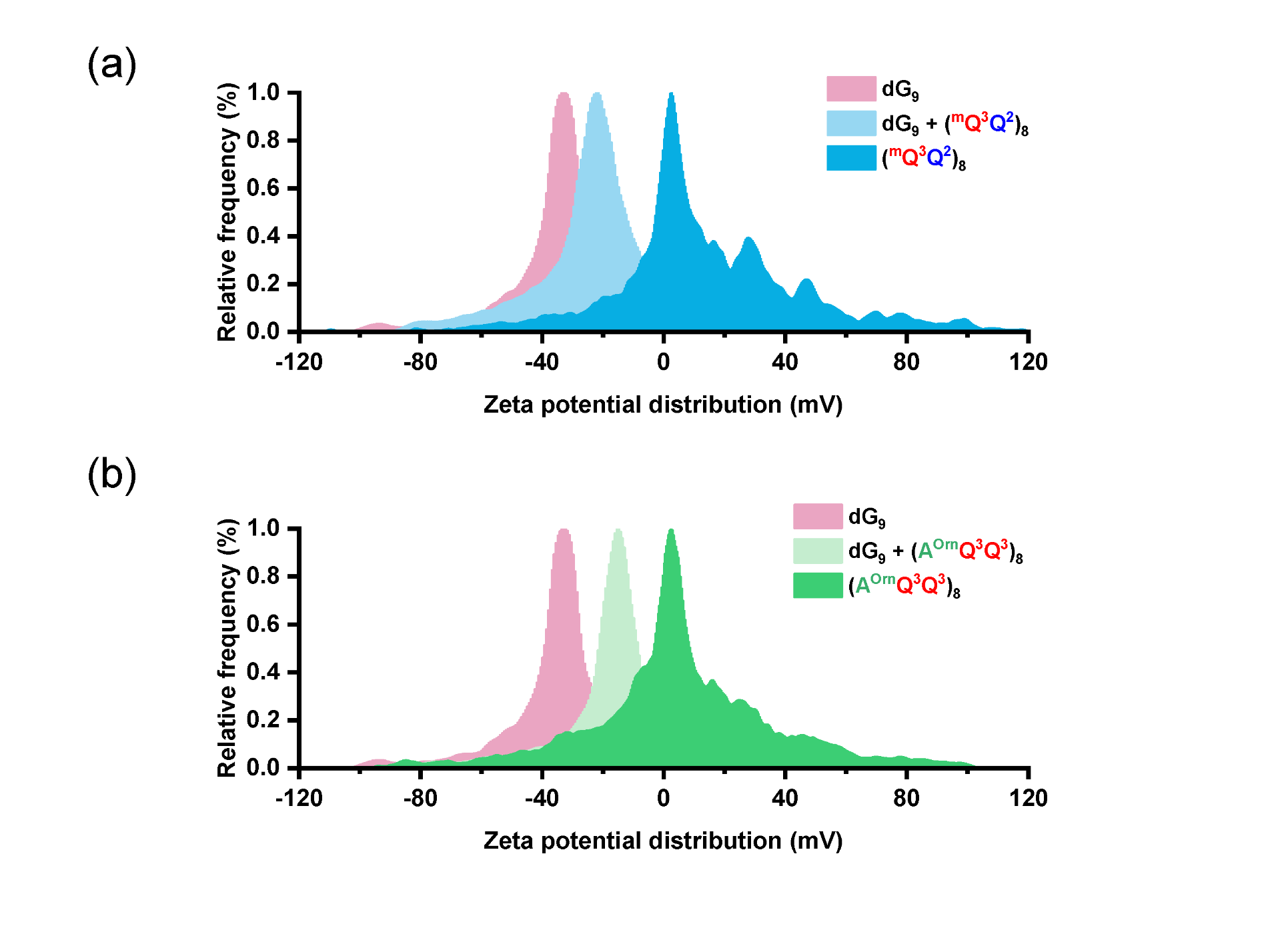


Figure S7. Zeta potential distribution graphs of (a) 2.5 μM (^m^Q^3^Q^2^)_8_ and (b) 2.5 μM (A^Orn^Q^3^Q^3^)_8_ with 2.5 μM dG_9_ in 10 mM Tris·HCl, 10 mM NaCl, pH = 7.4.

**CD experiments**

ss-DNA dT_13_ (5’-TTT TTT TTT TTT T-3’) and ds-DNA (5’-CCA GTA CTG G-3’) was suspended at 10 μM in 10 mM Tris·HCl, pH = 7.4 and annealed by heating to 90 °C for 5 min, cooled to room temperature slowly. Spectra were measured every 0.5 nm between 480 nm and 220 nm with 1 s averaging time. Samples were incubated at room temperature for 20 minutes prior to scan.


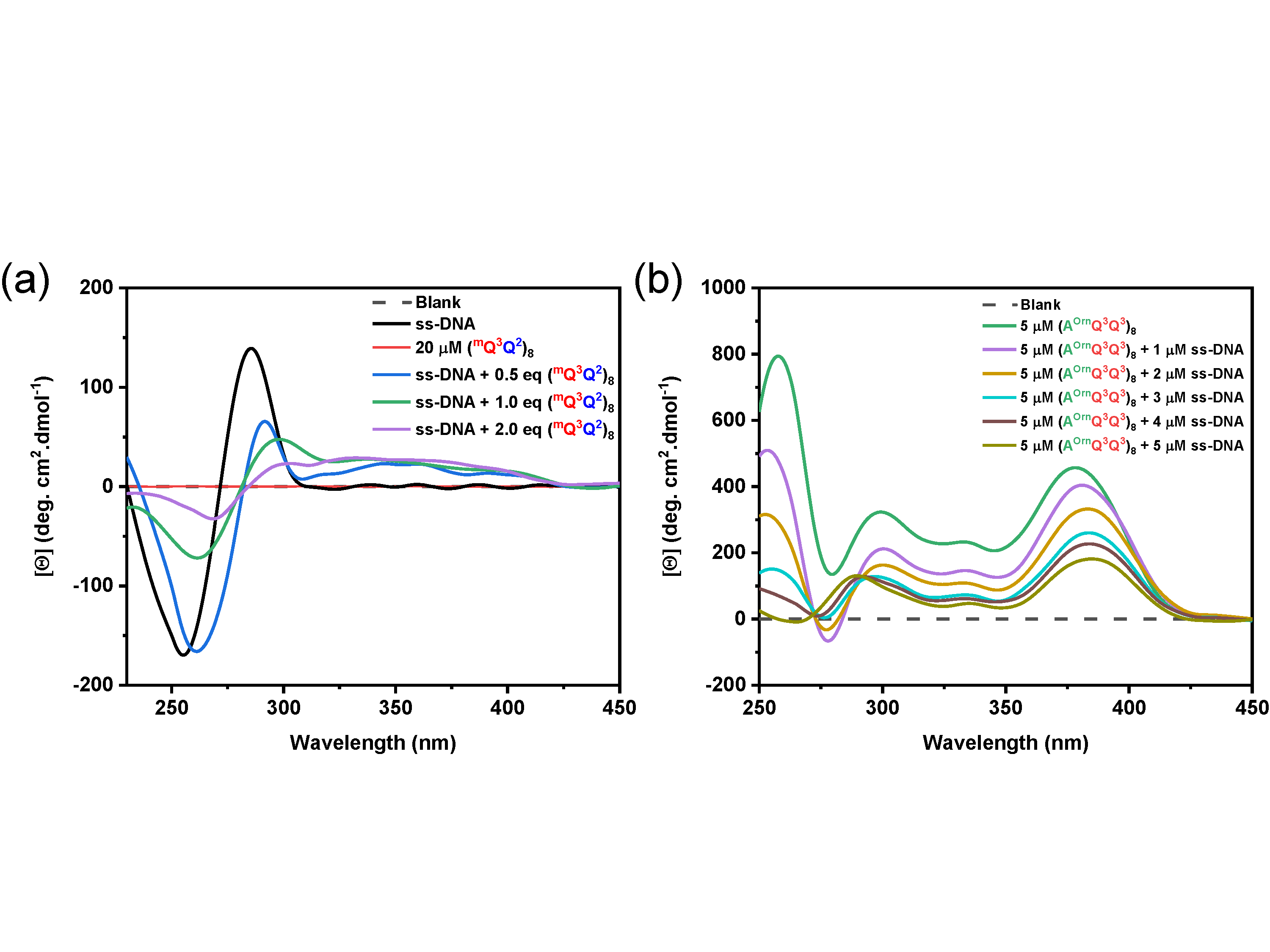


Figure S8. (a) The CD spectra of dT_13_ at the concentration of 10 μM upon titrating of (^m^Q^3^Q^2^)_8_. (b) The CD spectra of (A^Orn^Q^3^Q^3^)_8_ at the concentration of 5 μM upon titrating of dT_13_.


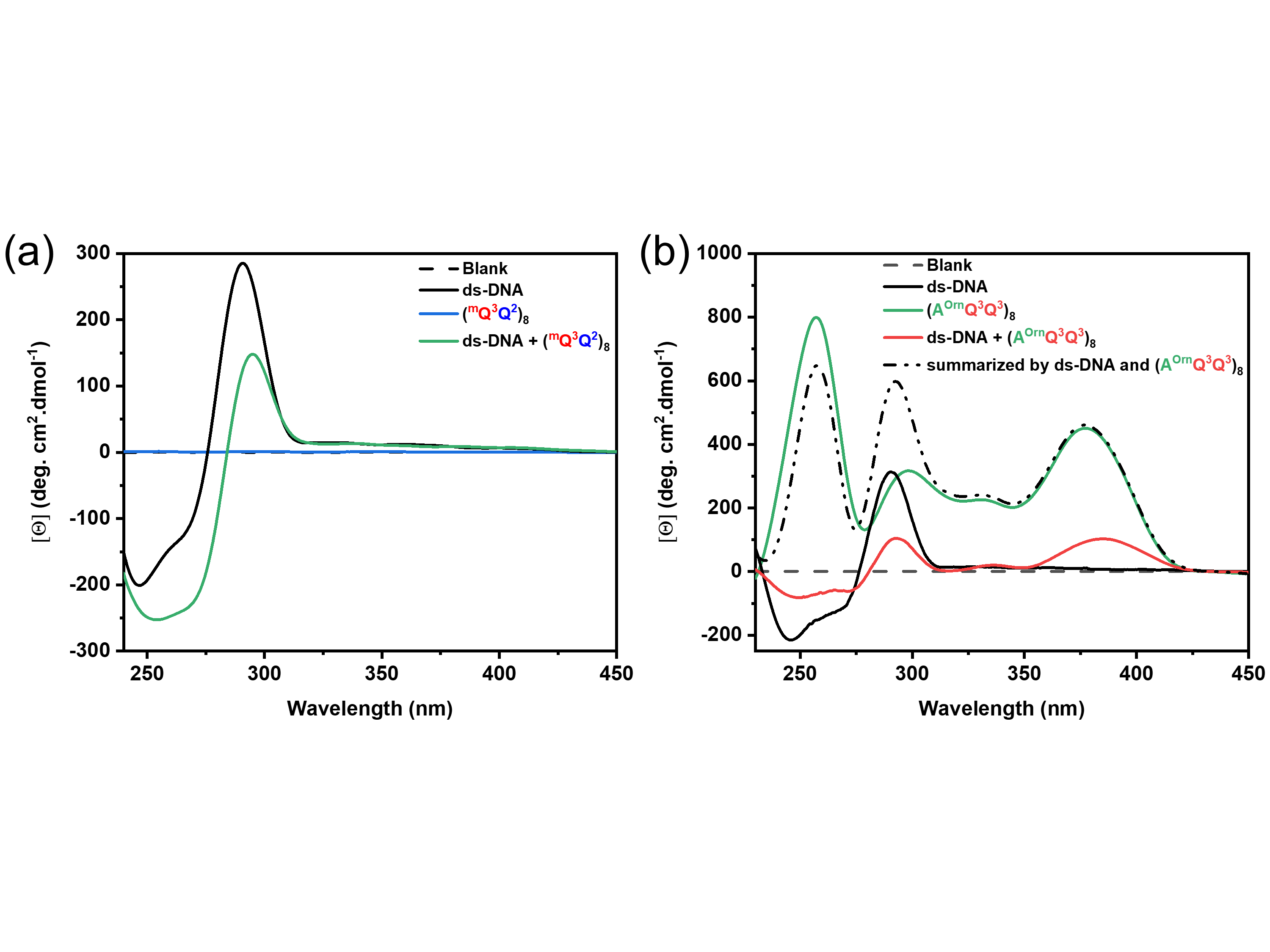


Figure S9. CD spectra of 10 μM ds-DNA upon addition of 5 μM (a) (^m^Q^3^Q^2^)_8_ and (b) (A^Orn^Q^3^Q^3^)_8_ in 10 mM Tris·HCl, 10 mM NaCl, pH = 7.4.

**Atomic Force Microscope images**

Atomic Force Microscope (AFM) images were obtained with German Bruker Dimension 3100 instrument in tapping mode. The compounds were dissolved in 10 mM Tris·HCl, 10 mM NaCl, pH = 7.4. Sample solution (1.5 μL) was deposited on the fresh silicon wafer, and then dried at room temperature.

**
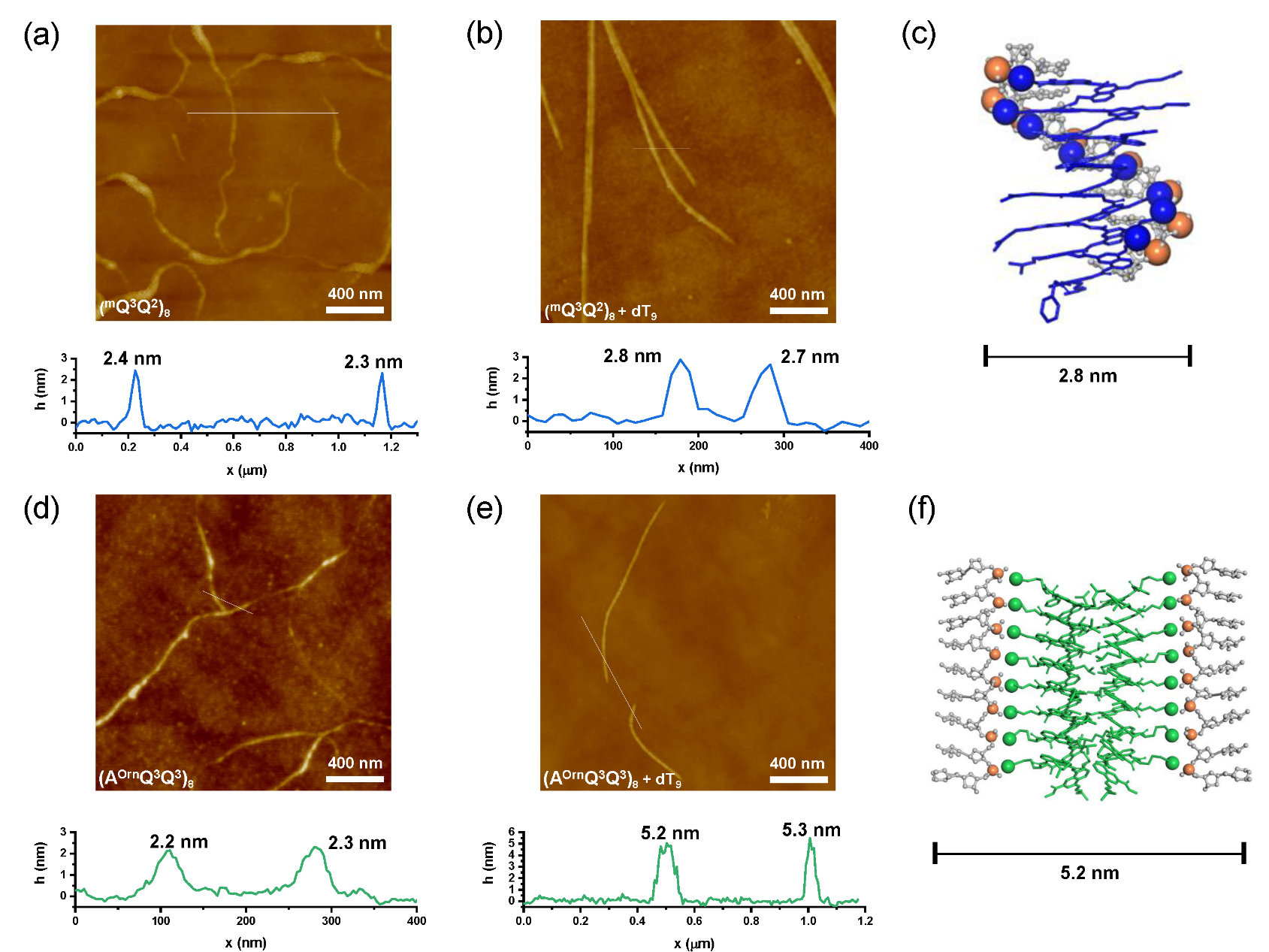
**

Figure S10. AFM images of (a) 10 μM (^m^Q^3^Q^2^)_8_ and (b) 10 μM (^m^Q^3^Q^2^)_8_ with 2 μM dT_9_; (c) Proposed interaction model of ss-DNA and (^m^Q^3^Q^2^)_8_; AFM images of (d) 10 μM (A^Orn^Q^3^Q^3^)_8_ and (e) 10 μM (A^Orn^Q^3^Q^3^)_8_ with 2 μM dT_9_; (f) Proposed interaction model of ss-DNA and (A^Orn^Q^3^Q^3^)_8_. The structure models (c) and (f) of DNA bound foldamers are speculation on the structural arrangement based on the morphologies observed by the AFM experiments.


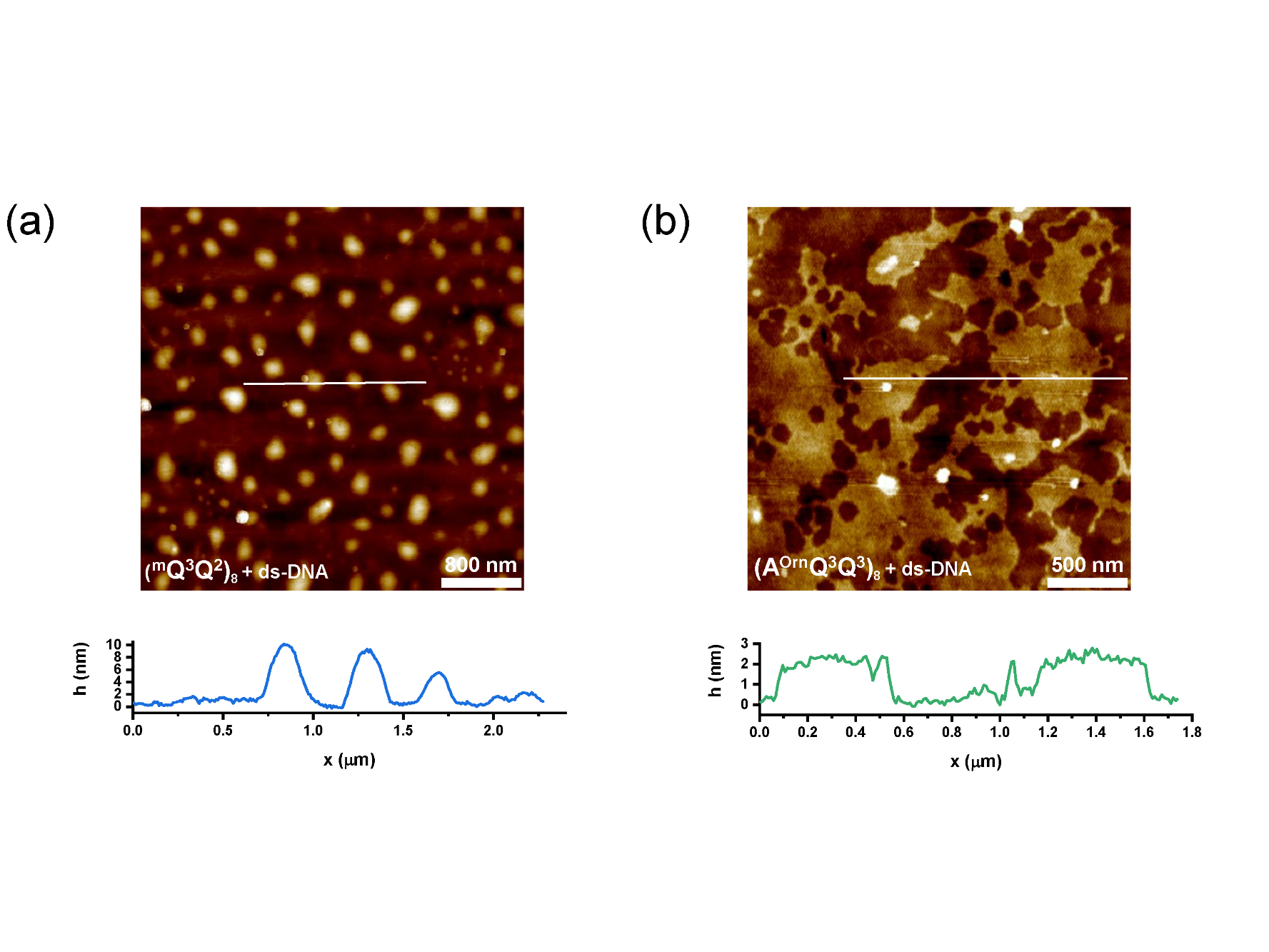


Figure S11. AFM images of (a) 10 μM (^m^Q^3^Q^2^)_8_ and (b) 10 μM (A^Orn^Q^3^Q^3^)_8_ with 2 μM ds-DNA.

**UV Melting Analysis**

An aqueous solution of 10 mM sodium cacodylate (CacoNa), 10 mM NaCl, pH=7.4 was used as analysis buffer and 15bp duplex DNA (5’-AGC CTA GGA TAA GAG-3’) was brought to a final concentration of 2.5 μM. Samples were heated to 90°C for 5 min, cooled to room temperature slowly. Foldamers (1.25 μM) or branched polyethyleneimine (branched-PEI, M.W. 600) and ds-DNA (2.5 μM) were incubated for 0.5 h at room temperature. Denaturation was recorded at 260 nm from 10°C to 80°C with a heating rate of 0.5°C/min.

Thermal denaturation profiles were obtained with LAMBDA 1050+ UV Spectrophotometer.


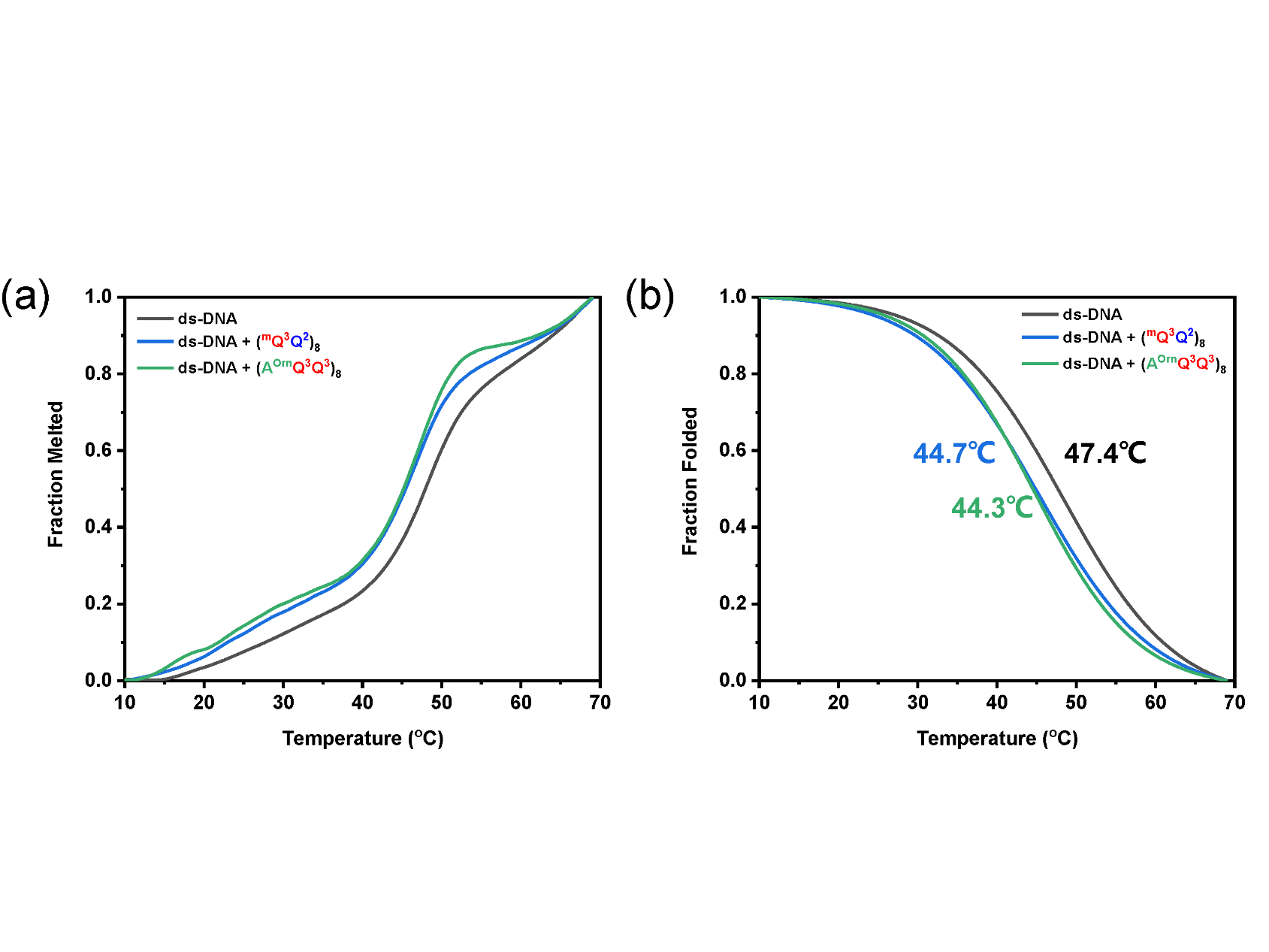


Figure S12**.** (a) Normalized plots from UV thermal melting experiment with ds-DNA at 2.5 uM in the absence (black) and presence of 0.5 equiv. (^m^Q^3^Q^2^)_8_ (blue) and 0.5 equiv. (A^Orn^Q^3^Q^3^)_8_ (green). (b) Fraction folded plots for ds-DNA at 2.5 μM in the absence (black) and presence of 0.5 equiv. (^m^Q^3^Q^2^)_8_ (blue) and 0.5 equiv. (A^Orn^Q^3^Q^3^)_8_ (green).


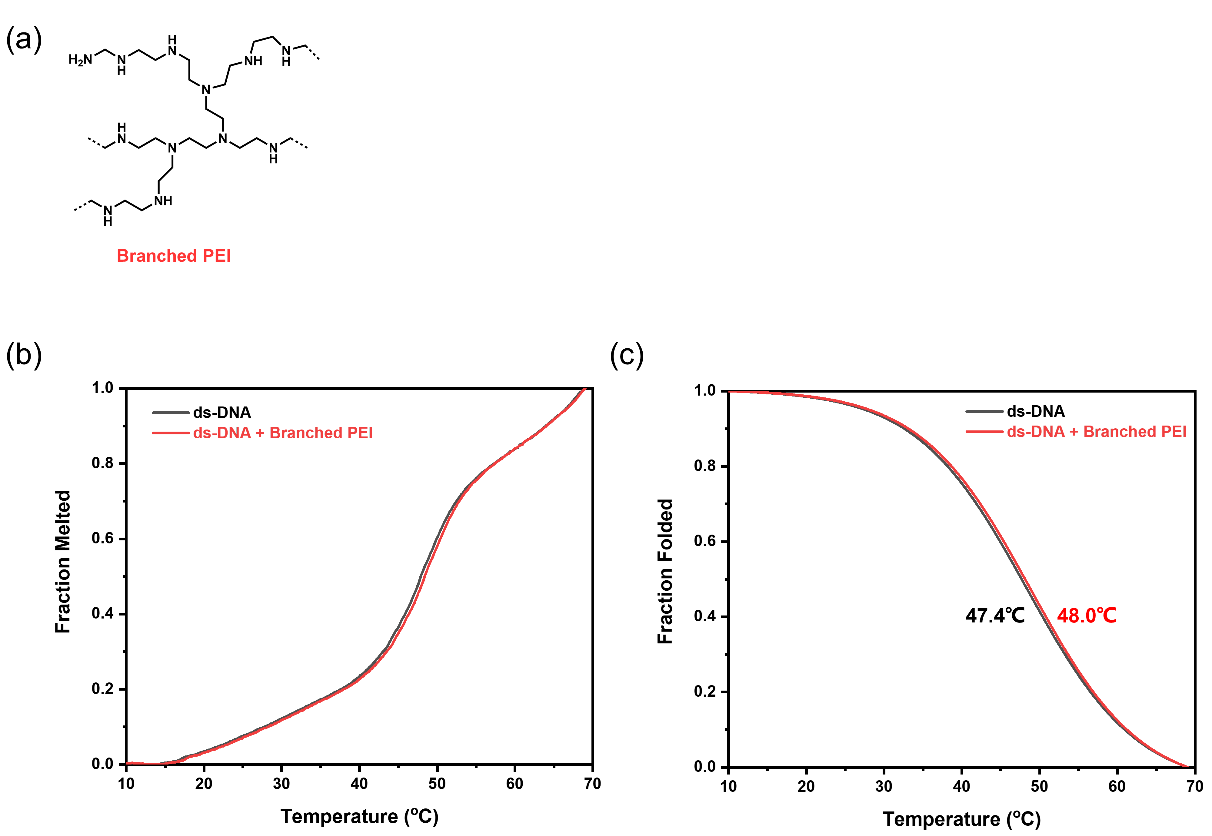


Figure S13. UV thermal stabilization data for (a) branched-PEI in 10 mM CacoNa, 100 mM NaCl, pH = 7.2. (b) Normalized plots from UV thermal melting experiment with ds-DNA at 2.5 μM in the absence (black) and presence of 0.5 equiv. branched-PEI (red). (c) Fraction folded plots for ds-DNA at 2.5 μM in the absence (black) and presence of 0.5 equiv branched branched-PEI (red).

**Fluorescence tests on recognition of single- and double-stranded oligonucleotides experiments**

Fluorescence experiments of foldamers with ss-DNA dT_9_ and ds-DNA (5’-FAM-CCA GTA CTG G-TAMRA-3’) were carried out with FLS980 Spectrometer at the excitation of 480 nm. The fluorescence intensity was monitored by sequential dropwise addition of (^m^Q^3^Q^2^)_8_ or (A^Orn^Q^3^Q^3^)_8_ to double labeled ds-DNA (1.0 μM) in 10 mM Tris·HCl, 10 mM NaCl, pH = 7.4. The fluorescence intensity was monitored by sequential dropwise addition of (^m^Q^3^Q^2^)_8_ or (A^Orn^Q^3^Q^3^)_8_ to mixed solution of dT_9_ (2.25 μM) and double labeled ds-DNA (1.0 μM) in 10 mM Tris·HCl, 10 mM NaCl, pH = 7.4_._ The titration details were calculated according to the equation:

I_intensity change_ = F_M_/F_0_

Where F_0_ is the fluorescence intensity without foldamer, F_M_ is the fluorescence intensity when foldamer was added accordingly.


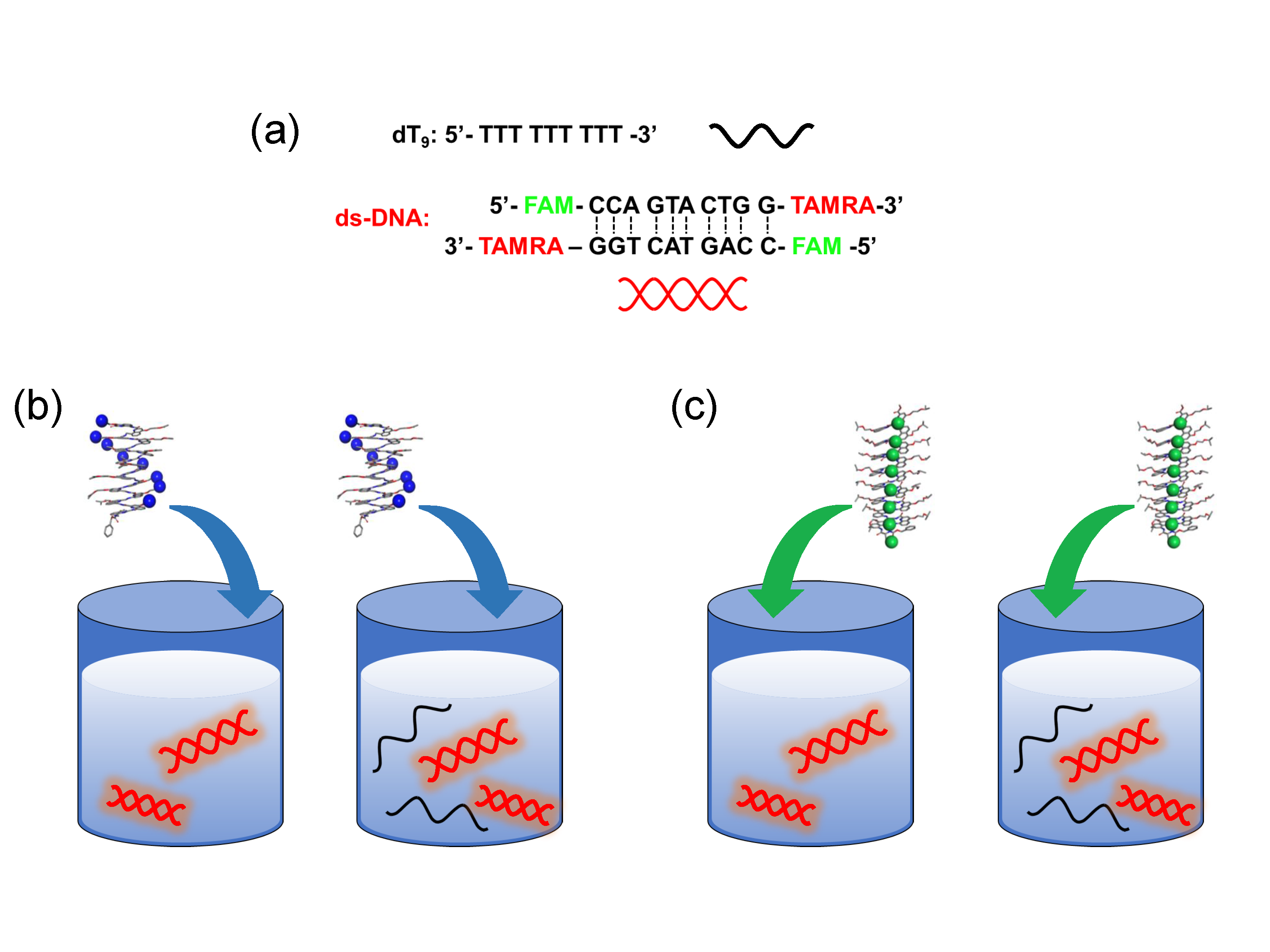


Figure S14. (a) The sequence of ss-DNA and ds-DNA were used in selective recognition. (b) The fluorescence signal of TAMRA was detected upon titrating (^m^Q^3^Q^2^)_8_ into different cells at 480 nm excitation. (c) The fluorescence signal of TAMRA was detected upon titrating (A^Orn^Q^3^Q^3^)_8_ into different cells at 480 nm excitation.

**Vesicle-based kinetic experiments**

The typical procedure of preparation of rhodamine B entrapped in large lamellar vesicles (LUVs) was as follow: 10.0 mg of egg yolk L-α-phosphatidylcholine (EYPC) was dissolved in dry CDCl_3_ (2 mL) and the solution was dried with N_2_ flow. The thin film of EYPC was dried under vacuum for 3 hours to remove the solvent completely, then the lipid was hydrated with 1 mL of buffer solution (Tris, 10 mM, pH = 7.4) containing 100 mM of NaCl and 1 mM of dye rhodamine B for 3 hours at 37 ℃. The suspension was carried out 10 times freeze-thaw cycles with liquid nitrogen and thermostat water bath and was extruded for 10 times using 200 μm polycarbonate membrane, and then was purified by Sephadex G-50 to remove the dye outside the vesicles (mobile phase: Tris buffer (10 mM, pH = 7.4) with 100 mM NaCl). To 400 μL 100 mM NaCl buffer solution (Tris, 10 mM, pH = 7.4) in a quartz fluorimetric cell was added 50 μL of LUVs containing rhodamine B. The emission of rhodamine B at 570 nm was monitored at excitation 480 nm, and then 10 μL of mixed solution of dT_9_-FAM (1.0 μM) and foldamers of different concentration ratios or controls (linear polyethyleneimine (average Mn 5000, PDI < 1.2), branched polyethyleneimine (W.M. 600)) in Tris was added. Finally, the monitoring was stopped by lysing the vesicles with detergent (10 μL of 200 μM aqueous melittin). The injection part of the spectrum was subtracted for clarity. The collected time course data Et was normalized according to the equation:

R_f_ = E_t_/E_0_

Where E_0_ is the initial emission intensity.

The typical procedure of preparation of carboxyfluorescenin (CF) entrapped in large lamellar vesicles (LUVs) was as same as rhodamine B. The emission of CF at 520 nm was monitored at excitation 480 nm, and then 10 μL of dT_9_ (1.0 μM) or the mixed solution of dT_9_ (1.0 μM) with foldamer (1.0 μM) in Tris was added. Finally, the monitoring was stopped by lysing the vesicles with detergent (10 μL of 200 μM aqueous melittin). The injection part of the spectrum was subtracted for clarity. The collected time course data Et was normalized according to the equation:

R_f_ = E_t_/E_0_

Where E_0_ is the initial emission intensity.


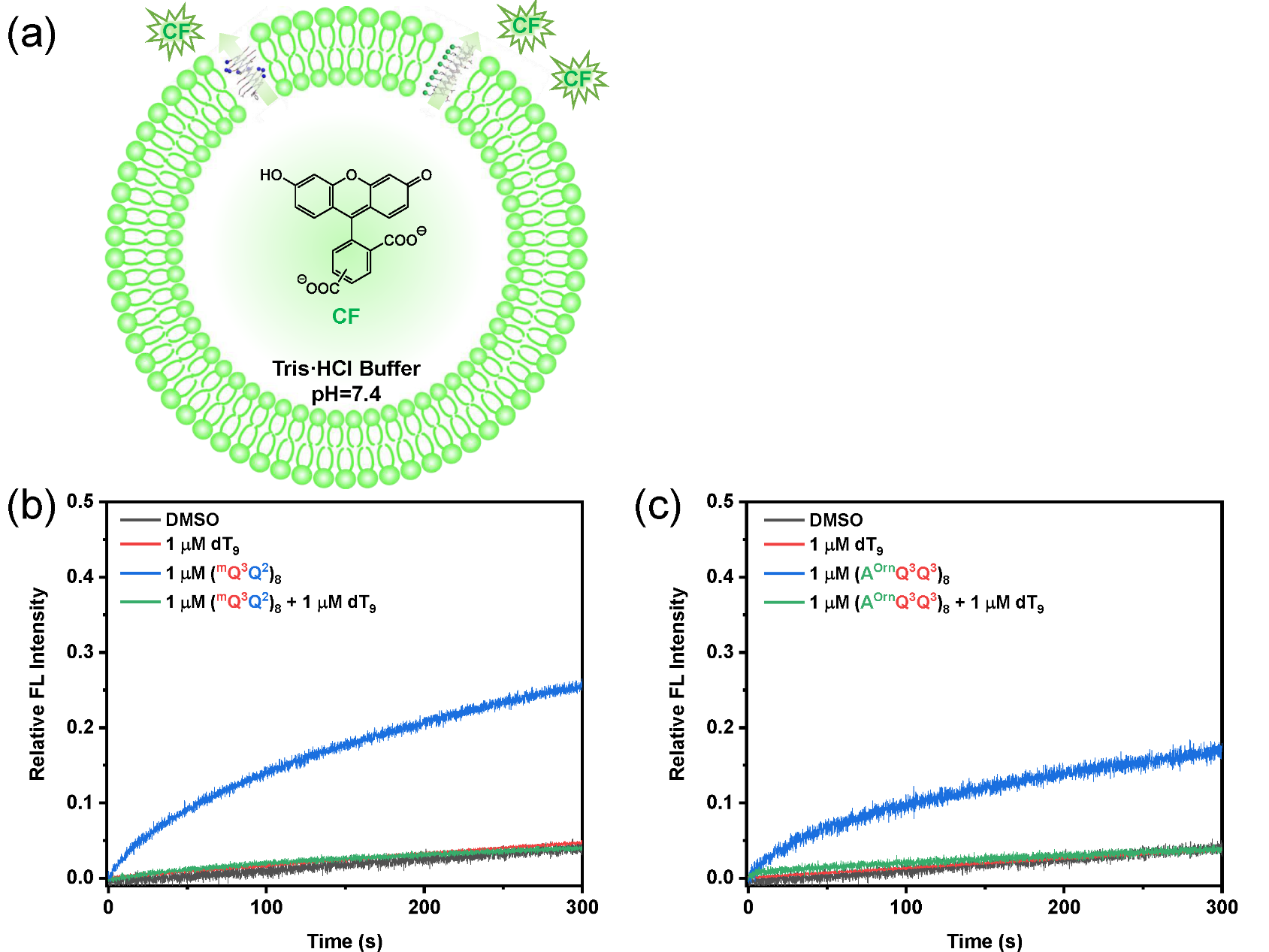


Figure S15. (a) Schematic of membrane rupture experiments by foldamers. (b) Normalized capacity of membrane rupture by (^m^Q^3^Q^2^)_8_. (c) Normalized capacity of membrane rupture by (A^Orn^Q^3^Q^3^)_8_.


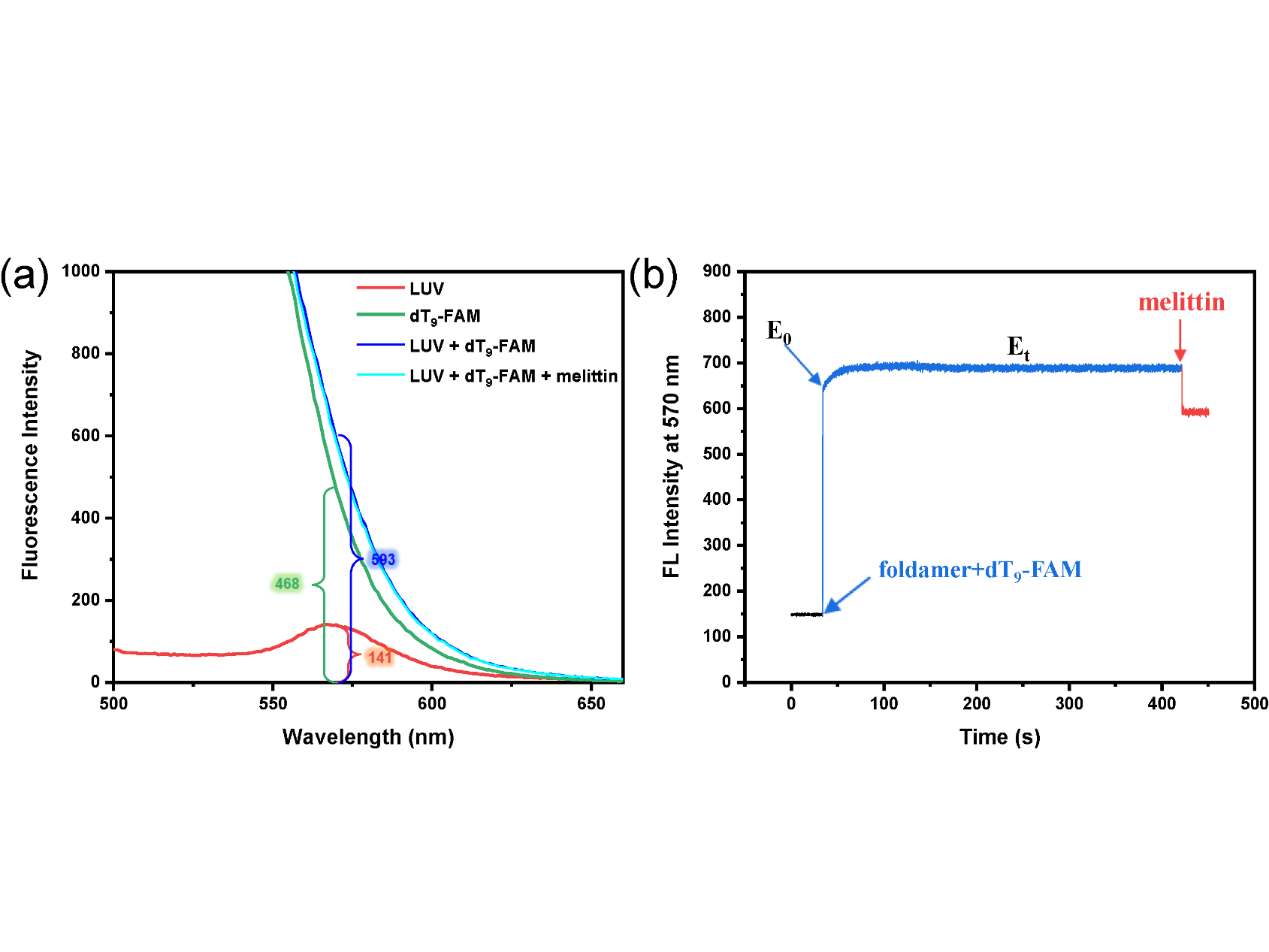


Figure S16. (a)The fluorescence spectra of LUVs containing rhodamine B with dT_9_-FAM (5’- FAM-TTT TTT TTT-3’), suggesting that there is no FRET under the condition. (b) The example of dT_9_- FAM transport experiment by (^m^Q^3^Q^2^)_8_.


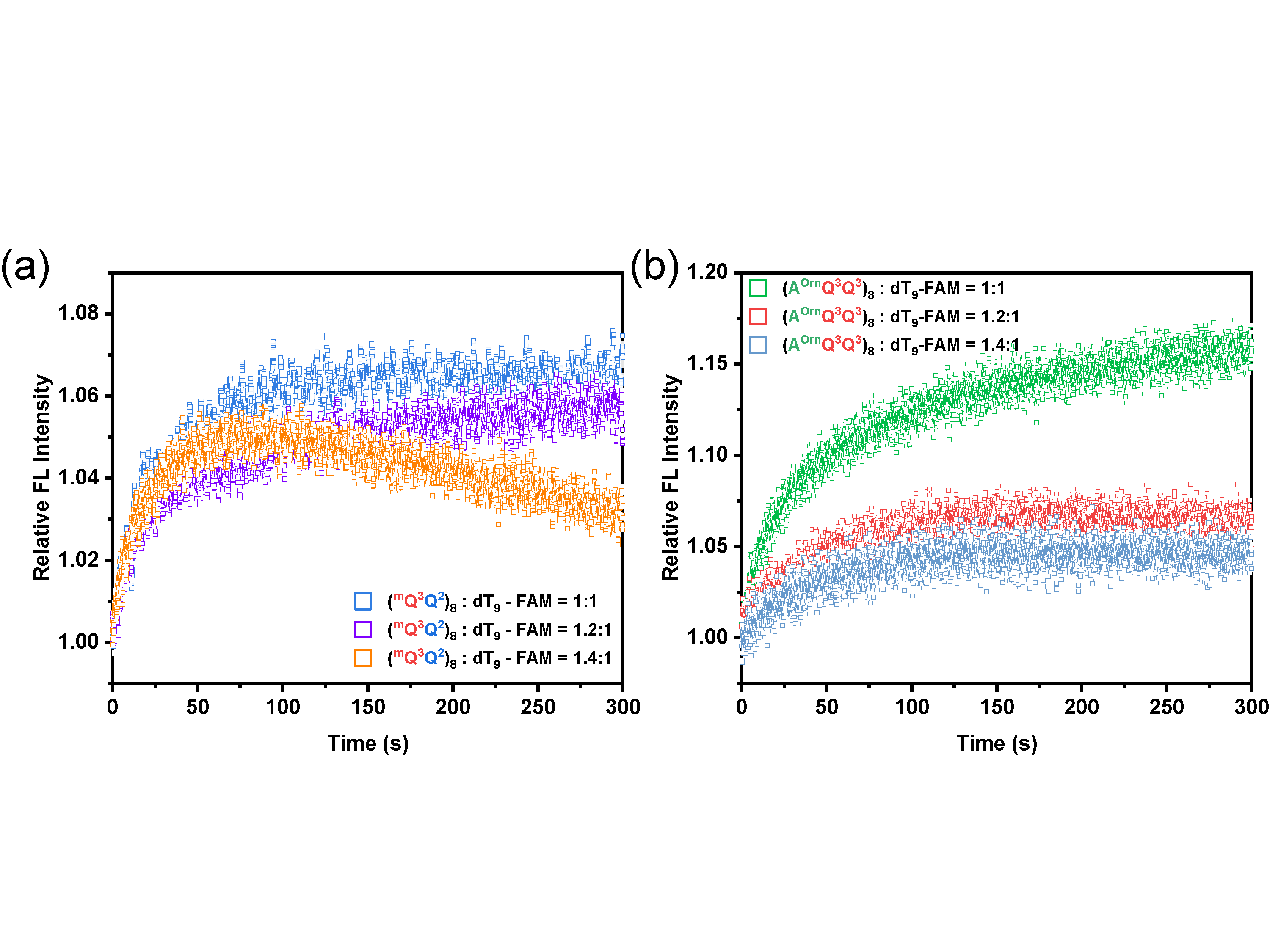


Figure S17. Normalized dT_9_-FAM (5’- FAM-TTT TTT TTT-3’) transport activities of (a) (^m^Q^3^Q^2^)_8_ and (b) (A^Orn^Q^3^Q^3^)_8_ for at different concentration ratios.


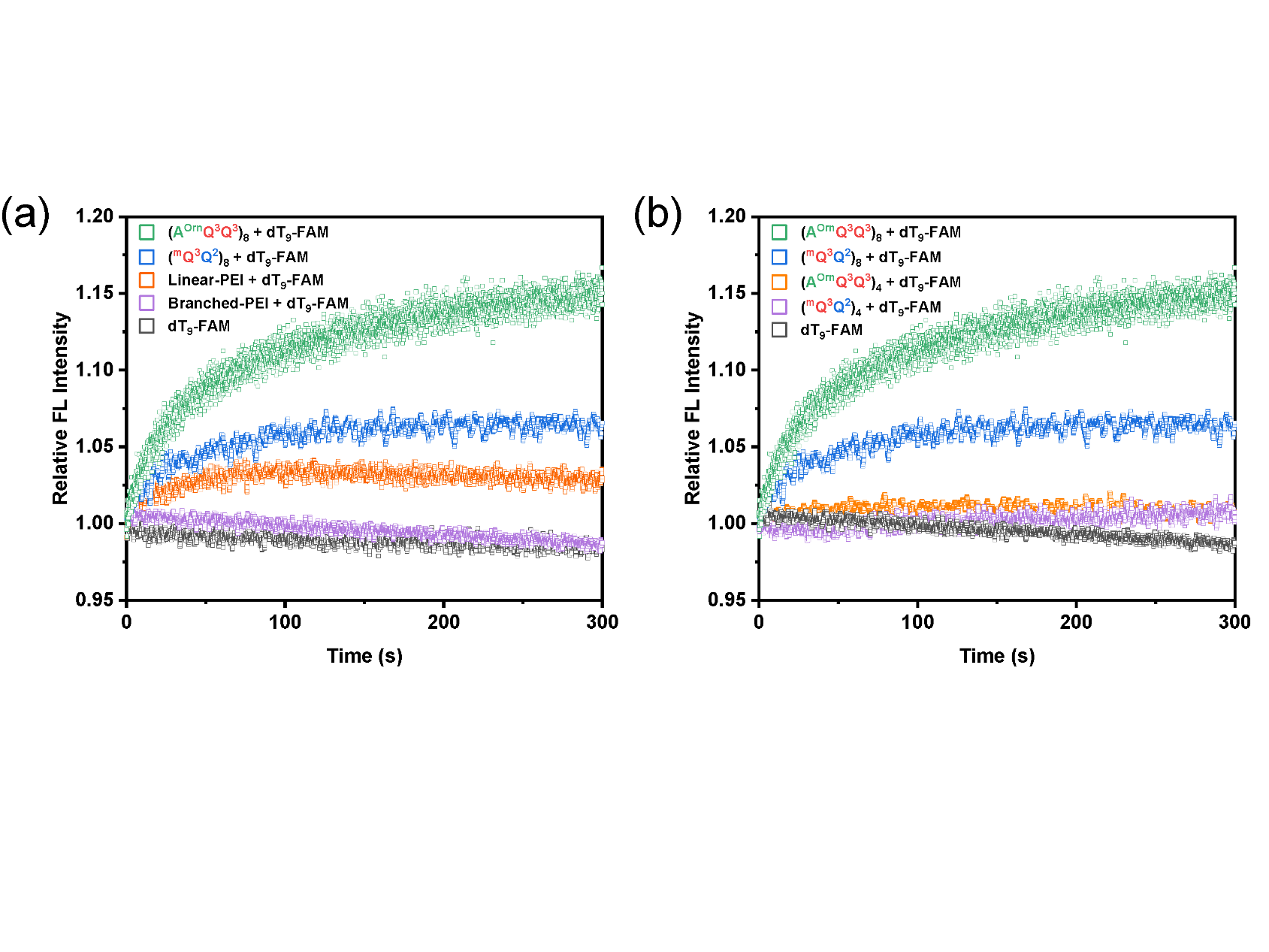


Figure S18. Normalized dT_9_-FAM (5’- FAM-TTT TTT TTT-3’) transport activities of (a) linear-PEI and branched-PEI, (b) (^m^Q^3^Q^2^)_4_ and (A^Orn^Q^3^Q^3^)_4_ for at the same concentration of foldamers (1 μM).

**Bilayer lipid membrane (BLM) experiments**

A chloroform solution of EYPC was dried using nitrogen gas to form a thin film and then dissolved in n-decane (25 mg/mL). 0.5 μL of the *n*-decane solution was precoated on the aperture (diameter = 200 μm) of the Delrin® cup, followed by removing the solvent with nitrogen gas. Both the cup (reference electrode) and chamber (input electrode) on the two side of the aperture were filled with 1.0 mL of 10 mM Tris·HCl, pH = 7.4. Ag-AgCl electrodes were applied directly to the two solutions. Planar lipid bilayer was formed by painting 0.3 μL of the lipid solution around the pretreated aperture and judged by capacitance (80-120 pF). The mixed solution of dT_9_ (2 μL, 50 μM) and foldamers (1 μL, 50 μM) in 10 mM Tris·HCl, pH = 7.4 was added into the cup and stirred for 3 minutes. The currents were obtained by a Warner BC-535 bilayer clamp amplifier (Warner Instruments) and collected using the ML846 data acquisition system. All data was filtered at 1 KHz with 8-pole Bessel fillter.

**
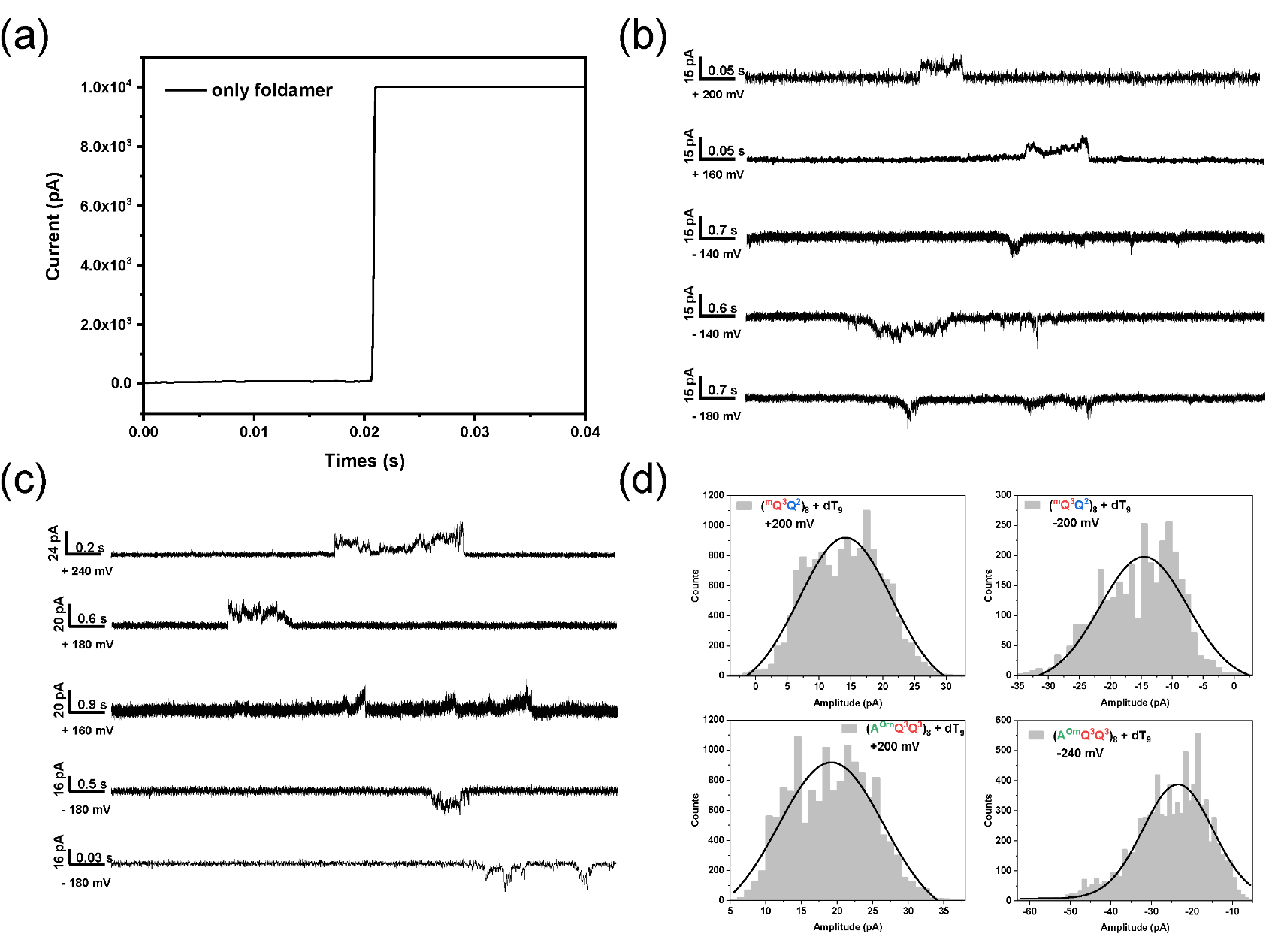
**

Figure S19. (a) Current traces of (^m^Q^3^Q^2^)_8_ or (A^Orn^Q^3^Q^3^)_8_ under different voltages in symmetrical solution (*cis* chamber = *trans* chamber = 10 mM Tris·HCl, pH = 7.4). Current traces of (b) (^m^Q^3^Q^2^)_8_ and (c) (A^Orn^Q^3^Q^3^)_8_ with dT_9_ (5’-TTT TTT TTT-3’) under different voltages in symmetrical solution (*cis* chamber = *trans* chamber = 10 mM Tris·HCl, pH = 7.4). (d) Histogram of the currents of (^m^Q^3^Q^2^)_8_ and (A^Orn^Q^3^Q^3^)_8_ with dT_9_ (5’-TTT TTT TTT-3’) under different voltages fitted with a single Gaussian fit to extract the average current.

**Cell culture and Cytotoxicity assays**

Human Umbilical Vein Endothelial Cells (HUVEC) were cultured in Dulbecco’s modified Eagle’s medium (DMEM; Gibco, Carlsbad, CA, USA) containing 10% (v/v) fetal bovine serum (FBS; HyClone Laboratories, Inc., Logan, UT, USA) and 1% (v/v) penicillin-streptomycin solution in an incubator with 5% CO_2_ at 37 ℃. Cells were cultured in plastic flasks until the confluency was greater than 90% and detached by 0.25 wt% Trypsin-EDTA solution. DMEM medium containing FBS was used to terminate the detachment of cells. The cell suspension was then centrifuged at 1200 rpm for 3 min and dispersed in a fresh medium at a suitable density for cell passaging.

HUVEC were seeded in 96-well plates at the density of 10,000 cells/well overnight. And then the culture medium was discarded and replaced with fresh DMEM containing different concentrations of (^m^Q^3^Q^2^)_8_ and (A^Orn^Q^3^Q^3^)_8_. Hieff Trans Liposomal Transfection Reagent (Yaesen, 1 μL) was used as positive control. Cell viability was determined by quantification of 3-(4,5-dimethylthiazol-2-yl)-2,5-diphenyltetrazolium bromide (MTT) reduction by mitochondrial dehydrogenases. The MTT assay was used to detect the cell viability after 24 h of exposure to different concentration of foldamers. Specifically, 10 μL MTT solution (5mg/ml) was added to each well, and then the plates were incubated at 37 ℃ for 2 hours, finally the absorbance was detected at 570 nm. Cell viability was calculated as follow:

Cell viability = (OD_sample_ – OD_blank_) / (OD_control_ – OD_blank_)

Figure S20. MTT cell viability assay in Human Umbilical Vein Endothelial Cells treat with (^m^Q^3^Q^2^)_8_ and (A^Orn^Q^3^Q^3^)_8_ at different concentrations. Hieff Trans Liposomal Transfection Reagent (1 uL) was used as positive control.

**EGFP-mRNA transfection and imaging by laser scanning** **confocal microscope**

*In* *vitro* cell transfection experiments have been performed using the EGFP-mRNA (Absin) which is for expression of enhanced green fluorescent protein. Twenty-four hours prior to transfection, 2×10^5^ Human Umbilical Vein Endothelial Cells were seeded in 35mm confocal glass-bottom dishes (Beyotime). Specifically, 1 uL of mRNA (1 μg/μL) and 1 uL of Hieff Trans Liposomal Transfection Reagent (Yaesen) or 1 uL foldamers (10 μM) were mixed. The mixture was diluted with serum-free medium to a final volume of 50 μL and incubated for 20 min at room temperature. For transfection, culture media was removed and replaced by 2 mL of serum-free DMEM, the mixture was added to 35mm confocal glass-bottom dishes with cells at 50-60% confluence. After incubation for 2.5 h at 37℃, the medium was replaced with fresh one contain serum. EGFP expression was imaged 6 hours after transfection by laser scanning confocal microscope (AX, Nikon, Japan). Imaging of mRNA expression was performed using a Nikon AX confocal laser scanning microscope, using 405 nm laser excitation for foldamers, 488 nm laser excitation for EGFP.


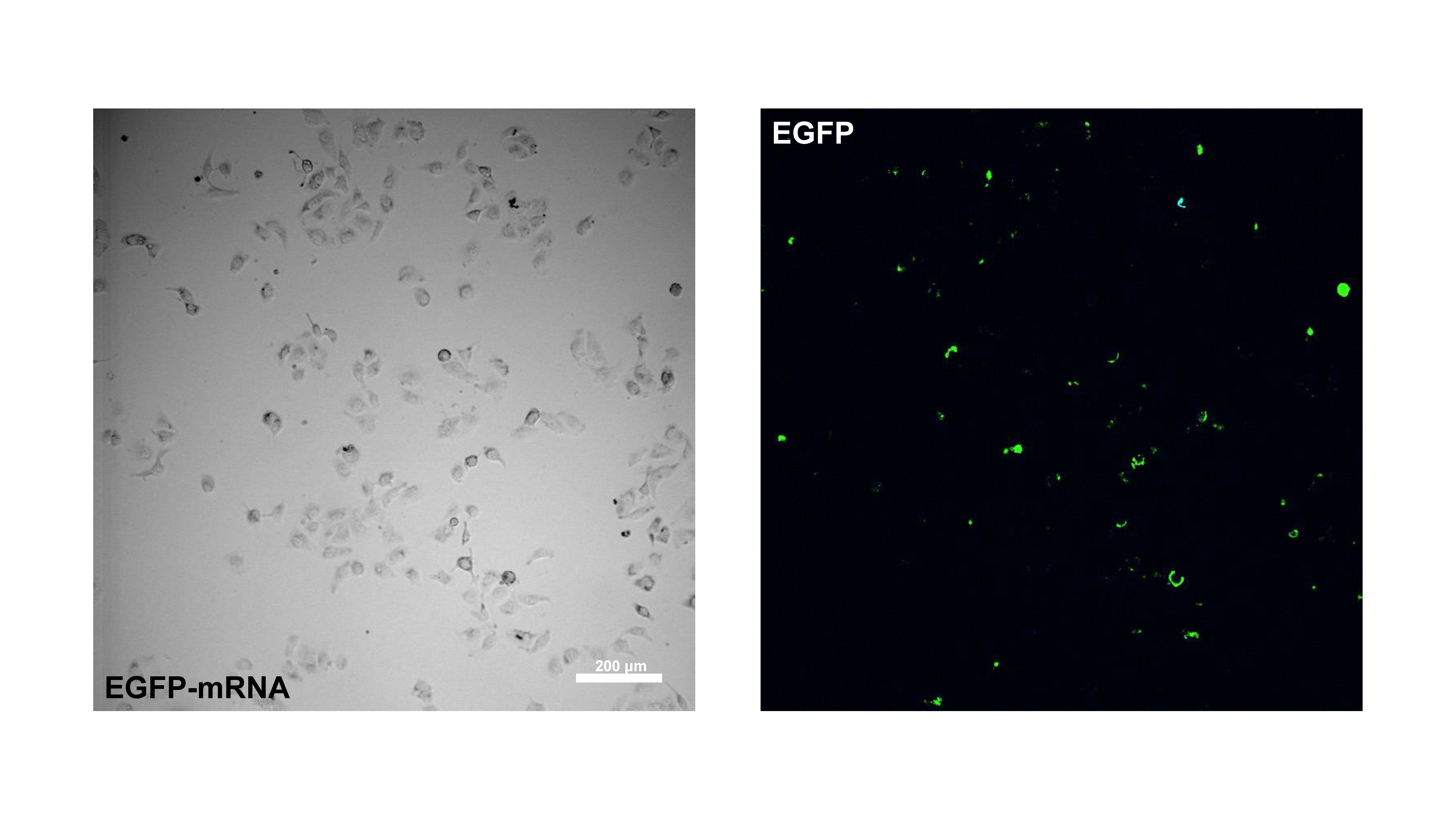


Figure S21. Confocal laser scanning microscopy images (10×) of negative control in Human Umbilical Vein Endothelial Cells.


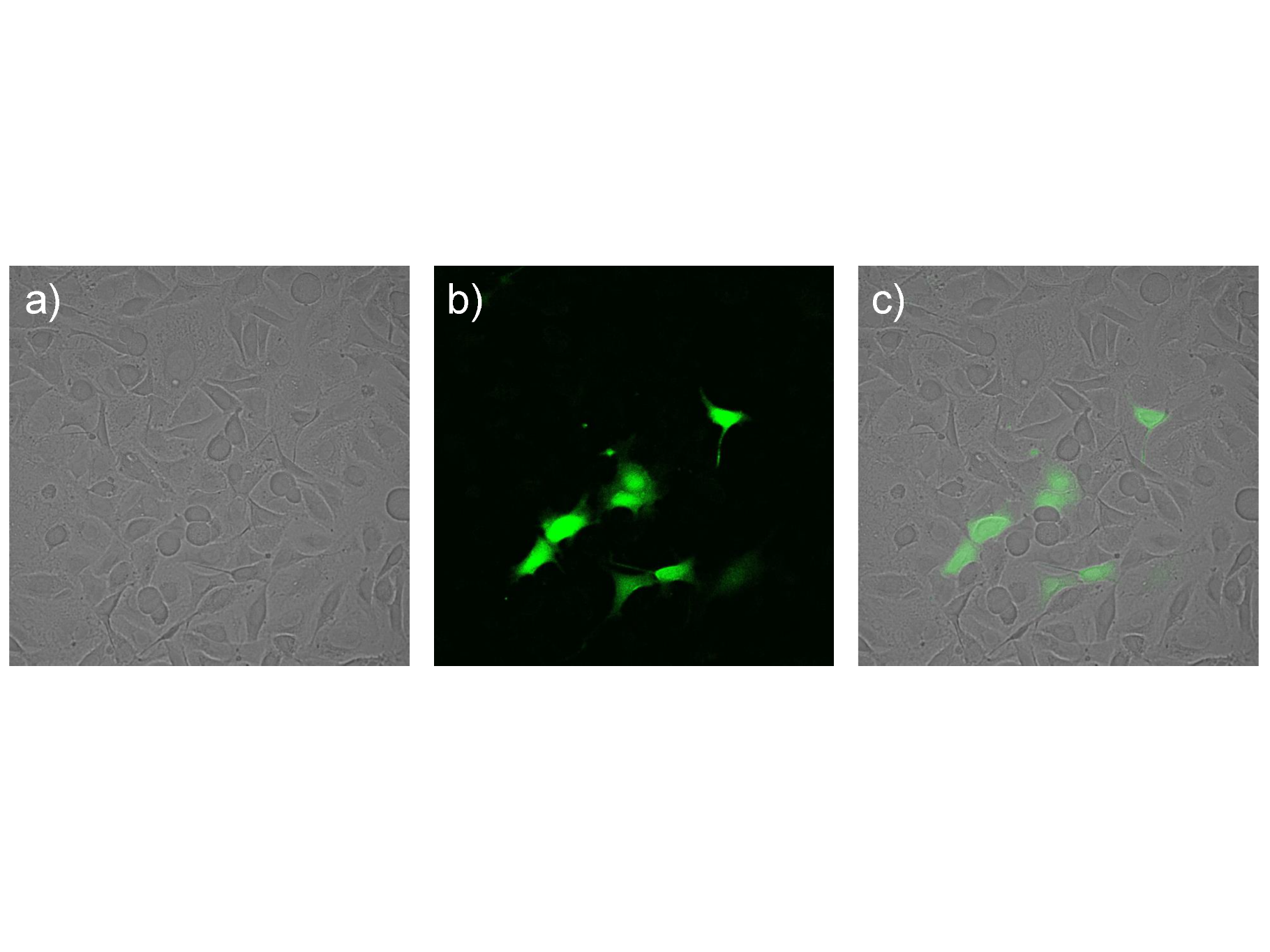


Figure S22. Confocal laser scanning microscopy images (40×) of positive control (Hieff Trans Liposomal Transfection Reagent carrying EGFP-mRNA) in Human Umbilical Vein Endothelial Cells.


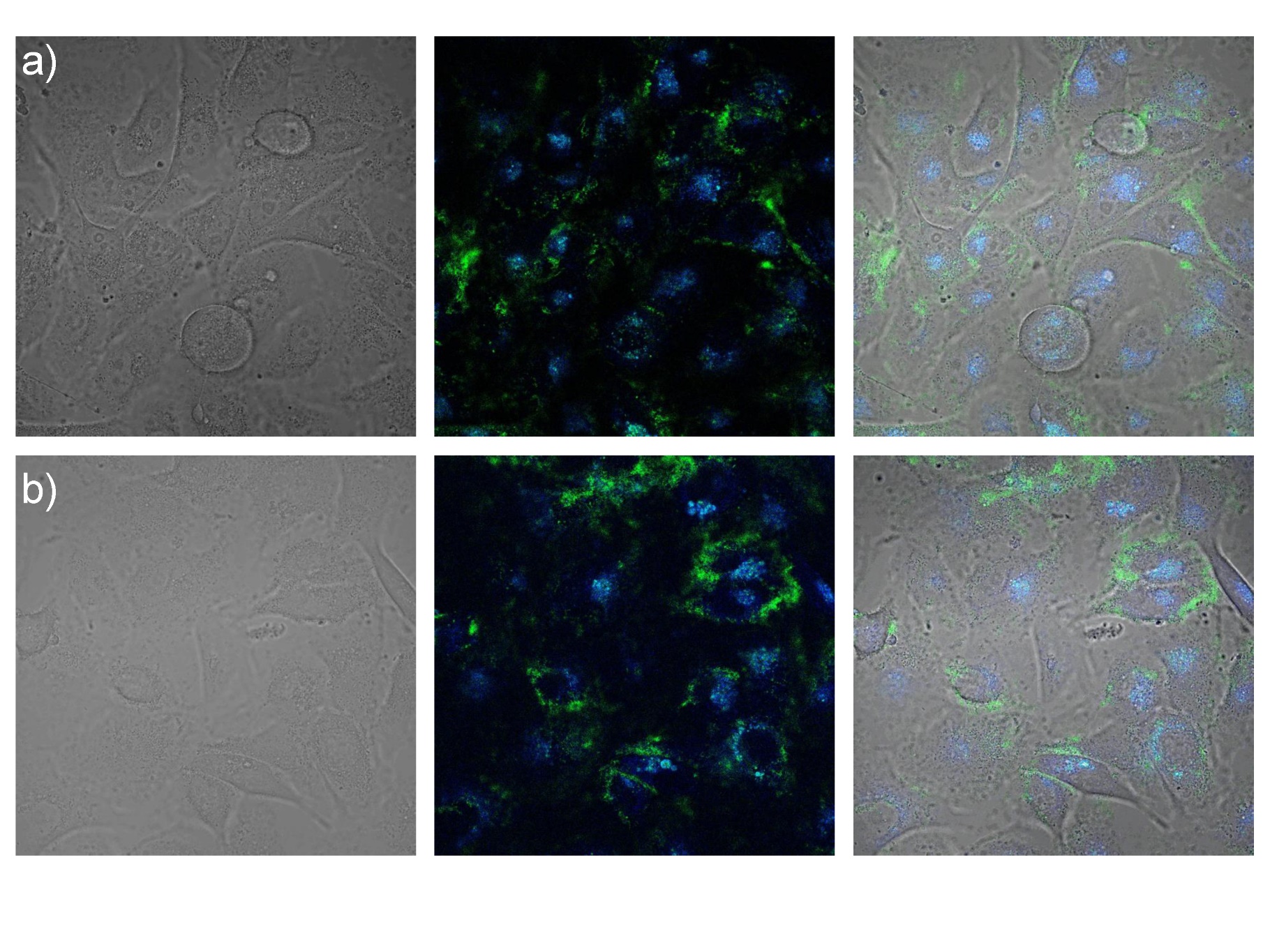


Figure S23. Confocal laser scanning microscopy images (100×) of Human Umbilical Vein Endothelial Cells after transfection with (a) (A^Orn^Q^3^Q^3^)_8_ and (b) (^m^Q^3^Q^2^)_8_ carrying EGFP-mRNA at the concentration of 10 μM. (405 nm laser excitation for foldamers (blue fluorescence), 488 nm laser excitation for EGFP (green fluorescence).


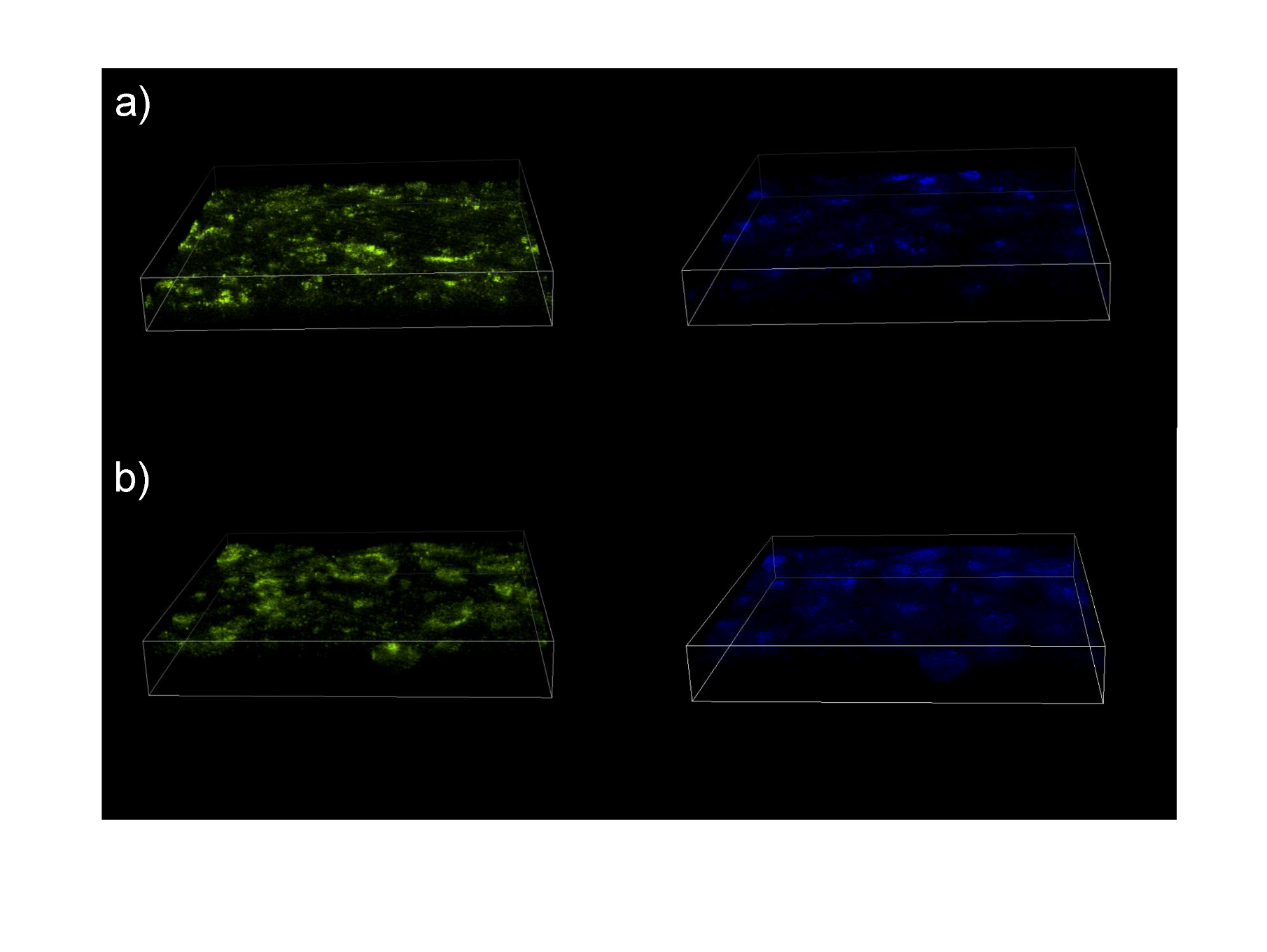


Figure S24. 3D images of cells at 6 h of transfection by (a) (A^Orn^Q^3^Q^3^)_8_ and (b) (^m^Q^3^Q^2^)_8_. (405 nm laser excitation for foldamers (blue fluorescence), 488 nm laser excitation for EGFP (green fluorescence).

**Synthesis and Characteristic spectra of compounds**

**Compound 3**

Compound 2^[2]^ (10.0 g, 46.0 mmol), triphenylphosphine (PPh_3_, 11.2 g, 1.05 equiv.) and 2-isopropoxyethan-1-ol (5.3 g, 1.1 equiv.) were dissolved in 90 mL anhydrous tetrahydrofuran (THF), respectively. And then diisopropyl azodiformate (DIAD) was added dropwise at the condition of ice bath and the protection of nitrogen gas^[3]^. After stirring for 4 hours at room temperature, rotary evaporating the solvent and then the residues were dispersed in methanol to recrystallize in refrigerator at -20°C for one night. After filtering the precipitant with a Buchner funnel, compound 3 was obtained as white solid (11.0 g, 80% yield). ^1^H NMR (500 MHz, DMSO-d6) δ 8.06 – 8.02 (m, 1H), 7.71 (dt, J = 6.9, 1.3 Hz, 1H), 7.61 – 7.57 (m, 2H), 4.46 – 4.39 (m, 2H), 3.96 (s, 3H), 3.90 – 3.85 (m, 2H), 3.71 (p, J = 6.1 Hz, 1H), 2.74 (s, 3H), 1.14 (d, J = 6.0 Hz, 6H). ^13^C NMR (125 MHz, DMSO) δ 166.18, 162.59, 148.37, 147.33, 137.93, 131.19, 127.96, 121.95, 119.77, 101.38, 71.68, 69.32, 66.10, 53.13, 22.53, 18.35, 0.57. ESI m/z: calculated for C_17_H_21_NO_4_ [M+H]^+^ 304.1; Found 304.2.

Figure S25. ^1^H NMR spectrum of 3 in DMSO-d_6_.

Figure S26. ^13^C NMR spectrum of 3 in DMSO-d_6_.

Figure S27. ESI-MS spectrum of 3.

**Compound 4**

Compound 3 (9.8 g, 32.3 mmol), bromosuccinimide (NBS, 6.3 g, 35.5 mmol) and BPO (8.5 g, 35.5 mmol) were added into 50 mL benzene solution, stir and reflux in oil bath at 80 °C for 4 hours. Rotary evaporate solvent and the residues were purified by silica gel chromatography using pure CH_2_Cl_2_ as eluent. After drying under reduced pressure, compound 4 was obtained as yellow solid (7.0 g, 70% yield). ^1^H NMR (500 MHz, DMSO-d_6_) δ 8.20 (dd, J = 8.4, 1.5 Hz, 1H), 8.05 (dd, J = 7.1, 1.5 Hz, 1H), 7.71 (dd, J = 8.4, 7.0 Hz, 1H), 7.64 (s, 1H), 5.31 (s, 2H), 4.52 – 4.44 (m, 2H), 3.98 (s, 3H), 3.91 – 3.85 (m, 2H), 3.71 (h, J = 6.1 Hz, 1H), 1.14 (d, J = 6.1 Hz, 6H). ^13^C NMR (125 MHz, DMSO) δ 165.96, 162.82, 149.15, 145.80, 137.14, 132.72, 128.13, 122.79, 122.14, 102.01, 71.69, 69.57, 66.06, 53.31, 30.19, 22.53. ESI m/z: calculated for C_17_H_20_BrNO_4_ [M+H]^+^ 382.1; Found 382.1.

Figure S28. ^1^H NMR spectrum of 4 in DMSO-d_6_.

Figure S29. ^13^C NMR spectrum of 4 in DMSO-d_6_.

Figure S30. ESI-MS spectrum of 4.

**Compound 5**

Compound 4 (5.0 g, 13.1 mmol) and NaN_3_ (936.6 mg, 14.4 mmol) were added to DMF (40 mL) and stirred at room temperature for 6 hours. Rotary evaporating the solvent, the residues were purified by silica gel chromatography. Compound 5 was obtained as yellow oily substance (3.6 g, 79% yield). ^1^H NMR (500 MHz, Chloroform-*d*) δ 8.27 (dd, *J* = 8.5, 1.4 Hz, 1H), 7.79 (d, *J* = 6.9 Hz, 1H), 7.65 – 7.57 (m, 2H), 5.14 (s, 2H), 4.44 (t, *J* = 4.9 Hz, 2H), 4.06 (s, 3H), 3.97 (t, *J* = 4.9 Hz, 2H), 3.77 (hept, *J* = 6.1 Hz, 1H), 1.26 (d, *J* = 6.1 Hz, 6H). ^13^C NMR (125 MHz, CDCl_3_) δ 166.22, 162.72, 148.53, 146.54, 135.11, 129.71, 127.11, 122.34, 122.26, 101.23, 72.37, 68.74, 66.03, 52.97, 51.00, 22.09. ESI m/z: calculated for C_17_H_20_N_4_O_4_ [M+H]^+^ 345.1; Found 345.2.

Figure S31. ^1^H NMR spectrum of 5 in CDCl_3_.

Figure S32. ^13^C NMR spectrum of 5 in CDCl_3_.

Figure S33. ESI-MS spectrum of 5.

**Compound 6**

Compound 5 (6.0 g, 17.4 mmol) and PPh_3_ (6.9 g, 26.0 mmol) were dissolved in THF (200 mL). After stirring for 1.5 hours at room temperature, inject 10 mL distilled water into the reaction system by a syringe and stir for 12 hours. *N*-(Benzyloxycarbonyloxy) succinimide (6.5 g, 26.0 mmol) and excess TEA (20 mL) were added to the mixture solvent above, and kept reacting 6 hours at room temperature. The residues were dried under reduced pressure after rotary evaporating the solvent. And then the residues were purified by silica gel chromatography (CH_2_Cl_2_/EtOAc = 10:1 as eluent) to give compound 6 (4.5 g, 57% yield). ^1^H NMR (500 MHz, DMSO-*d*_6_) δ 8.10 (dd, *J* = 7.1, 2.8 Hz, 1H), 7.85 (t, *J* = 6.2 Hz, 1H), 7.72 – 7.66 (m, 2H), 7.62 (s, 1H), 7.44 – 7.29 (m, 5H), 5.10 (s, 2H), 4.90 (d, *J* = 6.2 Hz, 2H), 4.49 – 4.42 (m, 2H), 3.96 (s, 3H), 3.90 – 3.84 (m, 2H), 3.71 (hept, *J* = 6.1 Hz, 1H), 1.14 (d, *J* = 6.1 Hz, 6H). ^13^C NMR (125 MHz, DMSO) δ 166.00, 162.67, 157.05, 148.54, 145.92, 138.49, 137.66, 128.85, 128.28, 128.22, 127.94, 127.77, 121.78, 120.58, 101.67, 71.69, 69.39, 66.08, 65.96, 53.20, 22.52. ESI m/z: calculated for C_25_H_28_N_2_O_6_ [M+H]^+^ 453.2; Found 453.2.

Figure S34. ^1^H NMR spectrum of 6 in DMSO-d_6_.

Figure S35. ^13^C NMR spectrum of 6 in DMSO-d_6_.

Figure S36. ESI-MS spectrum of 6.

**Compound 7**

Compound 6 (4.0 g, 8.8 mmol) and LiOH (422.0 mg, 17.6 mmol) were dissolved in methanol and stirred for 2 hours, and rotary evaporate the solvent. Then acidification and extraction were carried out, respectively. Compound 7 was obtained as white powder (3.5 g, 90% yield). ^1^H NMR (500 MHz, DMSO-*d*_6_) δ 8.11 (dd, *J* = 8.2, 1.7 Hz, 1H), 7.90 (t, *J* = 6.2 Hz, 1H), 7.74 – 7.70 (m, 1H), 7.70 – 7.64 (m, 1H), 7.62 (s, 1H), 7.42 – 7.29 (m, 5H), 5.08 (s, 2H), 4.88 (d, *J* = 6.2 Hz, 2H), 4.45 (dd, *J* = 5.6, 3.4 Hz, 2H), 3.90 – 3.84 (m, 2H), 3.71 (hept, *J* = 6.1 Hz, 1H), 1.14 (d, *J* = 6.1 Hz, 6H). ^13^C NMR (125 MHz, DMSO) δ 167.96, 162.43, 157.17, 156.45, 145.60, 137.58, 137.46, 128.82, 128.28, 128.24, 126.52, 121.47, 120.81, 101.57, 71.67, 69.02, 66.07, 66.01, 41.24, 22.54. ESI m/z: calculated for C_24_H_26_N_2_O_6_ [M+H]^+^ 439.2; Found 439.2.

Figure S37. ^1^H NMR spectrum of 7 in DMSO-d_6_.

Figure S38. ^13^C NMR spectrum of 7 in DMSO-d_6_.

Figure S39. ESI-MS spectrum of 7.

**Compound 10**

Compound 9^[2]^ (10.0 g, 40.0 mmol), triphenylphosphine (PPh_3_, 11.2 g, 1.05 equiv.), tert-butyl (2-hydroxyethyl) carbamate (7.7 g, 1.2 equiv.) were mixed in 90 mL anhydrous tetrahydrofuran (THF). And then diisopropyl azodiformate (DIAD) was added dropwise at the condition of ice bath and the protection of nitrogen gas. After stirring for 4 hours at room temperature, rotary evaporating the solvent and then the residues were dispersed in methanol to recrystallize in refrigerator at -20°C for one night. After filtering the precipitant with a Buchner funnel, compound 10 was obtained as white solid (12.0 g, 75% yield). ^1^H NMR (500 MHz, DMSO-*d*_6_) δ 8.56 (dd, *J* = 8.5, 1.4 Hz, 1H), 8.35 (dd, *J* = 7.6, 1.4 Hz, 1H), 7.83 (t, *J* = 8.0 Hz, 1H), 7.67 (s, 1H), 7.22 (t, *J* = 5.9 Hz, 1H), 4.38 (t, *J* = 5.2 Hz, 2H), 3.95 (s, 3H), 3.51 (q, *J* = 5.4 Hz, 2H), 1.38 (s, 9H). ^13^C NMR (125 MHz, DMSO) δ 165.28, 162.76, 156.28, 151.45, 148.69, 139.21, 127.22, 126.47, 124.95, 122.79, 103.02, 78.37, 69.39, 53.45, 28.66. ESI m/z: calculated for C_18_H_21_N_3_O_7_ [M+H]^+^ 392.1; Found 392.1.

Figure S40. ^1^H NMR spectrum of 10 in DMSO-d_6_.

Figure S41. ^13^C NMR spectrum of 10 in DMSO-d_6_.

Figure S42. ESI-MS spectrum of 10.

**Compound 11**

The nitro precursor 10 (5.0 g, 12.7 mmol) and ammonium formate (1.6 g, 2.0 equiv.) were dissolved in the mixture solution of EtOAc (25 mL) and MeOH (25 mL), and 25 mg of 10 wt % Pd/C was added. The reaction was stirred at room temperature for 4 hours. Upon completion the catalyst was removed by filtration over celite and the filtrate was evaporated to provide amine 11 with quantitative yield. ^1^H NMR (500 MHz, DMSO-*d*_6_) δ 7.45 (s, 1H), 7.40 – 7.31 (m, 2H), 7.13 (t, *J* = 5.9 Hz, 1H), 6.93 (dd, *J* = 7.1, 1.8 Hz, 1H), 6.00 (s, 2H), 4.25 (t, *J* = 5.4 Hz, 2H), 3.94 (s, 3H), 3.46 (p, *J* = 5.5 Hz, 2H), 1.39 (s, 9H). ^13^C NMR (125 MHz, DMSO) δ 165.99, 162.18, 156.26, 146.40, 145.58, 137.65, 129.30, 122.70, 110.32, 107.99, 100.99, 78.28, 68.21, 52.98, 28.68. ESI m/z: calculated for C_18_H_23_N_3_O_5_ [M+H]^+^ 362.2; Found 362.2.

Figure S43. ^1^H NMR spectrum of 11 in DMSO-d_6_.

Figure S44. ^13^C NMR spectrum of 11 in DMSO-d_6_.

Figure S45. ESI-MS spectrum of 11.

**Compound 12**

Compound 7 (4.0 g, 9.1 mmol) and compound 11 (3.2 g, 10.6 mmol) were dissolved in anhydrous 50 mL DMF, and then PyBop (7.0 g, 13.6 mmol) and excess DIPEA (10 mL) were added into this reaction system. After stirring in room temperature 4 hours, rotary evaporating the solvent, the residues were dispersed in cold 100 mL methanol, and then filter the precipitant, compound 12 was obtained as white solid after drying in reduced pressure (5.0 g, 70% yield). ^1^H NMR (500 MHz, Chloroform-*d*) δ 12.29 (s, 1H), 9.14 (d, *J* = 7.7 Hz, 1H), 8.25 (d, *J* = 8.4 Hz, 1H), 7.95 (d, *J* = 8.3 Hz, 1H), 7.90 (d, *J* = 7.0 Hz, 1H), 7.83 (s, 1H), 7.67 (t, *J* = 8.1 Hz, 1H), 7.58 (t, *J* = 7.7 Hz, 1H), 7.52 (s, 1H), 7.23 (td, *J* = 7.1, 3.6 Hz, 5H), 6.10 (t, *J* = 6.5 Hz, 1H), 5.27 (d, *J* = 6.4 Hz, 2H), 5.11 (s, 1H), 5.03 (s, 2H), 4.49 (t, *J* = 4.7 Hz, 2H), 4.35 (t, *J* = 5.0 Hz, 2H), 4.01 (d, *J* = 10.5 Hz, 5H), 3.84 – 3.74 (m, 3H), 1.50 (s, 9H), 1.28 (d, *J* = 6.1 Hz, 7H). ^13^C NMR (125 MHz, CDCl_3_) δ 165.65, 163.43, 163.13, 162.31, 156.88, 155.88, 150.63, 147.58, 145.97, 139.56, 136.93, 136.67, 135.19, 130.47, 129.78, 128.44, 128.33, 127.91, 127.86, 126.96, 122.39, 121.91, 121.75, 117.92, 115.90, 101.49, 98.60, 79.94, 72.39, 68.77, 68.41, 66.79, 66.44, 66.07, 53.05, 42.48, 39.83, 28.40, 22.14. ESI m/z: calculated for C_42_H_47_N_5_O_10_ [M+H]^+^ 782.3; Found 782.3.

Figure S46. ^1^H NMR spectrum of 12 in CDCl_3_.

Figure S47. ^13^C NMR spectrum of 12 in CDCl_3_.

Figure S48. ESI-MS spectrum of 12.

**Compound 13**

Compound 12 (2.0 g, 2.5 mmol) and LiOH (120.0 mg, 5.0 mmol) was added to methanol and stirred for 2 hours, and rotary evaporated the solvent. Then acidification and extraction were carried out respectively. compound 13 as white powder (1.8 g, 92% yield) was used without further purification after reduced pressure drying. ESI m/z: calculated for C_41_H_45_N_5_O_10_ [M+H]^+^ 768.3; Found 768.3.

Figure S49. ESI-MS spectrum of 13.

**Compound 14**

Compound 12 (2.0 g, 2.5 mmol) and ammonium formate (315.0 mg, 5.0 mmol) were dissolved in the mixture solution of DCM (10 mL) and MeOH (10 mL), and 25 mg of 10 wt % Pd/C was added. The reaction was stirred at room temperature for 4 hours. Upon completion the catalyst was removed by filtration over celite and the filtrate was evaporated to provide the amine 14 that was used without further purification. ESI m/z: calculated for C_34_H_41_N_5_O_8_ [M+H]^+^ 648.3; Found 648.3.

Figure S50. ESI-MS spectrum of 14.

**Compound 15**

Compound 13 (1.5 g, 1.9 mmol) and compound 14 (1.2 g, 1.9 mmol) were dissolved in anhydrous 25 mL DMF, and then PyBop (1.8 g, 3.8 mmol) and excess DIPEA (5 mL) were added into this reaction system. After stirring in room temperature 4 hours later, rotary evaporating the solvent, the residues were dispersed in cold 60 mL methanol, and then filter the precipitant, compound 15 was obtained as white solid after drying in reduced pressure (1.7 g, 63% yield). ^1^H NMR (400 MHz, Chloroform-*d*) δ 11.32 (s, 1H), 11.15 (s, 1H), 10.50 (s, 1H), 10.29 (s, 1H), 9.51 (d, *J* = 10.0 Hz, 1H), 9.28 (s, 1H), 8.99 (d, *J* = 8.9 Hz, 1H), 8.57 (d, *J* = 7.4 Hz, 1H), 8.04 (s, 2H), 7.98 (t, 2H), 7.84 (t, 2H), 7.76 (d, *J* = 9.8 Hz, 2H), 7.65 (t, 2H), 7.59 (s, 2H), 7.46 (t, *J* = 7.6 Hz, 1H), 7.20 (d, *J* = 8.3 Hz, 2H), 7.13 (s, 5H), 7.08 (d, *J* = 8.2 Hz, 2H), 7.02 (s, 2H), 6.78 (d, *J* = 13.2 Hz, 4H), 6.67 (d, *J* = 11.7 Hz, 6H), 6.57 (d, *J* = 7.8 Hz, 2H), 6.51 (s, 2H), 6.46 (s, 2H), 6.41 (s, 1H), 5.67 (s, 2H), 5.39 (t, *J* = 12.2 Hz, 1H), 5.04 (d, *J* = 13.7 Hz, 1H), 4.76 (d, *J* = 12.3 Hz, 1H), 4.62 (d, *J* = 12.4 Hz, 1H), 4.46 (d, *J* = 13.5 Hz, 4H), 4.29 (d, *J* = 19.2 Hz, 8H), 4.00 (s, 9H), 3.94 (s, 3H), 3.81 (dd, *J* = 11.8, 5.7 Hz, 12H), 3.70 (d, *J* = 5.6 Hz, 8H), 1.61 (s, 19H), 1.50 (s, 18H), 1.42 (d, 6H), 1.35 (dd, 12H), 1.25 (d, 6H). ^13^C NMR (125 MHz, DMSO) δ 165.06, 162.63, 161.81, 161.29, 156.87, 155.71, 150.76, 150.60, 149.91, 146.30, 146.07, 138.66, 137.99, 137.42, 137.26, 136.38, 133.99, 132.70, 132.43, 128.59, 128.07, 127.30, 123.07, 121.35, 120.58, 120.40, 119.94, 119.34, 117.44, 116.21, 113.81, 104.45, 100.59, 100.49, 99.37, 98.81, 97.83, 78.82, 71.89, 71.76, 69.61, 68.48, 66.11, 65.88, 65.57, 53.80, 44.26, 28.81, 28.70, 22.62, 22.54. ESI m/z: calculated for C_75_H_84_N_10_O_17_ [M+H]^+^ 1397.6; Found 1397.6.

Figure S51. ^1^H NMR spectrum of 15 in CDCl_3_.

Figure S52. ^13^C NMR spectrum of 15 in DMSO-d_6_.

Figure S53. ESI-MS spectrum of 15.

**Compound 16**

Compound 15 (0.8 g, 0.57 mmol) and LiOH (27.0 mg, 1.14mmol) were added to methanol and stirred for 2 hours, and rotary evaporated the solvent. Then acidification and extraction were carried out respectively. Compound 16 as white powder (0.7 g, 88% yield) after reduced pressure drying. ESI m/z: calculated for C_74_H_82_N_10_O_17_ [M+H]^+^ 1383.6; Found 1383.6.

Figure S54. ESI-MS spectrum of 16.

**Compound 17**

The compound 15 (0.8 g, 0.57 mmol) and ammonium formate (72.0 mg, 1.14mmol) were dissolved in the mixture solution of DCM (5 mL) and MeOH (5 mL), and 25 mg of 10 wt % Pd/C was added. The reaction was stirred at room temperature for 4 hours. Upon completion the catalyst was removed by filtration over celite and the filtrate was evaporated to provide the amine 17 with quantitative yield. ESI m/z: calculated for C_67_H_78_N_10_O_15_ [M+H]^+^ 1263.6; Found 1263.6.

Figure S55. ESI-MS spectrum of 17.

**Compound 18**

Compound 16 (0.6 g, 0.43 mmol) and compound 17 (0.5 mg, 0.4 mmol) were dissolved in anhydrous 10 mL DMF, and then PyBop (447.0 mg, 0.86 mmol) and excess DIPEA (3 mL) were added into this reaction system. After stirring in room temperature 4 hours, rotary evaporating the solvent, the residues were dispersed in cold methanol, and then filtered the precipitant, compound 18 was obtained as white solid after drying in reduced pressure (0.7 g, 62% yield). ^1^H NMR (500 MHz, DMSO-*d*_6_) δ 11.20 (s, 1H), 11.05 (s, 1H), 10.45 (s, 1H), 10.11 (s, 1H), 9.26 (d, *J* = 26.8 Hz, 2H), 8.82 (s, 1H), 8.40 (d, *J* = 7.5 Hz, 1H), 8.02 (s, 1H), 7.95 – 7.87 (m, 2H), 7.70 (d, *J* = 15.5 Hz, 2H), 7.60 – 7.56 (m, 1H), 7.49 (s, 3H), 7.30 (d, *J* = 8.5 Hz, 4H), 7.23 (s, 2H), 7.11 (d, *J* = 23.5 Hz, 11H), 7.02 (s, 3H), 6.92 (s, 1H), 6.70 – 6.64 (m, 5H), 6.58 (s, 2H), 6.52 (s, 1H), 6.41 (d, *J* = 17.0 Hz, 3H), 6.33 (s, 1H), 5.20 (s, 1H), 4.86 (s, 1H), 4.62 (s, 2H), 4.55 (s, 1H), 4.26 (s, 1H), 4.10 (q, *J* = 5.3 Hz, 6H), 4.05 (s, 5H), 3.99 – 3.95 (m, 3H), 3.89 – 3.81 (m, 8H), 3.77 (s, 4H), 3.71 – 3.68 (m, 4H), 3.51 (s, 8H), 3.17 (d, *J* = 5.2 Hz, 4H), 2.93 (s, 1H), 1.57 (d, *J* = 10.7 Hz, 28H), 1.43 (s, 9H), 1.40 (d, *J* = 6.1 Hz, 6H), 1.32 (d, *J* = 6.1 Hz, 6H), 1.29 (d, *J* = 6.2 Hz, 6H), 1.19 (d, *J* = 6.1 Hz, 6H). ^13^C NMR (100 MHz, CDCl_3_) δ 165.44, 165.23, 164.26, 164.17, 162.69, 162.56, 162.42, 161.99, 161.64, 161.51, 161.41, 161.24, 160.87, 160.57, 160.33, 156.84, 156.51, 155.83, 150.94, 150.70, 150.48, 149.71, 149.54, 146.76, 145.57, 145.48, 145.22, 144.23, 137.72, 137.29, 136.35, 136.16, 136.03, 135.30, 134.59, 133.98, 133.12, 132.58, 131.72, 131.57, 131.36, 130.16, 129.77, 128.63, 128.23, 127.88, 127.74, 127.02, 126.67, 126.46, 126.00, 123.25, 122.47, 122.17, 121.52, 121.41, 121.33, 121.20, 120.38, 120.18, 119.80, 118.92, 118.20, 117.23, 117.01, 116.76, 115.93, 115.16, 114.81, 100.08, 99.56, 99.13, 98.07, 97.72, 97.31, 79.88, 79.47, 72.35, 72.26, 72.20, 72.16, 68.76, 68.28, 68.11, 67.99, 67.77, 67.38, 66.24, 66.13, 65.94, 65.78, 53.82, 44.03, 43.62, 43.16, 41.00, 40.10, 39.67, 36.52, 31.47, 29.03, 28.89, 28.61, 22.39, 22.32, 22.29, 22.17, 0.04. ESI m/z: calculated for C_141_H_158_N_20_O_31_ [M+H]^+^ 2628.1; Found 2628.0.

Figure S56. ^1^H NMR spectrum of 18 in DMSO.

Figure S57. ^13^C NMR spectrum of 18 in CDCl_3_.

Figure S58. ESI-MS spectrum of 18.

**Compound 19**

Compound 18 (0.3 g, 0.11 mmol) and LiOH (10.0 mg, 0.4 mmol) were added to methanol and stirred for 2 hours, and rotary evaporated the solvent. Then acidification and extraction were carried out respectively. Compound 19 as white powder (250.0 mg, 86% yield) after reduced pressure drying. ESI m/z: calculated for C_140_H_156_N_20_O_31_ [M+H]^+^ 2614.1; Found 2614.0.

Figure S59. ESI-MS spectrum of 19.

**Compound 20**

The compound 18 (0.3 g, 0.11 mmol) and ammonium formate (25.0 mg, 0.4 mmol) were dissolved in the mixture solution of DCM (5 mL) and MeOH (5 mL), and 10 mg of 10 wt % Pd/C was added. The reaction was stirred at room temperature for 4 hours. Upon completion the catalyst was removed by filtration over celite and the filtrate was evaporated to provide the amine 20. ESI m/z: calculated for C_133_H_152_N_20_O_29_ [M+H]^+^ 2494.1; Found 2494.0.

Figure S60. ESI-MS spectrum of 20.

**Compound 21**

Compound 19 (250.0 mg, 0.09 mmol) and compound 20 (200.0 mg, 0.08 mmol) were dissolved in anhydrous 10 mL DMF, and then PyBop (94.0 mg, 0.18 mmol) and excess DIPEA (2 mL) were added into this reaction system. After stirring in room temperature 4 hours, rotary evaporating the solvent, the residues were dispersed in cold methanol, and then filtered the precipitant, compound 21 was obtained as white solid after drying in reduced pressure (150 mg, 32% yield). ^1^H NMR (400 MHz, Chloroform-*d*) δ 11.20 (s, 1H), 11.03 (s, 1H), 10.24 (d, *J* = 20.7 Hz, 2H), 9.90 (s, 1H), 9.85 (d, *J* = 9.4 Hz, 3H), 9.33 (d, *J* = 9.6 Hz, 1H), 9.02 (s, 1H), 8.69 (dd, *J* = 21.1, 8.7 Hz, 5H), 8.44 (d, *J* = 6.9 Hz, 1H), 7.93 – 7.80 (m, 3H), 7.73 (d, *J* = 8.2 Hz, 1H), 7.70 – 7.53 (m, 6H), 7.50 – 7.40 (m, 4H), 7.36 (t, *J* = 7.9 Hz, 2H), 7.28 – 7.09 (m, 10H), 7.09 – 7.00 (m, 8H), 6.93 (dd, *J* = 18.4, 10.3 Hz, 7H), 6.83 (d, *J* = 7.0 Hz, 1H), 6.66 (s, 1H), 6.61 – 6.50 (m, 2H), 6.48 – 6.34 (m, 10H), 6.32 – 6.20 (m, 11H), 6.17 – 6.00 (m, 11H), 5.30 – 5.18 (m, 2H), 4.91 (s, 1H), 4.65 (s, 1H), 4.55 (t, *J* = 10.5 Hz, 2H), 4.31 (s, 3H), 4.08 (d, *J* = 9.2 Hz, 13H), 4.01 – 3.86 (m, 12H), 3.84 (s, 3H), 3.81 – 3.40 (m, 48H), 3.35 – 3.00 (m, 8H), 1.61 (s, 18H), 1.57 – 1.50 (m, 44H), 1.43 (s, 6H), 1.35 – 1.24 (m, 52H).^13^C NMR (100 MHz, CDCl_3_) δ 165.53, 165.09, 164.30, 164.13, 164.02, 162.68, 161.89, 161.70, 161.39, 161.28, 160.91, 160.45, 160.07, 156.72, 156.55, 156.30, 155.74, 150.73, 150.38, 149.04, 146.71, 145.66, 145.35, 137.61, 137.12, 136.28, 136.01, 135.65, 135.48, 134.46, 133.90, 133.73, 133.23, 131.51, 131.25, 131.12, 130.57, 129.76, 128.41, 128.15, 127.79, 127.65, 127.00, 126.31, 126.20, 125.95, 125.85, 125.64, 125.55, 123.25, 122.14, 121.45, 121.38, 121.33, 121.26, 121.19, 120.97, 120.87, 120.72, 119.67, 118.92, 117.92, 117.88, 117.76, 117.70, 116.74, 116.57, 116.51, 116.41, 114.76, 114.71, 114.64, 114.50, 98.17, 98.09, 97.85, 97.75, 97.49, 97.22, 97.07, 79.98, 79.40, 79.32, 79.21, 79.14, 79.02, 72.22, 72.16, 72.12, 72.06, 71.99, 68.56, 68.08, 68.00, 67.92, 66.14, 66.00, 65.81, 53.73, 43.43, 39.97, 29.05, 29.01, 28.96, 28.92, 28.82, 28.79, 28.54, 22.32, 22.29, 22.26, 22.20, 22.17, 22.13, 0.04. ESI m/z: calculated for C_273_H_306_N_40_O_59_ [M+2H]**^2+^** 2545.1; Found 2545.0.

Figure S61. ^1^H NMR spectrum of 21 in CDCl_3_.

Figure S62. ^13^C NMR spectrum of 21 in CDCl_3_.

Figure S63. ESI-MS spectrum of compound 21.

**Compound (^m^Q^3^Q^2^)_8_**

The canonical Boc deprotection method was followed, compound 21 (20.0 mg, 0.0039 mmol) was dissolved in 1 mL anhydrous CH_2_Cl_2_ and then 2.3 μL trifluoroacetic acid (8 equiv.) was added into this reaction system. After stirring in room temperature 4 hours, rotary evaporating the solvent. The residues were washed by petroleum ether and diethyl ether, (^m^Q^3^Q^2^)_8_ was obtained after drying in reduced pressure. ^1^H NMR (400 MHz, Methanol-*d*_4_) δ 11.01 (s, 1H), 10.92 (s, 1H), 10.19 (s, 1H), 10.00 (s, 1H), 9.75 (t, *J* = 15.9 Hz, 4H), 9.58 (s, 1H), 9.15 (s, 2H), 8.96 (s, 1H), 8.85 (s, 1H), 8.70 (s, 2H), 8.23 (s, 1H), 7.93 (s, 1H), 7.85 – 7.73 (m, 9H), 7.67 (d, *J* = 7.6 Hz, 2H), 7.57 (s, 1H), 7.52 – 7.31 (m, 15H), 7.27 (s, 3H), 7.22 (d, *J* = 6.2 Hz, 3H), 7.15 (s, 3H), 7.09 (s, 5H), 6.94 (s, 8H), 6.83 (s, 2H), 6.77 – 6.66 (m, 5H), 6.63 – 6.58 (m, 3H), 6.47 (d, *J* = 8.1 Hz, 6H), 6.40 (s, 1H), 6.35 (s, 1H), 6.26 (d, *J* = 7.8 Hz, 8H), 6.17 (s, 3H), 4.63 (s, 2H), 4.31 – 4.27 (m, 4H), 4.06 – 3.93 (m, 21H), 3.87 – 3.82 (m, 13H), 3.74 – 3.65 (m, 14H), 3.57 (s, 8H), 3.52 – 3.46 (m, 12H), 3.24 (q, *J* = 7.6 Hz, 7H), 3.09 – 3.03 (m, 1H), 2.73 (s, 3H), 2.47 (d, *J* = 14.2 Hz, 2H), 2.26 (d, *J* = 36.2 Hz, 6H), 1.35 (d, *J* = 8.9 Hz, 48H). ESI m/z: calculated for C_233_H_242_N_40_O_43_ [M+2H]^2+^ 2146.3; Found 2145.9.

Figure S64. ^1^H NMR spectrum of (^m^Q^3^Q^2^)_8_ in Methanol-*d*_4_.

Figure S65. ESI-MS spectrum of compound (^m^Q^3^Q^2^)_8_.

**Compound 22**

Compound 9^[2]^ (10.0 g, 40 mmol), triphenylphosphine (PPh_3_, 11.2 g, 1.05 equiv.), 2-isopropoxyethan-1-ol (5.3g, 1.1 equiv.) were dissolved in 90 mL anhydrous tetrahydrofuran (THF), respectively. And then diisopropyl azodiformate (DIAD) was added dropwise at the condition of ice bath and the protection of nitrogen gas. After stirring for 4 hours at room temperature, rotary evaporating the solvent and then the residues were dispersed in methanol to recrystallize in refrigerator at -20°C for one night. After filtering the precipitant with a Buchner funnel, Compound 22 was obtained as white solid (11.0 g, 80% yield). ^1^H NMR (400 MHz, Chloroform-*d*) δ 8.50 (dd, *J* = 8.5, 1.5 Hz, 1H), 8.12 (dd, *J* = 7.5, 1.5 Hz, 1H), 7.71 (s, 1H), 7.67 (t, *J* = 8.0 Hz, 1H), 4.48 (t, *J* = 4.7 Hz, 2H), 4.05 (s, 3H), 3.97 (dd, *J* = 5.4, 3.9 Hz, 2H), 3.75 (p, *J* = 6.1 Hz, 1H), 1.25 (d, *J* = 6.1 Hz, 6H). ^13^C NMR (100 MHz, CDCl_3_) δ 165.63, 162.74, 151.26, 148.33, 140.07, 126.64, 126.01, 125.22, 123.27, 102.46, 72.47, 69.32, 65.92, 53.44, 22.10, 0.05. ESI m/z: calculated for C_16_H_18_N_2_O_6_ [M+H]^+^ 335.1; Found 335.1.

 Figure S66. ^1^H NMR spectrum of 22 in CDCl_3_.

 Figure S67. ^1^C NMR spectrum of 22 in CDCl_3_.

Figure S68. ESI-MS spectrum of compound 22.

**Compound 23**

Compound 22 (5.0 g, 14.9 mmol) and ammonium formate (1.9 g, 2.0 equiv.) were dissolved in the mixture solution of EtOAc (25 mL) and MeOH (25), and 25 mg of 10 wt % Pd/C was added. The reaction was stirred at room temperature for 4 hours. Upon completion the catalyst was removed by filtration over celite and the filtrate was evaporated to provide the amine 23 with quantitative yield. ^1^H NMR (400 MHz, Chloroform-*d*) δ 7.56 – 7.49 (m, 2H), 7.38 (t, *J* = 7.9 Hz, 1H), 6.95 (dd, *J* = 7.5, 1.3 Hz, 1H), 5.18 (s, 2H), 4.39 (dd, *J* = 5.7, 4.1 Hz, 2H), 4.04 (s, 3H), 3.95 (t, *J* = 4.9 Hz, 2H), 3.77 (p, *J* = 6.1 Hz, 1H), 1.25 (d, *J* = 6.1 Hz, 6H). ^13^C NMR (100 MHz, CDCl_3_) δ 166.34, 162.44, 145.68, 144.94, 138.41, 128.74, 122.95, 110.97, 109.71, 100.96, 77.45, 77.13, 76.81, 72.40, 68.55, 66.11, 52.88, 22.15. ESI m/z: calculated for C_16_H_20_N_2_O_4_ [M+H]^+^ 305.1; Found 305.1.

Figure S69. ^1^H NMR spectrum of 23 in CDCl_3_.

Figure S70. ^1^C NMR spectrum of 23 in CDCl_3_.

Figure S71. ESI-MS spectrum of compound 23.

**Compound 24**

Compound 23 (3.9 g, 12.8 mmol) and N-α-benzyloxycarbonyl-N-δ-boc-L-ornithine (5.2 g, 1.1 eq) were dissolved in the mixture solution of MeCN (65 mL). NMI (3.5 eq) was added, followed by TCFH (1.2 eq). The reaction was stirred at room temperature for 20 hours, and compound 24 was purified by silica gel chromatography as yellow solid (5.0 g, 60% yield). ^1^H NMR (400 MHz, Chloroform-*d*) δ 10.34 (s, 1H), 8.76 (d, *J* = 7.8 Hz, 1H), 7.96 – 7.90 (m, 1H), 7.59 (d, *J* = 5.9 Hz, 2H), 7.37 (s, 2H), 7.34 – 7.29 (m, 2H), 7.26 (d, *J* = 2.5 Hz, 2H), 5.81 (s, 1H), 5.16 (s, 2H), 4.71 (s, 1H), 4.62 (s, 1H), 4.42 (t, *J* = 4.8 Hz, 2H), 3.99 (s, 3H), 3.95 (t, *J* = 4.6 Hz, 2H), 3.75 (q, *J* = 5.5, 5.0 Hz, 1H), 3.19 (s, 2H), 2.11 – 2.00 (m, 1H), 1.90 – 1.78 (m, 1H), 1.40 (s, 9H), 1.24 (d, *J* = 5.6 Hz, 6H). ^13^C NMR (100 MHz, DMSO) δ 171.46, 165.50, 162.96, 157.14, 156.10, 147.71, 138.65, 137.49, 136.41, 135.04, 130.56, 128.87, 128.72, 128.24, 127.96, 123.52, 121.89, 120.69, 117.40, 115.93, 102.35, 77.91, 71.74, 69.68, 66.29, 66.10, 56.70, 53.21, 40.62, 40.41, 40.20, 40.00, 39.79, 39.58, 39.37, 35.79, 28.75, 26.75, 22.56. ESI m/z: calculated for C_34_H_44_N_4_O_9_ [M+H]^+^ 653.3; Found 653.3.

Figure S72. ^1^H NMR spectrum of 24 in CDCl_3_.

Figure S73. ^1^C NMR spectrum of 24 in DMSO-d_6_.

Figure S74. ESI-MS spectrum of compound 24.

**Compound 25**

Compound 24 (4.8g, 7.3 mmol) and LiOH (2.0 equiv.) were added to methanol and stirred for 2 hours, and rotary evaporated the solvent. Then acidification and extraction were carried out respectively. Compound 25 as faint yellow powder (4.4 g, 90% yield) after reduced pressure drying. ^1^H NMR (400 MHz, DMSO-*d*_6_) δ 10.61 (s, 1H), 8.77 (d, *J* = 7.8 Hz, 1H), 7.92 (d, *J* = 7.6 Hz, 1H), 7.86 (d, *J* = 8.3 Hz, 1H), 7.68 (d, *J* = 7.7 Hz, 2H), 7.36 (dt, *J* = 24.8, 8.5 Hz, 5H), 6.83 (s, 1H), 5.15 – 5.03 (m, 2H), 4.48 (t, *J* = 4.5 Hz, 3H), 3.89 (t, *J* = 4.5 Hz, 2H), 3.73 (p, *J* = 6.0 Hz, 1H), 2.95 (d, *J* = 6.4 Hz, 2H), 1.82 (s, 1H), 1.54 (s, 2H), 1.35 (s, 9H), 1.16 (d, *J* = 6.1 Hz, 6H). ^13^C NMR (100 MHz, CDCl_3_) δ 170.07, 163.59, 157.87, 156.49, 135.65, 134.87, 129.78, 128.50, 128.28, 127.98, 122.39, 118.43, 116.45, 100.23, 79.57, 77.40, 77.08, 76.76, 72.49, 69.10, 67.96, 66.01, 55.48, 40.01, 28.43, 27.15, 26.79, 22.12, 0.05. ESI m/z: calculated for C_33_H_42_N_4_O_9_ [M+H]^+^ 639.3; Found 639.3.

Figure S75. ^1^H NMR spectrum of 25 in DMSO-d_6_.

Figure S76. ^1^C NMR spectrum of 25 in CDCl_3_.

Figure S77. ESI-MS spectrum of compound 25.

**Compound 26**

Compound 25 (3.9 g, 6.0 mmol) and compound 23 (2.2 g, 1.2 equiv.) were dissolved in the mixture solution of MeCN (45 mL). NMI (3.5 eq) was added, followed by TCFH (1.2 equiv.). The reaction was stirred at room temperature for 20 hours, and compound 26 purified by silica gel chromatography as yellow solid (4.0 g, 70% yield). ^1^H NMR (400 MHz, Chloroform-*d*) δ 11.83 (s, 1H), 10.73 (s, 1H), 8.88 (d, *J* = 7.5 Hz, 2H), 7.99 (t, *J* = 8.2 Hz, 2H), 7.81 (s, 1H), 7.66 (t, *J* = 8.1 Hz, 1H), 7.61 (t, *J* = 8.1 Hz, 1H), 7.49 (s, 1H), 7.20 – 7.05 (m, 3H), 6.73 (d, *J* = 7.2 Hz, 2H), 6.67 (d, *J* = 8.8 Hz, 1H), 4.82 (d, *J* = 12.5 Hz, 1H), 4.60 (s, 1H), 4.49 (q, *J* = 8.1, 4.8 Hz, 3H), 4.40 (tq, *J* = 10.4, 5.1 Hz, 2H), 4.19 (d, *J* = 12.9 Hz, 1H), 3.98 (q, *J* = 4.7, 3.7 Hz, 7H), 3.77 (dq, *J* = 12.4, 6.1 Hz, 2H), 3.08 (s, 2H), 2.26 (s, 1H), 2.00 – 1.88 (m, 1H), 1.54 (d, *J* = 7.5 Hz, 2H), 1.41 (s, 9H), 1.26 (t, *J* = 6.0 Hz, 12H). ^13^C NMR (100 MHz, CDCl_3_) δ 170.83, 166.39, 163.48, 163.16, 162.89, 156.59, 155.91, 150.46, 146.73, 140.01, 138.64, 136.17, 134.80, 134.02, 128.34, 128.15, 127.83, 127.75, 127.36, 122.27, 122.15, 119.07, 118.21, 116.75, 116.67, 101.54, 99.48, 79.11, 77.43, 77.11, 76.79, 72.50, 72.48, 68.96, 68.89, 66.40, 66.09, 66.06, 56.11, 53.42, 40.07, 29.40, 28.45, 26.96, 26.67, 22.18, 0.07. ESI m/z: calculated for C_49_H_60_N_6_O_12_ [M+H]^+^ 925.4; Found 925.4.

Figure S78. ^1^H NMR spectrum of 26 in CDCl_3_.

Figure S79. ^1^C NMR spectrum of 26 in CDCl_3_.

Figure S80. ESI-MS spectrum of compound 26.

**Compound 27**

Compound 26 (2.0 g, 2.1 mmol) and LiOH (96.0 mg, 4.0 mmol) were added to methanol and stirred for 2 hours, and rotary evaporate the solvent. Then acidification and extraction were carried out respectively. Compound 27 as yellow powder (1.8 g, 92% yield) was used without further purification after reduced pressure drying. ESI m/z: calculated for C_48_H_58_N_6_O_12_ [M+H]^+^ 911.4; Found 911.4.

Figure S81. ESI-MS spectrum of compound 27.

**Compound 28**

The compound 26 (2.0 g, 2.1 mmol) and ammonium formate (315.0 mg, 5.0 mmol) were dissolved in the mixture solution of DCM (10 mL) and MeOH (10 mL), and 25 mg of 10 wt % Pd/C was added. The reaction was stirred at rt for 4 hours. Upon completion the catalyst was removed by filtration over celite and the filtrate was evaporated to provide the amine 28 that was used without further purification. ^1^H NMR (400 MHz, Chloroform-*d*) δ 11.95 (s, 1H), 11.81 (s, 1H), 8.91 (dd, *J* = 7.8, 1.3 Hz, 1H), 8.83 (dd, *J* = 7.7, 1.3 Hz, 1H), 8.03 (dd, *J* = 8.5, 1.4 Hz, 1H), 7.96 (dd, *J* = 8.4, 1.3 Hz, 1H), 7.78 (s, 1H), 7.71 (t, *J* = 8.1 Hz, 1H), 7.65 – 7.56 (m, 2H), 4.63 (s, 1H), 4.51 – 4.43 (m, 4H), 3.98 (d, *J* = 6.8 Hz, 7H), 3.77 (pd, *J* = 6.1, 2.0 Hz, 2H), 3.61 (dd, *J* = 7.8, 4.3 Hz, 1H), 3.09 (d, *J* = 6.7 Hz, 2H), 1.98 (s, 1H), 1.63 (d, *J* = 7.3 Hz, 1H), 1.57 (q, *J* = 8.6, 7.6 Hz, 2H), 1.38 (s, 9H), 1.25 (d, *J* = 6.0 Hz, 12H). ^13^C NMR (100 MHz, CDCl_3_) δ 174.14, 165.76, 163.53, 163.50, 163.10, 155.95, 150.33, 147.03, 140.13, 138.88, 135.03, 134.49, 128.49, 127.98, 122.48, 122.24, 119.58, 117.47, 116.72, 116.14, 101.74, 99.26, 79.13, 77.40, 77.08, 76.76, 72.52, 72.46, 68.99, 68.94, 66.07, 56.02, 53.39, 40.31, 32.04, 28.43, 26.52, 22.16, 0.06. ESI m/z: calculated for C_41_H_54_N_6_O_10_ [M+H]^+^ 791.4; Found 791.4.

Figure S82. ^1^H NMR spectrum of 28 in CDCl_3_.

Figure S83. ^1^C NMR spectrum of 28 in CDCl_3_.

Figure S84. ESI-MS spectrum of compound 28.

**Compound 29**

Dry N,N-diisopropylethylamine (2.0 mL, 11.8 mmol) was added to a solution of 27 (1.5 g, 1.9 mmol), HBTU (1.5 g, 4.0 mmol) and HOBt (81 mg, 0.6 mmol) in dry dimethylformamide (10 mL) under N_2_, and stirred for 10 minutes at room temperature. Then, 28 (1.4 g, 1.8 mmol) in dry dimethylformamide (10 mL) was added to the reaction mixture, and stirred for 14 hours at room temperature. The reaction mixture was quenched with 5% aqueous citric acid solution and extracted with dichloromethane. The organic layer was washed with water and brine, dried over magnesium sulfate and filtered. After the solvent was removed in vacuo, the residue was purified by silica gel to give 29 (2.0 g, 60%) as a yellow solid. ^1^H NMR (400 MHz, Acetonitrile-*d*_3_) δ 11.03 (s, 1H), 11.00 (s, 1H), 10.37 (s, 1H), 10.09 (s, 1H), 8.75 (d, *J* = 9.6 Hz, 1H), 8.48 (d, *J* = 7.8 Hz, 1H), 8.37 (dd, *J* = 17.5, 7.6 Hz, 2H), 7.53 (d, *J* = 23.1 Hz, 3H), 7.47 (q, *J* = 8.0, 5.6 Hz, 5H), 7.34 (td, *J* = 8.0, 5.2 Hz, 3H), 7.29 – 7.19 (m, 3H), 7.18 – 6.91 (m, 7H), 6.58 (d, *J* = 7.6 Hz, 2H), 6.48 (d, *J* = 10.0 Hz, 2H), 5.53 (t, *J* = 5.8 Hz, 1H), 4.93 (q, *J* = 7.6 Hz, 1H), 4.38 – 4.25 (m, 5H), 4.17 (d, *J* = 8.4 Hz, 2H), 4.06 (d, *J* = 8.4 Hz, 1H), 4.00 – 3.85 (m, 12H), 3.79 (q, *J* = 7.4, 6.0 Hz, 9H), 3.74 (d, *J* = 5.9 Hz, 1H), 3.18 – 3.01 (m, 3H), 2.32 (s, 2H), 2.07 – 1.99 (m, 2H), 1.57 (t, *J* = 8.9 Hz, 4H), 1.42 (d, *J* = 17.8 Hz, 25H), 1.34 – 1.23 (m, 33H), 1.22 (s, 8H), 0.95 – 0.79 (m, 2H). ^13^C NMR (100 MHz, CDCl_3_) δ 176.86, 170.22, 170.09, 165.62, 165.30, 162.95, 162.58, 162.33, 161.94, 157.30, 157.25, 156.34, 156.05, 150.15, 149.09, 147.27, 139.66, 138.59, 137.65, 137.45, 135.76, 134.22, 133.64, 133.36, 133.23, 130.03, 129.78, 128.22, 127.99, 127.81, 127.69, 127.40, 127.28, 126.95, 126.80, 126.54, 125.56, 123.26, 121.71, 121.21, 121.11, 118.92, 118.50, 117.82, 117.26, 116.60, 115.58, 101.29, 99.29, 98.71, 98.12, 97.37, 79.04, 77.44, 77.12, 76.81, 72.45, 72.37, 72.32, 68.88, 68.74, 68.57, 68.34, 66.76, 66.13, 66.06, 66.00, 55.71, 54.18, 52.90, 40.39, 40.19, 36.60, 33.88, 31.96, 30.86, 30.42, 29.82, 29.75, 29.58, 29.38, 29.23, 29.16, 28.50, 28.43, 28.37, 27.26, 27.22, 27.03, 26.73, 24.84, 22.74, 22.32, 22.27, 22.24, 22.21, 22.18, 14.20, 0.07. ESI m/z: calculated for C_89_H_110_N_12_O_21_ [M+H]^+^ 1683.8; Found 1683.8.

Figure S85. ^1^H NMR spectrum of 29 in CD_3_CN.

Figure S86. ^1^C NMR spectrum of 29 in CDCl_3_.

Figure S87. ESI-MS spectrum of compound 29.

**Compound 30**

Compound 29 (1.0 g, 0.6 mmol) and LiOH (48.0 mg, 2.0 mmol) were added to methanol and stirred for 2 hours, and rotary evaporated the solvent. Then acidification and extraction were carried out respectively. Compound 30 as yellow powder (880.0 mg, 88% yield) was used without further purification after reduced pressure drying. ESI m/z: calculated for C_88_H_108_N_12_O_21_ [M+H]^+^ 1669.8; Found 1669.8.

Figure S88. ESI-MS spectrum of compound 30.

**Compound 31**

The compound 29 (1.0 g, 0.59 mmol) and ammonium formate (126.0 mg, 2.0 mmol) were dissolved in the mixture solution of DCM (10 mL) and MeOH (10 mL), and 25 mg of 10 wt % Pd/C was added. The reaction was stirred at rt for 4 hours. Upon completion the catalyst was removed by filtration over celite and the filtrate was evaporated to provide the amine 31 that was used without further purification. ^1^H NMR (400 MHz, Acetonitrile-*d*_3_) δ 11.50 (s, 1H), 10.92 (s, 2H), 10.25 (s, 1H), 8.84 (s, 1H), 8.47 (q, *J* = 4.8 Hz, 1H), 8.22 (d, *J* = 40.2 Hz, 2H), 7.52 (s, 2H), 7.46 (d, *J* = 8.7 Hz, 2H), 7.42 – 7.38 (m, 4H), 7.35 – 7.26 (m, 4H), 7.13 (t, *J* = 8.1 Hz, 1H), 6.99 (t, *J* = 8.0 Hz, 1H), 6.91 (d, *J* = 6.4 Hz, 1H), 6.71 (s, 1H), 6.37 (s, 1H), 5.66 (s, 1H), 5.35 (t, *J* = 5.8 Hz, 1H), 4.88 (q, *J* = 7.4 Hz, 1H), 4.48 – 4.30 (m, 5H), 3.92 – 3.74 (m, 22H), 3.35 (d, *J* = 7.7 Hz, 1H), 2.93 (s, 2H), 2.26 (s, 4H), 1.46 (s, 9H), 1.43 – 1.38 (m, 8H), 1.30 (s, 9H), 1.24 (s, 8H), 1.20 (d, *J* = 6.0 Hz, 8H). ^13^C NMR (100 MHz, CDCl_3_) δ 173.10, 169.96, 165.23, 162.89, 162.75, 162.28, 162.21, 162.08, 156.04, 150.33, 149.29, 146.94, 139.09, 138.53, 137.76, 134.14, 133.43, 133.21, 128.30, 128.07, 127.87, 127.51, 127.22, 126.88, 126.43, 121.88, 121.27, 121.16, 117.89, 117.55, 117.28, 116.70, 116.34, 115.87, 101.41, 98.94, 97.95, 97.37, 79.09, 78.90, 77.45, 77.13, 76.81, 72.38, 72.31, 72.25, 68.82, 68.68, 68.37, 66.11, 66.02, 55.75, 54.58, 52.85, 40.34, 40.01, 32.10, 30.08, 28.50, 28.45, 28.35, 26.78, 26.30, 22.29, 22.24, 22.20, 22.17, 0.07. ESI m/z: calculated for C_81_H_104_N_12_O_19_ [M+H]^+^ 1549.8; Found 1549.7.

Figure S89. ^1^H NMR spectrum of 31 in CD_3_CN.

Figure S90. ^1^C NMR spectrum of 31 in CDCl_3_.

Figure S91. ESI-MS spectrum of compound 31.

**Compound 32**

Dry N,N-diisopropylethylamine (0.5 mL, 2.7 mmol) was added to a solution of 30 (0.8 g, 0.47 mmol), HBTU (375.0 mg, 1.0 mmol) and HOBt (21.0 mg, 0.2 mmol) in dry dimethylformamide (5 mL) under N_2_, and stirred for 10 minutes at room temperature. Then, 31 (620.0 mg, 0.4 mmol) in dry dimethylformamide (5 mL) was added to the reaction mixture, and stirred for 24 hours at room temperature. The reaction mixture was quenched with 5% aqueous citric acid solution and extracted with dichloromethane. The organic layer was washed with water and brine, dried over magnesium sulfate and filtered. After the solvent was removed in vacuo, the residue was purified by silica gel to give 32 (400.0 mg, 26%) as a yellow solid. ^1^H NMR (400 MHz, Acetonitrile-*d*_3_) δ 10.46 (d, *J* = 15.3 Hz, 2H), 10.20 (s, 1H), 10.09 (s, 1H), 9.89 (s, 1H), 9.74 (d, *J* = 11.2 Hz, 2H), 9.59 (s, 1H), 8.54 (s, 1H), 8.19 (s, 1H), 7.99 (s, 1H), 7.78 (s, 2H), 7.58 – 7.48 (m, 4H), 7.43 – 7.36 (m, 4H), 7.31 – 7.15 (m, 8H), 7.04 (t, *J* = 10.5 Hz, 8H), 6.94 (d, *J* = 7.4 Hz, 4H), 6.76 – 6.61 (m, 2H), 6.45 (s, 2H), 6.34 (s, 2H), 6.09 (s, 2H), 5.78 (s, 1H), 5.71 – 5.59 (m, 2H), 4.82 (d, *J* = 12.5 Hz, 1H), 4.70 (s, 1H), 4.59 (s, 1H), 4.37 (s, 6H), 4.30 – 4.20 (m, 3H), 4.18 – 3.97 (m, 14H), 3.95 – 3.77 (m, 26H), 3.68 (dt, *J* = 12.1, 6.1 Hz, 6H), 3.56 (s, 5H), 3.11 – 2.89 (m, 10H), 1.51 – 1.46 (m, 18H), 1.41 (d, *J* = 10.7 Hz, 18H), 1.35 – 1.31 (m, 17H), 1.23 (d, *J* = 6.1 Hz, 16H), 1.15 (t, *J* = 6.6 Hz, 16H). ^13^C NMR (125 MHz, CDCl_3_) δ 169.37, 166.13, 165.42, 162.83, 162.06, 156.08, 151.34, 148.95, 140.02, 139.33, 139.04, 134.10, 133.17, 129.73, 128.06, 127.35, 126.24, 121.16, 120.69, 118.02, 116.44, 99.28, 98.46, 78.95, 77.30, 77.04, 76.79, 72.27, 68.68, 66.71, 66.02, 62.75, 53.82, 47.15, 40.05, 31.92, 29.70, 29.35, 28.44, 28.37, 26.74, 22.68, 22.35, 22.27, 22.16, 22.07, 14.12, 0.00. ESI m/z: calculated for C_169_H_210_N_24_O_39_ [M+2H^+^]^2+^ 1600.7; Found 1600.7.

Figure S92. ^1^H NMR spectrum of 32 in CD_3_CN.

Figure S93. ^1^C NMR spectrum of 32 in CDCl_3_.

Figure S94. ESI-MS spectrum of compound 32.

**Compound 33**

Compound 32 (0.1 g, 0.03 mmol) and LiOH (10.0 mg) were added to methanol and stirred for 2 hours, and rotary evaporated the solvent. Then acidification and extraction were carried out respectively. Compound 33 as yellow powder (0.08 g, 83% yield) was used without further purification after reduced pressure drying. ESI m/z: calculated for C_168_H_208_N_24_O_39_ [M+2H]^2+^ 1593.7; Found 1593.7.

Figure S95. ESI-MS spectrum of compound 33.

**Compound 34**

The compound 32 (0.8 mg, 0.03 mmol) and ammonium formate (30.0 mg, 0.5 mmol) were dissolved in the mixture solution of DCM (5 mL) and MeOH (5 mL), and 10 mg of 10 wt % Pd/C was added. The reaction was stirred at room temperature for 24 hours. Upon completion the catalyst was removed by filtration over celite and the filtrate was evaporated to provide the amine 34 (0.7 mg, 76% yield) that was used without further purification. ESI m/z: calculated for C_161_H_204_N_24_O_37_ [M+2H]^2+^ 1534.2; Found 1534.2

Figure S96. ESI-MS spectrum of compound 34.

**Compound 35**

Dry N,N-diisopropylethylamine (30.0 μL, 0.145 mmol) was added to a solution of 33 (90.0 mg, 0.029 mmol), HBTU (5.0 mg, 0.013 mmol) and HOBt (1.0 mg, 0.01 mmol) in dry dimethylformamide (5 mL) under N_2_, and stirred for 10 minutes at room temperature. Then, 34 (80.0 mg, 0.026 mmol) in dry dimethylformamide (5 mL) was added to the reaction mixture, and stirred for 48 hours at room temperature. Then another HBTU (5.0 mg, 0.013 mmol), dry N,N-diisopropylethylamine (30.0 μL, 0.145 mmol) and HOBt (1.0 mg, 0.01 mmol) in dry dimethylformamide (5 mL) were added under N_2_ for another 24 hours. The reaction mixture was quenched with 5% aqueous citric acid solution and extracted with dichloromethane. The organic layer was washed with water and brine, dried over magnesium sulfate and filtered. After the solvent was removed in vacuo, the residue was purified by silica gel, the residues were dispersed in diethyl ether and DCM, and then filter the precipitant to give 35 (30.0 mg, 24% yield) as a yellow solid. ^1^H NMR (500 MHz, Chloroform-*d*) δ 11.00 (d, *J* = 34.6 Hz, 2H), 10.82 – 9.51 (m, 14H), 9.13 – 7.31 (m, 42H), 7.26 – 5.98 (m, 42H), 5.51 – 3.38 (m, 113H), 3.06 (d, *J* = 52.9 Hz, 21H), 2.26 (d, *J* = 67.2 Hz, 6H), 1.95 – 1.71 (m, 8H), 1.33 – 0.84 (m, 168H). ESI m/z: calculated for C_329_H_410_N_48_O_75_ [M+3H]^3+^ 2078.6; Found 2078.6.

Figure S97. ^1^H NMR spectrum of 35 in CDCl_3_.
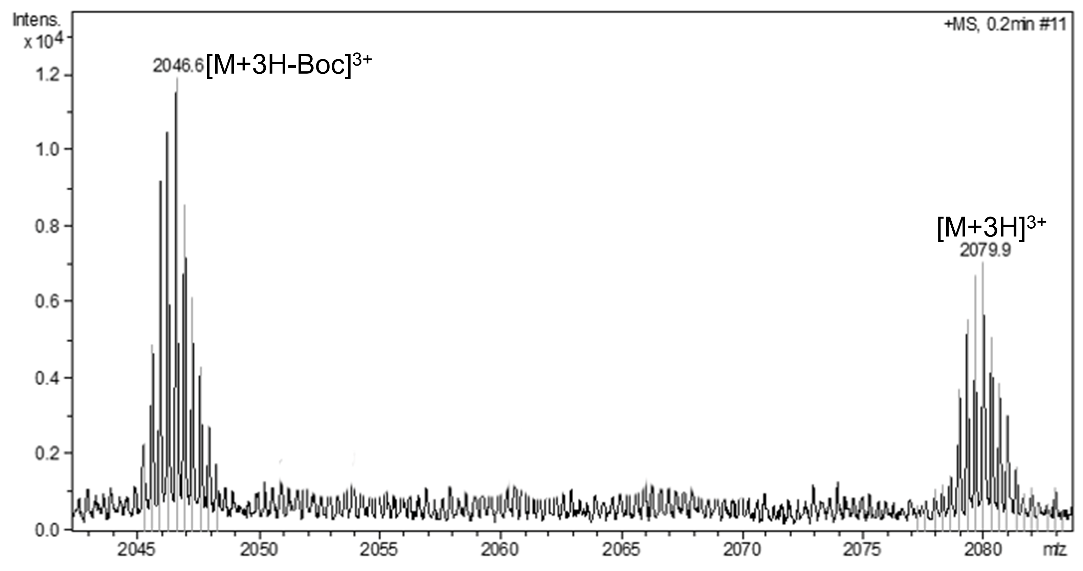


Figure S98. ESI-MS spectrum of compound 35.

**Compound (A^Orn^Q^3^Q^3^)_8_**

The canonical Boc deprotection method was followed, compound 35 (10.0 mg, 0.0016 mmol) was dissolved in 500 μL anhydrous CH_2_Cl_2_ and then 1.0 μL trifluoroacetic acid (8 equiv.) was added into this reaction system. After stirring in room temperature 4 hours, rotary evaporating the solvent. The residues were washed by petroleum ether and diethyl ether, (A^Orn^Q^3^Q^3^)_8_ was obtained after drying in reduced pressure. ESI m/z: calculated for C_289_H_346_N_48_O_59_ [M+2H]^2+^ 2719.1; Found 2719.3.

Figure S99. ESI-MS spectrum of compound (A^Orn^Q^3^Q^3^)_8_.

**Reference**

[1] a) Z. L. Xi-Zeng Feng, Lin-Jin Yang, Chen Wang, Chun-li Bai *Talanta.* **1998**, *47*, 1223-1229; b) A. Manna, S. Chakravorti, *Spectrochim Acta A Mol Biomol Spectrosc.* **2015**, *150*, 120-126.

[2] K. Ziach, C. Chollet, V. Parissi, P. Prabhakaran, M. Marchivie, V. Corvaglia, P. P. Bose, K. Laxmi-Reddy, F. Godde, J.-M. Schmitter, S. Chaignepain, P. Pourquier, I. Huc, *Nature Chemistry.* **2018**, *10*, 511-518.

[3] T. Qi, T. Deschrijver, I. Huc, *Nat. Protoc.* **2013**, *8*, 693-708.
